# Safety and efficacy of leriglitazone in childhood cerebral adrenoleukodystrophy (NEXUS): an interim analysis of an open-label, phase 2/3 trial

**DOI:** 10.1016/j.eclinm.2025.103265

**Published:** 2025-05-24

**Authors:** Ángeles García-Cazorla, Caroline Sevin, Juliana Ribeiro Constante, Elise Yazbeck, Hendrik Rosewich, Sandra Jimenez, Gloria Chia-Yi Chiang, Otto Rapalino, Paul Caruso, Daniel Balentine, Karl G. Helmer, Seth Bennett, Marco Emanuele, Laura Rodriguez-Pascau, Pilar Pizcueta, Guillem Pina, Anna Vilà, Maria Rovira, Adriana Mantilla, Uwe Meya, Arun Mistry, María Pascual, Sílvia Pascual, Marc Martinell, Patricia L. Musolino, Eric Mallack

**Affiliations:** a Neurometabolic Unit, Neurology Department, Institut de Recerca, CIBERER and MetabERN, Hospital Sant Joan de Déu, Barcelona, Spain; b Pediatric Neurology Department, Assistance Publique-Hôpitaux de Paris, Hôpitaux Universitaires Paris Saclay, Bicêtre Hospital, Le Kremlin Bicêtre, Paris, France; c Department of Pediatrics and Adolescent Medicine, University Medical Center Göttingen, Georg August University Göttingen, Göttingen, Germany; d German Center for Child and Adolescent Health (DZKJ), Göttingen, Germany; e Department of Child Neurology, Developmental Neurology, General Pediatrics, Endocrinology, Diabetology, Social Pediatrics, University Children's Hospital, Eberhard Karls University Tübingen, Tübingen, Germany; f Children's Neurodevelopment Center, Hasbro Children's Hospital, Providence, RI, USA; g Department of Radiology, Weill Cornell Medicine, NewYork-Presbyterian Hospital, New York, NY, USA; h Division of Neuroradiology, Massachusetts General Hospital, Harvard Medical School, Boston, MA, USA; i Pediatric Neuroimaging Service, Lenox Hill Radiology and Medical Imaging Associates, New York, NY, USA; j Athinoula A. Martinos Center for Biomedical Imaging, Department of Radiology, Massachusetts General Hospital, Boston, MA, USA; k Harvard Medical School, Boston, MA, USA; l CTI Clinical Trial & Consulting, Covington, KY, USA; m Minoryx Therapeutics SL, Barcelona, Spain; n Department of Neurology, Massachusetts General Hospital, Harvard Medical School, Boston, MA, USA; o The Moser Center for Leukodystrophies, Kennedy Krieger Institute, Johns Hopkins University School of Medicine, Baltimore, MD, USA

**Keywords:** Cerebral adrenoleukodystrophy, Paediatric adrenoleukodystrophy, PPARγ agonist

## Abstract

**Background:**

Cerebral adrenoleukodystrophy rapidly progresses in approximately 90% of untreated patients. Current treatment, haematopoietic stem-cell transplantation (HSCT), is associated with high morbidity and is not widely available. Lower risk treatments that can be administered immediately upon lesion identification are needed. Leriglitazone, a peroxisome proliferator-activated receptor gamma agonist, may slow disease progression.

**Methods:**

NEXUS (NCT04528706), a 96-week, phase 2/3, open-label, multicentre study conducted between February 13, 2020 and April 2025, enrolled boys aged 2–12 years with X-linked adrenoleukodystrophy with white matter lesions. Participants received oral leriglitazone once-daily. The primary endpoint is the proportion of patients with arrested disease at week 96. This predefined interim analysis assessed the continuation criteria at week 24, defined as the proportion of patients with lesion growth deceleration or disease arrest (success: one-sided 95% CI >10%). Secondary endpoints were the change from baseline in neurologic function score (NFS), Loes score and gadolinium intensity score (GIS), the overall survival of patients remaining on leriglitazone, and the number of patients meeting study HSCT criteria.

**Findings:**

Eleven patients were evaluable at week 24 and all met the continuation criteria. All remained clinically stable and showed lesion growth deceleration. Five (45%, 95% CI 16·7–76·6) had arrested disease. NFS, Loes score, and GIS stabilised by week 24 in most patients. Survival of patients who remained on leriglitazone was 100% (95% CI 69·2–100·0). Five patients met the study HSCT criteria owing to persistent gadolinium enhancement but had no significant lesion growth. Leriglitazone was well tolerated; 87 adverse events occurred and there were no treatment-related serious adverse events.

**Interpretation:**

All evaluable patients met the continuation criteria. Clinical and radiological data suggest deceleration of disease progression compared with available natural history data, indicating that leriglitazone may be beneficial in boys with cerebral adrenoleukodystrophy. Additional follow-up will fully assess the safety and efficacy of leriglitazone in cerebral adrenoleukodystrophy.

**Funding:**

Minoryx Therapeutics.

**Research in context**

**Evidence before this study**

We searched PubMed from inception to September 1, 2023, using the terms ‘adrenoleukodystrophy’ AND (‘treatment’ OR ‘therapy’ OR ‘drug’) AND (‘clinical trial’ OR ‘trial’ OR ‘randomised’). We screened search results to only include publications reporting studies of paediatric patients with cerebral adrenoleukodystrophy receiving drug therapy, allogeneic haematopoietic stem-cell transplantation (HSCT) or autologous-modified HSCT (gene therapy). Studies of adult patients, dietary supplements, non-interventional trials, and non-clinical data were excluded.

In total, 11 publications were included in this review of previous evidence; most studies were retrospective or reported small numbers of patients. All four studies of drug therapy for children with cerebral adrenoleukodystrophy reported disease progression in clinical, radiological, or biomarker outcomes and no alteration in the natural history. Allogeneic or autologous-modified HSCT treatments in children with cerebral adrenoleukodystrophy were reported by five and two publications, respectively. Survival was reported to be greater for patients who underwent HSCT than those who did not; however, in some cases, patients continued to deteriorate after HSCT, with disease progression generally correlating with the extent of cerebral disease at the time of transplant. One study reported a transplantation-related mortality rate of 8% in patients who underwent allogeneic HSCT.

**Added value of this study**

This literature review highlights the lack of drug treatments for children with cerebral adrenoleukodystrophy, and for those treatments that are under development, there is a paucity of clinical trial data. Studies of allogeneic and autologous-modified HSCT demonstrate that while these treatments are effective in some patients, success is dependent on the extent of cerebral disease at the time of transplant. Therefore, an unmet need exists for a drug treatment that can alter the course of disease. NEXUS is the first multicentre study of a drug-based treatment for boys with cerebral adrenoleukodystrophy.

**Implications of all the available evidence**

This interim analysis of the NEXUS study suggests early changes in the natural history of cerebral adrenoleukodystrophy in patients receiving leriglitazone for 24 weeks. Although full study follow-up to 96 weeks is required to assess efficacy and safety, these interim data support the potential of leriglitazone in the treatment of childhood cerebral adrenoleukodystrophy.

## Introduction

Adrenoleukodystrophy is a rare, X-linked neurodegenerative disease caused by mutations in the *ABCD1* gene, leading to the accumulation of very long-chain fatty acids in the brain, spinal cord, adrenal glands, and other tissues.^1^^,^^2^ Adrenoleukodystrophy presents with two nervous system phenotypes: adrenomyeloneuropathy and cerebral adrenoleukodystrophy.^3^ Adrenomyeloneuropathy is an adult-onset, chronic disorder characterised by axonal damage in the spinal cord and peripheral nervous system that affects all male and most female patients with adrenoleukodystrophy. Cerebral adrenoleukodystrophy, mainly occurring in males, is characterised by demyelinating brain lesions; once these lesions begin growing the disease is considered to be progressive and is usually fatal within 10 years.^1^^,^^2^ For patients with adrenoleukodystrophy, there is a 60% lifetime risk of developing cerebral lesions. Most cases occur in boys between 3 and 12 years of age.^4^^,^^5^ In approximately 10% of patients, lesions spontaneously self-arrest, most often in adolescence and rarely in children below 10 years of age.^6^

Current treatments for cerebral adrenoleukodystrophy are allogeneic haematopoietic stem-cell transplantation (HSCT) or, in the USA only, autologous-modified HSCT (*ex vivo* gene therapy, elivaldogene autotemcel). Boys are generally considered eligible for HSCT if they have a Loes score below 9, a neurologic function score (NFS) of 0 or 1, and growing lesions with gadolinium enhancement.^7^^,^^8^ Although allogeneic HSCT and autologous-modified HSCT may halt disease progression,^2^^,^^9^, ^10^, ^11^, ^12^ there are significant limitations associated with both treatments. Suitable donors for allogeneic HSCT are not always available,^13^ when available, there is a delay of 3–4 months of untreated disease progression between the detection of gadolinium-enhancing lesions and treatment delivery.^14^^,^^15^ Moreover, lesion progression continues for 6–18 months after treatment until disease arrest is achieved.^9^^,^^16^^,^^17^ Allogeneic HSCT is associated with significant morbidity and mortality owing to graft failure, graft-versus-host disease, and immune deficiency, and is not curative for all patients.^8^^,^^12^^,^^18^, ^19^, ^20^ Access to allogeneic HSCT is not universal; disparities in access exist between different racial and ethnic groups in the USA,^14^ and allogeneic HSCT is not routinely available to patients from low or middle income countries.^21^^,^^22^ Moreover, outcomes following allogeneic HSCT may vary by centre experience and the extent of cerebral disease at time of transplant.^8^^,^^12^^,^^20^^,^^23^^,^^24^ Autologous-modified HSCT, although mitigating some of the risks associated with allogeneic HSCT, still requires myeloablative chemotherapy and is not approved outside the USA or for patients without gadolinium-enhancing lesions.^25^^,^^26^ Therefore, there is a significant unmet need for globally accessible, lower-risk therapies that can be administered immediately upon cerebral lesion detection. Development of drug therapies for childhood cerebral adrenoleukodystrophy have so far been unsuccessful.^27^, ^28^, ^29^, ^30^

Leriglitazone is an orally available, neuroprotective, brain-penetrant peroxisome proliferator-activated receptor gamma (PPARγ) agonist that reduces neuroinflammation, promotes myelination and improves mitochondrial function.^31^ In healthy volunteers, leriglitazone demonstrated PPARγ target engagement by decreasing pro-inflammatory biomarkers and increasing adiponectin levels in plasma and cerebrospinal fluid.^32^ A subsequent phase 2/3 trial in adults with adrenomyeloneuropathy showed leriglitazone was well tolerated. Secondary and safety endpoint data indicated that leriglitazone reduced cerebral disease progression.^33^ Radiological changes, defined as the incidence of new gadolinium-enhancing or non-enhancing lesions or the growth of existing non-enhancing lesions, were observed in eight of the 39 patients (21%) receiving placebo versus three of the 77 patients (4%) receiving leriglitazone. Independently, physicians made a clinical diagnosis of progressive cerebral adrenoleukodystrophy in six participants in the placebo group and in no participants receiving leriglitazone. This observed attenuation of lesion development and progression was consistent with biomarker data; markers for axonal degeneration, neuroinflammation, and blood–brain barrier disruption were higher in patients receiving placebo than in those receiving leriglitazone.

Here, we report results from a predefined 24-week interim assessment from a phase 2/3, open-label, multicentre study to assess the safety, study continuation criteria, and effects of leriglitazone on disease progression in boys with cerebral adrenoleukodystrophy.

## Methods

### Study design

NEXUS is a 96-week, open-label, multicentre study for European registration conducted at three sites in Europe and one site in South America between 13 February 2020 and April 2025. The study includes a predefined interim analysis of evaluable patients, conducted at the point that the thirteenth patient to be enrolled reached week 24. At the time of data cut-off for this interim analysis, no participants were enrolled at the South American site. All patients received leriglitazone orally, once-daily, dose-adjusted to achieve target plasma leriglitazone exposure of 170 μg/h/mL (±20%). Dose adjustments could be made based on pharmacokinetic analysis of blood samples obtained during study visits, or at any time during the study for tolerability.

### Ethics

The study was conducted in accordance with the Declaration of Helsinki and all patients’ parents or legal guardians provided written informed consent. All participating sites obtained independent ethics committee or institutional review board approval.

Before providing consent to participate in the study, the parent or legal guardian was informed that the results of the screening evaluations may indicate that the patient is eligible for HSCT and were asked whether HSCT should be undertaken if their child was eligible. They were also asked to provide consent for the patient to receive leriglitazone and attend study visits up until the HSCT procedure was initiated. The informed consent discussion also included the possible scenario of continued treatment with leriglitazone if the patient was found to be ineligible for HSCT after the baseline visit. If HSCT was declined, continued treatment with leriglitazone was offered to the patient as part of the study until the first end-of-treatment criteria occurred.

Participants could undergo HSCT at any point during the study, as determined by the patient's physician and family, without a requirement to fulfil the study HSCT criteria. As such, standard of care treatment was not delayed by participation in the study.

### Patients

The majority of patients were identified through family screening. Boys aged 2–12 years with a diagnosis of X-linked adrenoleukodystrophy with white matter lesions on a magnetic resonance imaging (MRI) scan, consistent with evidence of cerebral disease, were enrolled. Inclusion criteria required that participants had a major functional disabilities (MFD) score of 0 (as determined by key measures in the NFS), a Loes score of 0·5–10·0, with gadolinium-enhancing or non-enhancing lesions at baseline. Participants were included if they had no signs or symptoms of adrenal insufficiency (morning cortisol and aldosterone levels within the normal laboratory range) or were receiving steroid replacement therapy if adrenal insufficiency was present, and had glycated haemoglobin within the normal range. Participants were excluded if they had received pioglitazone or other thiazolidinediones in the 6 months before screening, used greater than 50 mg/day of biotin in the 3 months before screening, had previously undergone HSCT, or had participated in another interventional study in the 6 months before screening. Participants with any other chronic neurological disease diagnosis were excluded.

Participants without lesional gadolinium enhancement at baseline were included in population 1, and those with gadolinium enhancement at baseline were included in population 2.

### Safety endpoints

Adverse events (AEs) and serious adverse events (SAEs) were monitored throughout the study.

### Interim week 24 analysis

The primary endpoint for the NEXUS study is the proportion of patients with clinically and radiologically arrested disease at week 96 or the final visit before HSCT. Here, we present the pre-specified interim analysis of the proportion of patients demonstrating lesion growth deceleration or disease arrest at week 24 (6 months). The threshold for study continuation was a one-sided 95% CI of greater than 10% for the proportion of evaluable patients meeting these continuation criteria. Lesion growth deceleration was defined as growth below that expected from available natural history^34^ and/or a growth rate lower than previous measures. Growth rate was defined as the change in T2/fluid-attenuated inversion recovery (FLAIR)-hyperintensity total lesion volume divided by the number of months elapsed between MRI assessments (cm^3^/month). Acceleration or deceleration were defined as the change in growth rate since previous MRI (cm^3^/month^2^). No significant lesion growth was defined as either a fold change of less than 1·2 since previous MRI (“no growth”) or lesion growth below that expected of natural history.

Arrested disease at week 24 was defined as a change in NFS of less than or equal to 1 from baseline, free of MFDs, and a lack of lesion progression on MRI. A lack of lesion progression was defined as: 1) no conversion to gadolinium-positive lesions in population 1 or disappearance of persistent gadolinium-positive lesions in population 2; and 2) no significant growth (defined above) of T2/FLAIR lesions at week 24 compared with the previous MRI at week 12.

Secondary endpoints were the change from baseline in Loes score, NFS, and gadolinium intensity score (GIS), the overall survival of patients who remained on leriglitazone (ie, have not undergone HSCT), and the number of patients meeting study-specific HSCT criteria.

Study-specific HSCT criteria were defined as the presence of gadolinium-enhancing lesions and/or significant lesion growth across two consecutive MRI scans at least 12 weeks apart. Patients meeting these criteria were recommended for HSCT. Patients could also opt to continue on study treatment as agreed by the patient's family or until a suitable donor was identified. Study-specific HSCT criteria were first assessed at week 24.

Exploratory endpoints included the change from baseline in T2/FLAIR lesion volume and in the concentration of the plasma biomarkers, neurofilament light chain (NfL) and matrix metalloproteinase 9 (MMP-9). NfL and MMP-9 levels were measured using ultrasensitive SIMOA technology (Rules-Based Medicine, Austin, TX, USA) and HumanMAP multiplexed immunoassay panel using the Luminex bead-based instrument platform (Rules-Based Medicine, Austin, TX, USA), respectively.

Clinical assessments of neurological function, NFS and MFD, were conducted at baseline, week 12, and week 24. Cerebral MRI assessments were performed during screening, week 4 (optional), week 12, and week 24. MRI assessments were made on the expert opinion of at least two central readers experienced in neuroimaging of adrenoleukodystrophy. Lesion volumetrics and kinematics were calculated using the 3D Slicer image computing platform^35^ using the T2-weighted volume as the target to calculate lesion volume from the lesion segmentation volume. Data were recorded on a volumetrics rating form (Athinoula A. Martinos Center for Biomedical Imaging, Charlestown, MA, USA; Weill Cornell Medicine, New York, NY, USA; Appendix p 1). Blood samples for plasma biomarkers were taken at baseline, week 4, week 12, and week 24.

### Data analysis

The appropriate sample size was calculated to estimate the proportion of patients meeting arrested disease criteria to distinguish treatment versus the spontaneous arresting rate of 10% in untreated patients.^6^^,^^36^^,^^37^ With 80% power, the initial target enrolment was 13 patients using a criterion of four patients meeting arrested disease with a one-sided significance level of 0·05, and one-sided 95% CI of greater than 10%. Additional patients could be enrolled to ensure that the study recruited, at minimum, 13 evaluable patients.

The safety analysis set included all patients who received at least one dose of leriglitazone. Patients were included in the modified intent-to-treat (mITT) analysis set if they had received at least one dose of leriglitazone and had at least one post-baseline MRI. The pharmacodynamics analysis set included all patients who received at least one dose of leriglitazone and had at least one post-baseline measurable biomarker concentration available in plasma or cerebral spinal fluid. Patients were evaluable for the interim analysis if they had completed the week 24 study visit at the cut-off date.

CIs were calculated using the Clopper–Pearson exact CI. Comparisons to natural history data were performed using data generated by Mallack et al.^34^ In brief, lesion volume was predicted to increase from baseline by a factor of 2·49 per month (95% CI 2·10–2·89). For lesion acceleration, every month the lesion growth velocity increased by 0·10 cm^3^/month (95% CI 0·05–0·14). Natural history data were plotted to follow a linear increase for a follow-up period of 9–12 months.

Data analyses were performed using SAS System Version 9·4 (SAS, Cary, NC, USA).

The trial is registered with ClinicalTrials.gov, NCT04528706.

### Role of the funding source

The sponsor was involved in study design, data collection, data analysis, data interpretation, and drafting and review of the manuscript.

## Results

**Fig. 1:**
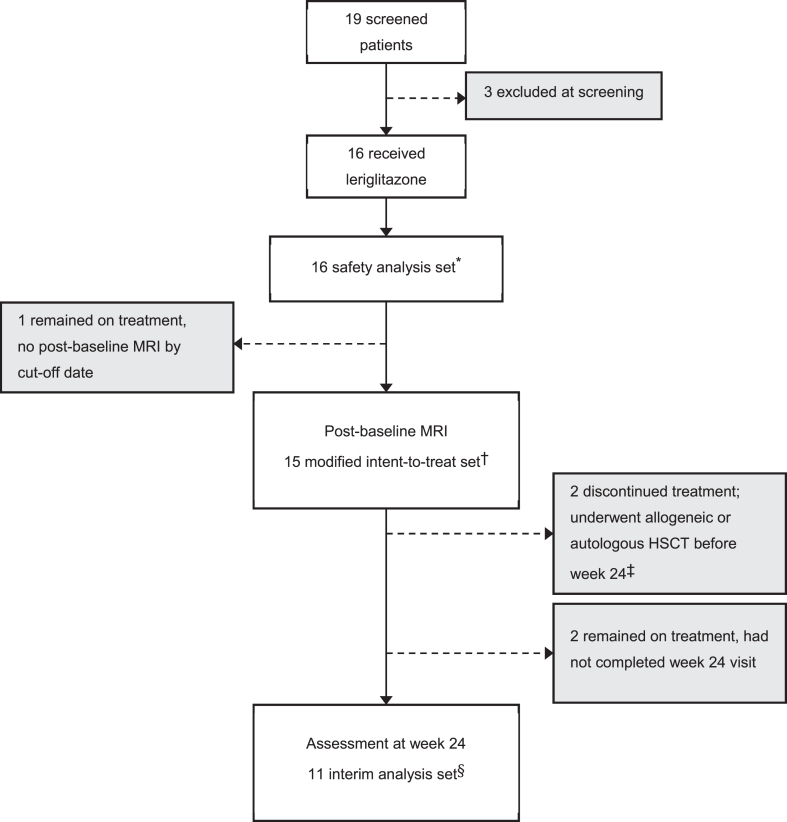
**Disposition of patients**. HSCT, haematopoietic stem-cell transplantation; MRI, magnetic resonance imaging. ∗Patients who received at least one dose of leriglitazone. ^†^Patients who received at least one dose of leriglitazone and had at least one post-baseline MRI. ^‡^Both patients underwent allogeneic HSCT or autologous-modified HSCT at the physician's discretion, study HSCT criteria were not met. ^§^Patients who completed the week 24 study visit at the cut-off date.

At data cut-off, 16 patients had received at least one dose of leriglitazone and were included in the safety analysis set (Fig. 1). Fifteen patients had at least one post-baseline MRI assessment and were included in the mITT analysis set. Individual patient summaries for all patients in the mITT analysis set are presented in the Appendix (p 2). Two patients underwent allogeneic HSCT or autologous-modified HSCT at their physician's or family's discretion before reaching week 24; neither of these patients fulfilled the study-specific HSCT criteria. Eleven patients had completed the week 24 study visit with data available for analysis and thus were considered evaluable and included in the interim analysis set (IAS; Fig. 1).

The median age of patients at screening was 8·5 years (range 4–12 years) and most patients were White (81%). At baseline, six patients were assigned to population 1 (lesions without gadolinium enhancement) and nine patients were assigned to population 2 (lesions with gadolinium enhancement) with a median GIS at baseline of 1·0 (range, 1·0–3·0) (Table 1). Patients in populations 1 and 2 had similar baseline Loes scores (median [range] 1·8 [1·0–3·0] versus 2·0 [1·0–3·0]).

**Table 1:** Patient characteristics at baseline.

	Population 1 (n = 6)	Population 2 (n = 9)	Overall (n = 15)
**Loes score**			
Median (range)	1·8 (1·0–3·0)	2·0 (1·0–3·0)	2·0 (1·0–3·0)
**GIS**			
Median (range)	0·0 (0·0–0·0)	1·0 (1·0–3·0)	1·0 (1·0–3·0)
**NFS (all items)**			
Median (range)	0·0 (0·0–1·0)	0·0 (0·0–0·0)	0·0 (0·0–1·0)
**Lesion volume, cm^3^**			
Median (range)	0·8 (0·2–8·3)	1·6 (0·2–20·2)	1·1 (0·2–20·2)

Data are presented from patients in the modified intent-to-treat analysis set (n = 15). Population 1 are participants without lesional gadolinium enhancement at baseline. Population 2 are participants with gadolinium enhancement at baseline. GIS, gadolinium intensity score; NFS, neurologic function score.

There were 87 AEs in 14/16 patients (88%) in the safety analysis set. Most AEs (84 [96·6%]) were mild and seven (8·0%) were considered related to treatment (Table 2). No patient experienced a treatment-related SAE, and no patients withdrew from the study due to an AE.

**Table 2:** Summary of adverse events.

	Patients	Events, n
AEs	14 (88%)	87
Relationship to study treatment		
Related	4 (25%)	7
Not related	10 (63%)	80
Severity		
Mild	11 (69%)	84
Moderate	3 (19%)	3
Severe	0	0
Life-threatening	0	0
Death	0	0
TEAE leading to study withdrawal	0	0
SAEs	1 (6%)^a^	1

Data are presented from patients in the safety analysis set (n = 16). The most frequent AEs were nasopharyngitis (number of patients, n = 6 [38%]), headache (n = 6 [38%]), severe acute respiratory syndrome coronavirus 2 infection (n = 3 [19%]), oropharyngeal pain (n = 3 [19%]), and eyelid oedema, upper abdominal pain, fatigue, and cough (each n = 2 [13%]). There were no cardiovascular or hepatic AEs. AE, adverse event; SAE, serious adverse event; TEAE, treatment-emergent adverse event.

a Appendicitis leading to hospitalisation.

Continuation criteria were met for all 11 evaluable patients (95% CI 71·5–100·0).

Overall, all 11 patients demonstrated lesion growth deceleration at week 24. In ten patients (91%), lesion acceleration was either absent or below the expected natural history of untreated patients and all patients had no significant lesion growth (Table 3).

**Table 3:** Criteria for arrested disease and lesion deceleration at week 24.

	Population 1 (n = 5)	Population 2 (n = 6)	Overall (n = 11)
**Lesion deceleration**			
**Overall lesion growth deceleration**			
Yes	5 (100%)	6 (100%)	11 (100%)
No	0	0	0
95% CI	47·8–100·0	54·1–100·0	71·5–100·0
**Is there acceleration of lesion growth compared with natural history?**			
Yes	1 (20%)	0	1 (9%)
No, growing with acceleration below natural history	0	1 (17%)	1 (9%)
No, growing at same velocity	0	1 (17%)	1 (9%)
No, growing but decelerating	1 (20%)	2 (33%)	3 (27%)
No, stable lesion	3 (60%)	2 (33%)	5 (45%)
**Is there significant growth?**			
Yes	0	0	0
No, lesion growth below natural history	2 (40%)	4 (67%)	6 (55%)
No, no lesion growth	3 (60%)	2 (33%)	5 (45%)
**Arrested disease**			
**Overall arrested disease**			
Yes	4 (80%)	1 (17%)	5 (45%)
No	1 (20%)	5 (83%)	6 (55%)
95% CI	28·4–99·5	0·4–64·1	16·7–76·6
**Clinical criteria: change in NFS ≤1**			
Yes	5 (100%)	6 (100%)	11 (100%)
No	0	0	0
95% CI	47·8–100·0	54·1–100·0	71·5–100·0
**Clinical criteria: free of MFDs**			
Yes	5 (100%)	6 (100%)	11 (100%)
No	0	0	0
95% CI	47·8–100·9	54·1–100·0	71·5–100·0
**Radiological criteria: lack of lesion progression on MRI**			
Yes	4 (80%)	1 (17%)	5 (45%)
No	1 (20%)	5 (83%)	6 (55%)
95% CI	0·5–71·6	35·9–99·6	23·4–83·3
**Gadolinium enhancement**			
Yes	1 (20%)	5 (83%)	6 (55%)
No	4 (80%)	1 (17%)	5 (45%)
95% CI	28·4–99·5	0·4–64·1	16·7–76·7
**No significant lesion growth**			
Yes	5 (100%)	6 (100%)	11 (100%)
No	0	0	0
95% CI	47·8–100·0	54·1–100·0	71·5–100·0

Data are presented from patients in the interim analysis set (n = 11). CIs constructed using the Clopper–Pearson Exact CI. CIs have been constructed around ‘yes’, with the exception of ‘gadolinium enhancement’ where CI is constructed around ‘no’. Population 1 are participants without lesional gadolinium enhancement at baseline. Population 2 are participants with gadolinium enhancement at baseline. MFD, major functional disability; MRI, magnetic resonance imaging; NFS, neurologic function score.

All 11 evaluable patients met the clinical criteria for arrested disease. For the radiological criteria, five patients (45%) showed a lack of lesion progression (95% CI 23·4–83·3). For the six patients with lesion progression, five were associated with persistence of gadolinium enhancement in population 2, and one patient from population 1 developed lesional gadolinium enhancement. None of these six patients were considered to have significant lesion growth at week 24. Overall, five patients (45%) met both the interim clinical and radiological criteria for disease arrest (95% CI 16·7–76·6) (Table 3).

**Fig. 2:**
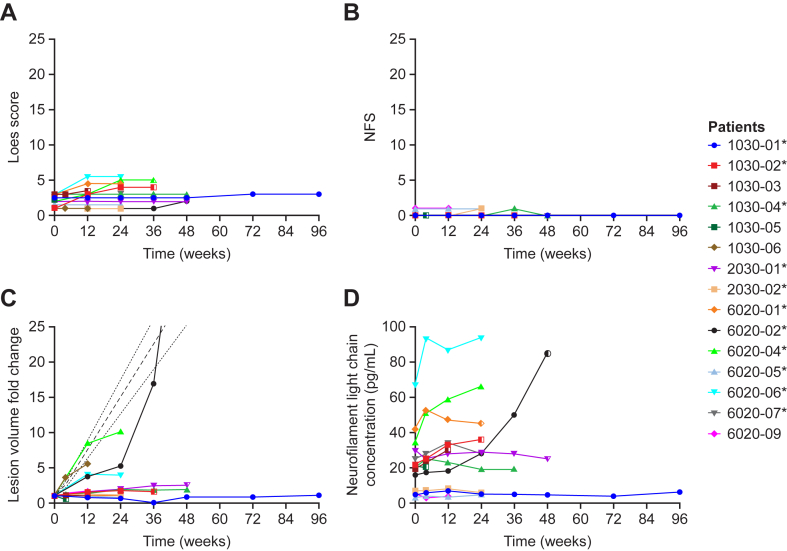
**Radiological and clinical outcomes, lesion volumetric data, and biomarker data in patients receiving leriglitazone**. (A) Loes score. (B) NFS. (C) Total lesion volume fold change. (D) Neurofilament light chain plasma concentration in patients receiving leriglitazone. Data are presented from patients in the modified intent-to-treat analysis set (n = 15). Dashed lines represent the mean and 95% CIs from natural history data.^34^ Data in (B) are plotted on a 0–25 scale to represent the range of the NFS assessment. ∗Patients included in interim analysis set. NFS, neurologic function score.

The overall change in Loes score from baseline to week 24 for population 2 was higher than for population 1 (median change [range] 0·8 [0·0–3·0] versus 0·0 [0·0–3·0]; Appendix p 5). Individual Loes score trajectories for all patients in the mITT analysis set are shown in Fig. 2A. With the exception of one patient, Loes score increased between baseline and week 12. By week 24, the change in Loes score from baseline had stabilised in most patients.

At week 24, all patients remained clinically stable according to the change in NFS from baseline (median change [range] 0·0 [0·0–1·0]; Fig. 2B; Appendix p 5). There was no change in the overall median GIS from baseline (median change [range] 0·0 [−1·0–1·0]; Appendix p 5). Gadolinium enhancement had resolved in one patient in population 2 and developed in one patient in population 1 (Appendix p 6).

The ten evaluable patients who remained on leriglitazone treatment past week 24 were alive at the data cut-off (overall survival, 100%; 95% CI 69·2–100·0).

Five patients (45%) in the IAS met the study-specific HSCT criteria at week 24 owing to persistent gadolinium enhancement, but without significant lesion growth.

At week 24, lesion volume remained stable for patients in population 1, with a median change from baseline of 0·01 cm^3^ (range −1·9–2·2). Patients in population 2 had a greater change in lesion volume with a median change from baseline of 2·68 cm^3^ (range 0·1–16·5). All patients trended towards volume stabilisation, at or below natural history, by week 24.

Lesion volume fold change from baseline was greater in population 2 than population 1 (median change [range] 3·0 [1·0–10·0] versus 1·1 [0·6–1·9]). In the mITT analysis set, all patients exhibited a total and monthly lesion volume fold change below that of untreated patients at week 24 (Fig. 2C; Appendix p 7).

**Fig. 3:**
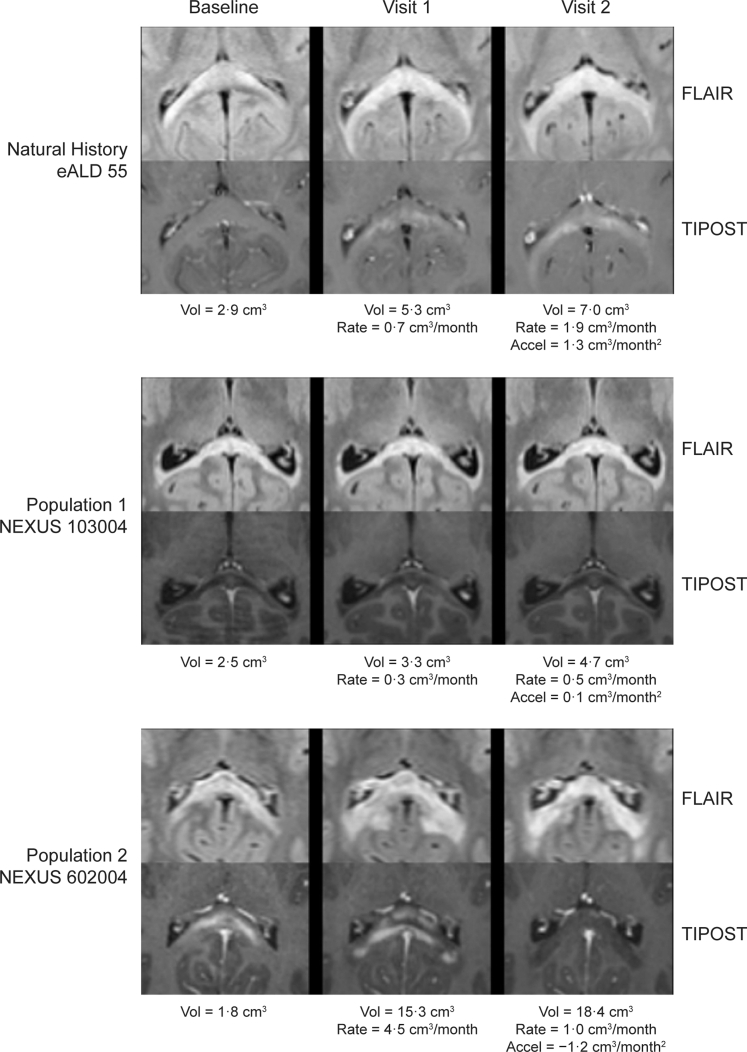
**T2 FLAIR and T1-post contrast MRI scans from an untreated patient from a natural history cohort, and patients in populations 1 and 2 of the NEXUS study**. Rows 1 and 2: early-stage lesion progression in an untreated 4-year-old patient at baseline, visit 1 (3·5 months) and visit 2 (4·4 months), demonstrating the development of lesional enhancement and lesion growth acceleration. Rows 3 and 4: lesion progression in an 8-year-old participant from Population 1 at baseline, visit 1 (3·0 months) and visit 2 (6·0 months), demonstrating a lack of lesional enhancement and slow lesion growth compared to natural history. Rows 5 and 6: lesion progression in a 6-year-old participant from Population 2 at baseline, visit 1 (3·0 months) and visit 2 (6·0 months), demonstrating resolving lesional enhancement and significant lesion growth deceleration inconsistent with the natural history of early-stage childhood cerebral adrenoleukodystrophy. Accel, acceleration; eALD, early-stage adrenoleukodystrophy; FLAIR, fluid-attenuated inversion recovery; MRI, magnetic resonance imaging; T1POST, T1-post contrast; Vol, volume.

The median lesion volume growth velocities for population 1 and population 2 were −0·01 cm^3^/month (range −0·5–0·5) and 0·14 cm^3^/month (range, −0·2–1·0), respectively. Lesion growth decelerated in both populations by week 24, below that expected from natural history (median [range]: population 1, −0·01 cm^3^/month^2^ [−0·1–0·1]; population 2, −0·02 cm^3^/month^2^ [−1·6–0·0]; Appendix p 7). Illustrative examples of MRI scans for an untreated patient and patients from populations 1 and 2 are shown in Fig. 3.

NfL levels were higher at baseline in population 2 than in population 1 (mean [SD], 30·44 pg/mL [16·01 pg/mL] versus 10·20 pg/mL [8·48 pg/mL]). In the pharmacodynamics analysis set, plasma NfL levels were stable between weeks 12 and 24 for most patients (Fig. 2D). Changes in NfL levels from baseline correlated with changes in lesion volume (r = 0·41, p = 0·01; Appendix p 8). For MMP-9, mean (SD) levels at baseline were 185·17 ng/mL (72·07 ng/mL) and 221·00 ng/mL (152·75 ng/mL) for populations 1 and 2, respectively. At week 24, mean MMP-9 levels decreased compared with baseline for both populations (mean change [SD], −78·00 ng/mL [77·32 ng/mL] and −120·08 ng/mL [163·94 ng/mL] for populations 1 and 2, respectively; Appendix p 9).

At the time of the interim analysis, data from beyond week 24 were available for some patients in the mITT analysis set (Fig. 2). One patient showed radiological evidence of disease progression after week 24 (Fig. 2C and Appendix p 2).

## Discussion

In this interim analysis of a phase 2/3 trial on the effects of leriglitazone on disease progression in paediatric patients with early-stage cerebral adrenoleukodystrophy, all evaluable patients met the continuation criteria at week 24. This exceeded the pre-specified study continuation criteria of a one-sided 95% CI of greater than 10%. All patients exhibited lesion growth deceleration without clinical progression; five of the patients also met the criteria for arrested disease. All patients were clinically stable and remained free of MFDs as of February 2024.

Safety data indicate that leriglitazone is well tolerated. Indeed, the safety profile in this paediatric population revealed a lower incidence of oedema and weight gain compared with adult patients with adrenomyeloneuropathy receiving leriglitazone.^33^ At week 24, no treatment-related SAEs had occurred.

The primary aim of this interim analysis was to assess patient safety and determine trial continuation. However, these interim results indicate early changes to the natural history of disease progression. Untreated, the presence or development of blood–brain barrier disruption, as determined by gadolinium contrast enhancement on T1-weighted images, drives the progression of cerebral demyelination, assessed by T2-hypertensity lesion burden (Loes score), and subsequent neurological deterioration characteristic of progressive cerebral adrenoleukodystrophy.^38^^,^^39^ Here, six patients exhibited T1-enhancing lesions uncoupled from the expected increase in Loes score on MRI scan. Loes score data trended towards stabilisation and were comparable with those seen in patients following allogeneic HSCT^2^ or autologous-modified HSCT over 6 months.^9^ Upon volumetric analysis, a more sensitive method of measuring lesion progression in early-stage cerebral adrenoleukodystrophy than Loes score, all patients had lesion deceleration, in contrast to the accelerated growth expected from natural history.^34^ The pattern of deceleration is illustrated in the representative MRI images comparing treated patients from NEXUS with an untreated patient.

This trend towards lesion stabilisation was also demonstrated by changes in NfL and MMP-9 levels, for which elevated plasma concentrations are considered biomarkers of axonal damage and blood–brain barrier disruption, respectively.^40^, ^41^, ^42^, ^43^ Plasma NfL levels have been reported to markedly increase in children with cerebral adrenoleukodystrophy compared to healthy children (median 158·8; [interquartile range 25·3–545·1] pg/mL versus 4·8 [3·6–6·3] pg/mL).^42^ In the present study, plasma NfL levels at baseline (median 20·0 [interquartile range 6·8–30·0] pg/mL) were lower than those previously reported; this difference may be explained by the different disease characteristics of the cohorts. For instance, the NEXUS study includes patients with early disease and limited lesion size at baseline, with a subset of these patients also having non-enhancing lesions. At the 24-week time point, NfL levels in all patients (median 28·0 [interquartile range 5·8–45·0] pg/mL) were maintained below those reported in childhood cerebral adrenoleukodystrophy. These results showed a parallel trajectory with lesion volume, indicated by the correlation between change from baseline in NfL concentration and lesion volume, and most patients showed stabilisation of NfL levels upon treatment with leriglitazone. Plasma MMP-9 levels are reported to increase in boys with cerebral adrenoleukodystrophy compared to healthy controls (mean 71·2 ng/mL versus 184·8 ng/mL).^40^ At week 24, for most patients, MMP-9 levels decreased or stabilised. Overall, radiological and plasma biomarker analyses revealed parallel trends consistent with disease attenuation.

The following context and limitations should be considered. First, where data beyond 24 weeks were available, Loes score, NFS, lesion volume fold change, and plasma biomarkers generally remained stable for most patients. However, at the time of reporting, one patient showed evidence of disease progression beyond week 24 in line with natural history (Fig. 2C). Second, for this 24-week interim analysis, boys with very early-stage disease may have lesions that initially undergo lower rates of growth before the period of exponential expansion. Therefore, they may initially qualify for a diagnosis of arrested disease as defined in the NEXUS study, before the disease fully declares itself. Continued surveillance for the full 96 weeks of the study, beyond the expected phase of rapid lesion growth, is required to accurately classify these patients. Similarly, the median time for gadolinium enhancement to develop in patients without lesional inflammation at diagnosis is 6 months.^15^ For population 1, who had lesions without gadolinium enhancement at baseline, while the interim analysis may capture the beginning of natural history, further changes may be seen at later time points or in a larger sample of patients. Third, the majority of natural history data available for the comparisons made in this study are from populations in the USA, whereas NEXUS enrolled patients at sites in Europe. Differences in access to standard of care may impact disease identification, progression, and presentation between these populations. Fourth, although the eligibility criteria included patients with a Loes score of up to 10·0, no patients with a Loes score greater than 3·0 were enrolled. Therefore, this cohort includes only patients with a limited lesion size at baseline. Fifth, prior to the publication of Mallack et al.,^34^ a subset of MRIs in this dataset were segmented using a supervised semi-automated algorithm in 3D slicer. All segmentations thereafter were completed manually according to the published methods to ensure a direct comparison to natural history. Iterative optimization of the volumetric analysis will continue through the final week 96 analysis. Finally, while the short-term efficacy signals outlined above are encouraging, full follow-up to 96 weeks will be necessary to fully assess the effect of leriglitazone on childhood cerebral adrenoleukodystrophy.

In conclusion, in this interim analysis of a phase 2/3 study of leriglitazone in paediatric patients with cerebral adrenoleukodystrophy, there were no major safety concerns and all patients met the study continuation criteria at week 24. Early clinical, radiological, and plasma biomarker data indicate stable or limited progression of disease over this 6-month follow-up. Given that larger lesions and higher Loes scores are associated with worse outcomes following standard of care treatments,^2^^,^^17^^,^^37^ and many patients do not have timely access to HSCT, leriglitazone may offer clinically meaningful benefit to patients with progressive cerebral adrenoleukodystrophy. Full study follow-up is required to determine whether these effects are sustained; the NEXUS study is ongoing to 96 weeks of treatment, with expected completion in April 2025.

## Contributors

All authors contributed to the drafting of this manuscript and approved the final submission. All authors had full access to all the data in the study and had final responsibility for the decision to submit for publication. SB and PLM had full access to and verified the data reported.

## Data sharing statement

The study sponsor, Minoryx Therapeutics SL, is committed to sharing access to patient-level data and supporting clinical documents with qualified external researchers. Individual anonymised participant data and relevant clinical study documents will first be made available between 12 and 24 months after publication of the final, week 96, study results. Data requests will be reviewed and approved by an independent review panel on the basis of scientific merit. All data will be anonymised, to respect the privacy of patients who have participated in the trial in line with applicable laws and regulations. A data-sharing agreement signed by the requesting researcher(s) is required before data access can be provided. Proposals should be submitted to SP (spascual@minoryx.com).

## Declaration of interests

AGC has received honoraria for research support and lectures from Immedica and PTC Therapeutics, honoraria for lectures from BioMarin and Recordati Rare Diseases Foundation, and is a co-founder of Neuroprotect Life Sciences.

CS has received research grants, conference support and consulting fees from BioMarin, Bluebird Bio, Forge Biologics, Minoryx Therapeutics, Shire and Takeda, and served as principal or co-principal investigator in clinical trials for Bluebird Bio, Ionis, Minoryx, and Takeda.

JR, EY, SJ, OR, PC and DB have no disclosures.

HR has received institutional research support from the German Research Council and the Eva Luise and Horst Köhler Foundation and has received research support/grants and travel support from Minoryx Therapeutics SL.

GCC has been compensated as consultant for Life Molecular Imaging and as an invited speaker for CME with Efficient CME and Horizons CME, and has received institutional research support from the National Institutes of Health and National Institute on Aging (R01AG068398, R01AG080011).

KGH has received institutional research support from Minoryx Therapeutics SL and the National Institutes of Health.

SB is an employee of CTI Clinical Trial and Consulting Services, Inc.

ME and PP are employees of Minoryx Therapeutics SL.

MR, LRP, GP, AV, A Mistry, and SP are employees of, and have stock in, Minoryx Therapeutics SL.

MP and A Mantilla are former employees of Minoryx Therapeutics SL.

UM is a former employee and stockholder of, and serves as a consultant for, Minoryx Therapeutics SL.

MM is an employee of, serves on the board of directors of, and has stock in, Minoryx Therapeutics SL, and has received intellectual property interests from a discovery or technology relating to healthcare.

PLM has received consulting fees from INC Pharma, Inozyme, IONIS Pharmaceuticals, Biogen, Bluebird Bio, Minoryx Therapeutics SL, and Vertex Pharmaceuticals Inc, and served a leadership or fiduciary role for ALD connect, Young Genetic Stroke Alliance, and the United Leukodystrophy Foundation.

EM has received support from the National Institutes of Health, National Institute of Neurological Disorders and Stroke (K12NS066274, K23NS118044), for the development of the quantitative MRI models subsequently adopted by this work and has been compensated for serving on a Scientific Advisory or Data Safety Monitoring Board for Grace Science and Lysogene.

## Appendix A Supplementary data

Supplementary Appendix

Protocol

Signed Final.pdf
