# Supplementary material for: Safety and efficacy of leriglitazone in childhood cerebral adrenoleukodystrophy (NEXUS): an interim analysis of an open-label, phase 2/3 trial
Source: eClinicalMedicine. 2025 May 24;84:103265. doi: 10.1016/j.eclinm.2025.103265 (PMC12159931; doi:10.1016/j.eclinm.2025.103265)
Supplement: Supplementary Appendix [file mmc1.docx]

# Supplementary appendix

## NEXUS volumetrics form

version 1.0, 01 September 2022

Outputs based on volumetrics:

| Volumetrics | |
| --- | --- |
| Lesion volume (cc): |  |
| Normalized lesion volume (cc):  Lesion volume normalized by the total brain volume |  |
| Normalized lesion volume (cc):  Lesion volume normalized by the white matter volume |  |
| Fold change from baseline: Change in (non-normalized) volume |  |
| Fold change from baseline (per month): |  |
| Change in (non-normalized) volume |  |
| Velocity: |  |
| Change in (non-normalized) volume over time from previous visit (12 weeks apart) | |
| Acceleration: |  |
| Change in velocity from previous visit (12 weeks apart) |  |

| Expert assessment of lesion growth* | |
| --- | --- |
| At this timepoint, is there acceleration of lesion growth according to natural history? | - Yes, acceleration according to or above untreated natural history - No (select option below) - N/A (baseline visit or V6) |
| If NO, select: | - growing with acceleration, but below natural history - growing at same velocity (change in velocity -0.01 to +0.01 cc/month) - growing but decelerating (change in velocity < -0.01 cc/month) - stable lesion. No further growth - N/A |
| At this timepoint, is there significant growth (based on radiological data and natural history)? | - Yes, progression according to or above untreated natural history - No (select option below) - N/A (baseline visit) |
| If NO, select: | - lesion growth, but below what would be expected based on natural history - no lesion growth (fold change since previous MRI < 1.2) - N/A |
| At this timepoint, is the patient meeting the HSCT criteria? | - Yes (select option below) - No - N/A (baseline visit or V6) |
| If YES, select: | - Gd+ at two consecutive visits (GIS ≥ 1) - Significant growth at two consecutive visits - Both, Gd+ and significant growth at two consecutive visits - N/A |
| Comments: | |

**Date:**

**Signature:**

*Reference natural history literature and data: Mallack et al. 2021:

- Growth (untreated natural history fold change assessment from baseline = 2.5/month [95%CI 2.1 - 2.9])
- Acceleration (untreated natural history: every month the lesion growth velocity increases by 0.10 cc [95%CI 0.05 - 0.14])

Gd+= gadolinium enhancement. GIS=gadolinium intensity score. MRI=magnetic resonance imaging. N/A=not applicable.

| **Patient ID** | **Population** | **Assessment** | **Loes score** | **GIS** | **NFS** | **MFD** | **Study HSCT criteria met?** | **HSCT performed?** | **Factors influencing HSCT decision** | **Relevant MRI and clinical findings up to the latest recorded visit*** |
| --- | --- | --- | --- | --- | --- | --- | --- | --- | --- | --- |
| 103001 | 1 | Baseline | 2.5 | 0 | 0 | 0 | NA | NA | NA | Stable lesion volume  Met arrested disease criteria at week 24 and week 96 |
|  |  | Week 96 | 3·0 | 0 | 0 | 0 | N | N | NA |  |
| 103002 | 1 | Baseline | 1·0 | 0 | 0 | 0 | NA | NA | NA | Stable lesion volume  No significant T2/FLAIR lesion growth  Did not meet arrested disease criteria at week 24 |
|  |  | Week 39 | 4·0 | 1 | 0 | 0 | Y | Y | Met study HSCT criteria |  |
| 103004 | 1 | Baseline | 3·0 | 0 | 0 | 0 | NA | NA | NA | Lesion volume increased from baseline to week 24 and then stabilised  No significant T2/FLAIR lesion growth  First gadolinium enhancement at week 36  Met arrested disease criteria at week 24 |
|  |  | Week 48 | 3·0 | 1 | 0 | 0 | Y | N | NR |  |
| 203002 | 1 | Baseline | 1·0 | 0 | 0 | 0 | NA | NA | NA | Stable lesion volume  Episodes of incontinence at week 24 (NFS: 1) which subsequently resolved  Met arrested disease criteria at week 24 |
|  |  | Week 24 | 1·0 | 0 | 1 | 0 | N | N | NA |  |
| 602005 | 1 | Baseline | 1.5 | 0 | 1 | 0 | NA | NA | NA | Lesion volume decreased from baseline to week 24  Running difficulties and hyperreflexia (NFS: 1) persisted from baseline to week 24  No significant T2/FLAIR lesion growth  Met arrested disease criteria at week 24 |
|  |  | Week 24 | 1.5 | 0 | 1 | 0 | N | N | NA |  |
| 602009 | 1 | Baseline | 2·0 | 0 | 1 | 0 | NA | NA | NA | Stable lesion volume  Running difficulties and hyperreflexia (NFS: 1) persisted from baseline to week 12  Did not complete week 24 by data cut-off |
|  |  | Week 12 | 2·0 | 0 | 1 | 0 | NA | N | NA |  |
| 103003 | 2 | Baseline | 3·0 | 1 | 0 | 0 | NA | NA | NA | Stable lesion volume  Discontinued before week 24 |
|  |  | Week 12 | 3.5 | 1 | 0 | 0 | N | Y | Investigator and family decision owing to increased Loes score and gadolinium enhancement. |  |
| 103005 | 2 | Baseline | 2·0 | 1 | 0 | 0 | NA | NA | NA | Stable lesion volume  Discontinued before week 24 |
|  |  | Day 35 | 2·0 | 1 | NR | NR | N | Y | Investigator and family decision |  |
| 103006 | 2 | Baseline | 1·0 | 1 | 0 | 0 | NA | NA | NA | Lesion volume increased from baseline to week 12  Significant T2/FLAIR lesion growth at week 4, but not week 12  Did not complete week 24 by data cut-off |
|  |  | Week 12 | 1·0 | 1 | 0 | 0 | NA | N | NA |  |
| 203001 | 2 | Baseline | 2·0 | 1 | 0 | 0 | NA | NA | NA | Lesion volume increased from baseline  Lesions were no longer gadolinium enhancing at week 12  No significant T2/FLAIR lesion growth  Met arrested disease criteria at week 24 |
|  |  | Week 48 | 2·0 | 0 | 0 | 0 | N | Y | Investigator and family decision |  |
| 602001 | 2 | Baseline | 3·0 | 3 | 0 | 0 | NA | NA | NA | Stable lesion volume  Did not meet arrested disease criteria at week 24 |
|  |  | Week 24 | 4.5 | 2 | 0 | 0 | Y | Y | Met study HSCT criteria |  |
| 602002 | 2 | Baseline | 1·0 | 1 | 0 | 0 | NA | NA | NA | Exponential lesion growth after week 24  NfL and MMP-9 concentrations increased after week 24  Did not meet arrested disease criteria at week 24  Lesion growth consistent with natural history |
|  |  | Week 48 | 2·0 | 1 | 0 | 0 | Y | N | Lack of expert centre available outside of Europe.  HSCT was performed at week 50. |  |
| 602004 | 2 | Baseline | 2·0 | 3 | 0 | 0 | NA | NA | NA | Lesion volume increased from baseline to week 12, and growth decelerated at week 24  No significant T2/FLAIR lesion growth  Did not meet arrested disease criteria at week 24 |
|  |  | Week 24 | 5·0 | 3 | 0 | 0 | Y | N | Lack of expert centre available outside of Europe.  HSCT was performed after week 24. |  |
| 602006 | 2 | Baseline | 3·0 | 1 | 0 | 0 | NA | NA | NA | Lesion volume increased from baseline to week 12, and stabilised at week 24  GIS increased to 3 at week 12  Did not meet arrested disease criteria at week 24 |
|  |  | Week 24 | 5.5 | 1 | 0 | 0 | Y | N | Lack of expert centre available outside of Europe.  HSCT was performed after week 24. |  |
| 602007 | 2 | Baseline | 3·0 | 1 | 0 | 0 | NA | NA | NA | Lesion volume increased from baseline with no acceleration  Did not meet arrested disease criteria at week 24 |
|  |  | Week 24 | 3·0 | 1 | 1 | 0 | Y | N | No donor available. Haploidentic transplant under consideration. |  |

## Supplementary Table 1: Individual patient summaries at baseline and most recent* study visit

Population 1 are participants without lesional gadolinium enhancement at baseline. Population 2 are participants with gadolinium enhancement at baseline. FLAIR=fluid-attenuated inversion recovery. GIS=gadolinium intensity score. HSCT=haematopoietic stem-cell transplantation. MFD=major functional disability. MMP-9=matrix metalloproteinase 9. MRI=magnetic resonance imaging. N=no. NA=not applicable. NfL= neurofilament light chain. NFS=neurologic function score. NR=not recorded. Y=yes. *At the time of data cut-off.

|  | **Population 1 (n=5)** | **Population 2 (n=6)** | **Overall (n=11)** |
| --- | --- | --- | --- |
| **Loes score** |  |  |  |
| Median (range) | 0·0 (0·0–3·0) | 0·8 (0·0–3·0) | 0·0 (0·0–3·0) |
| **GIS** |  |  |  |
| Median (range) | 0·0 (0·0–1·0) | 0·0 (−1·0–0·0) | 0·0 (−1·0–1·0) |
| **NFS (all items)** |  |  |  |
| Median (range) | 0·0 (0·0–1·0) | 0·0 (0·0–1·0) | 0·0 (0·0–1·0) |

## Supplementary Table 2: Change from baseline to week 24 for Loes score, GIS, and total NFS

Data are presented from patients in the interim analysis set (n=11). Population 1 are participants without lesional gadolinium enhancement at baseline. Population 2 are participants with gadolinium enhancement at baseline. GIS=gadolinium intensity score. NFS=neurologic function score.


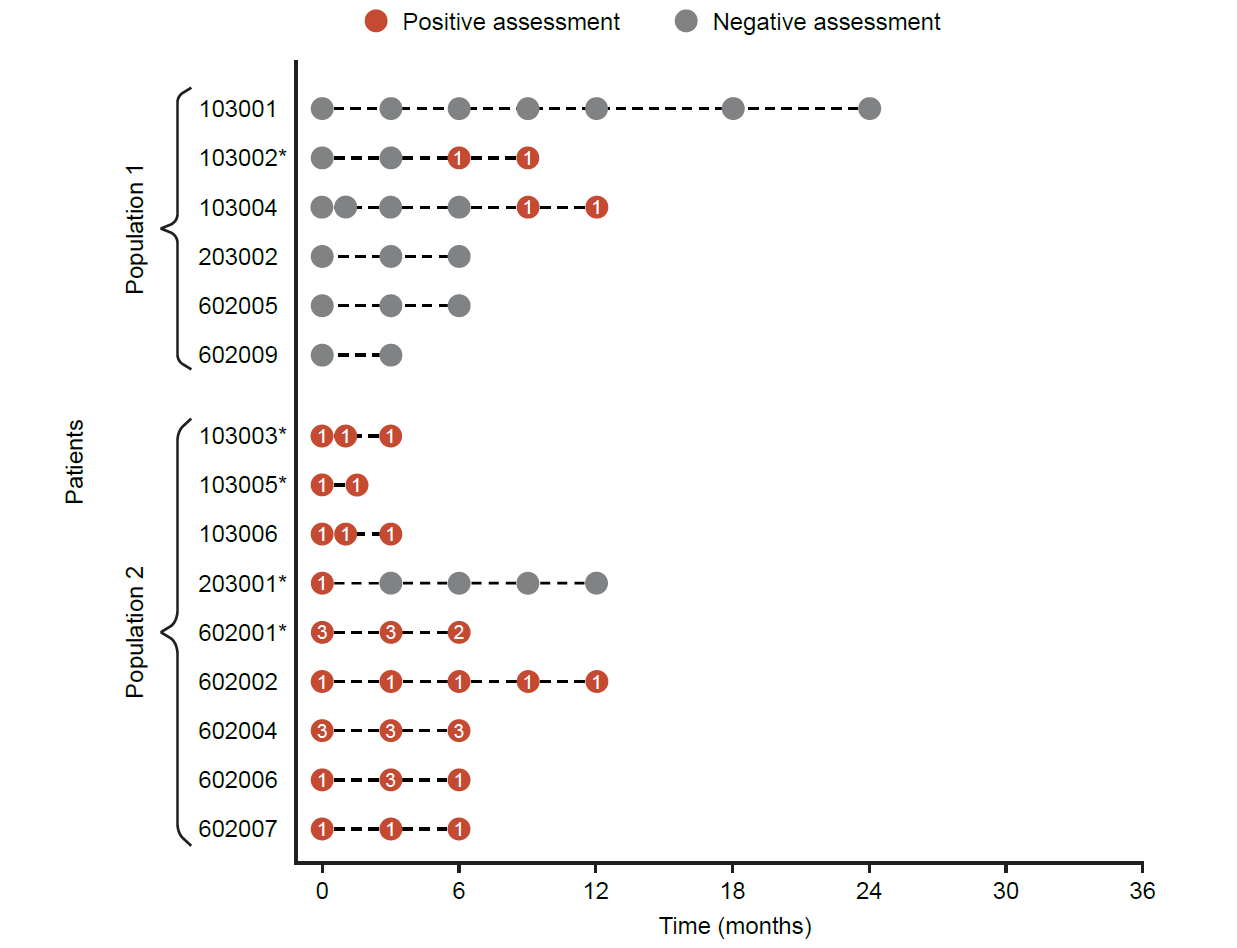


## Supplementary Figure 1: Gadolinium enhancement by visit

Data are presented from patients in the modified intent-to-treat analysis set (n=15). Population 1 are participants without lesional gadolinium enhancement at baseline. Population 2 are participants with gadolinium enhancement at baseline. Numbers within each data point indicate the GIS at each visit. GIS=gadolinium intensity score. HSCT=haematopoietic stem-cell transplantation. *Patients who underwent HSCT.

##
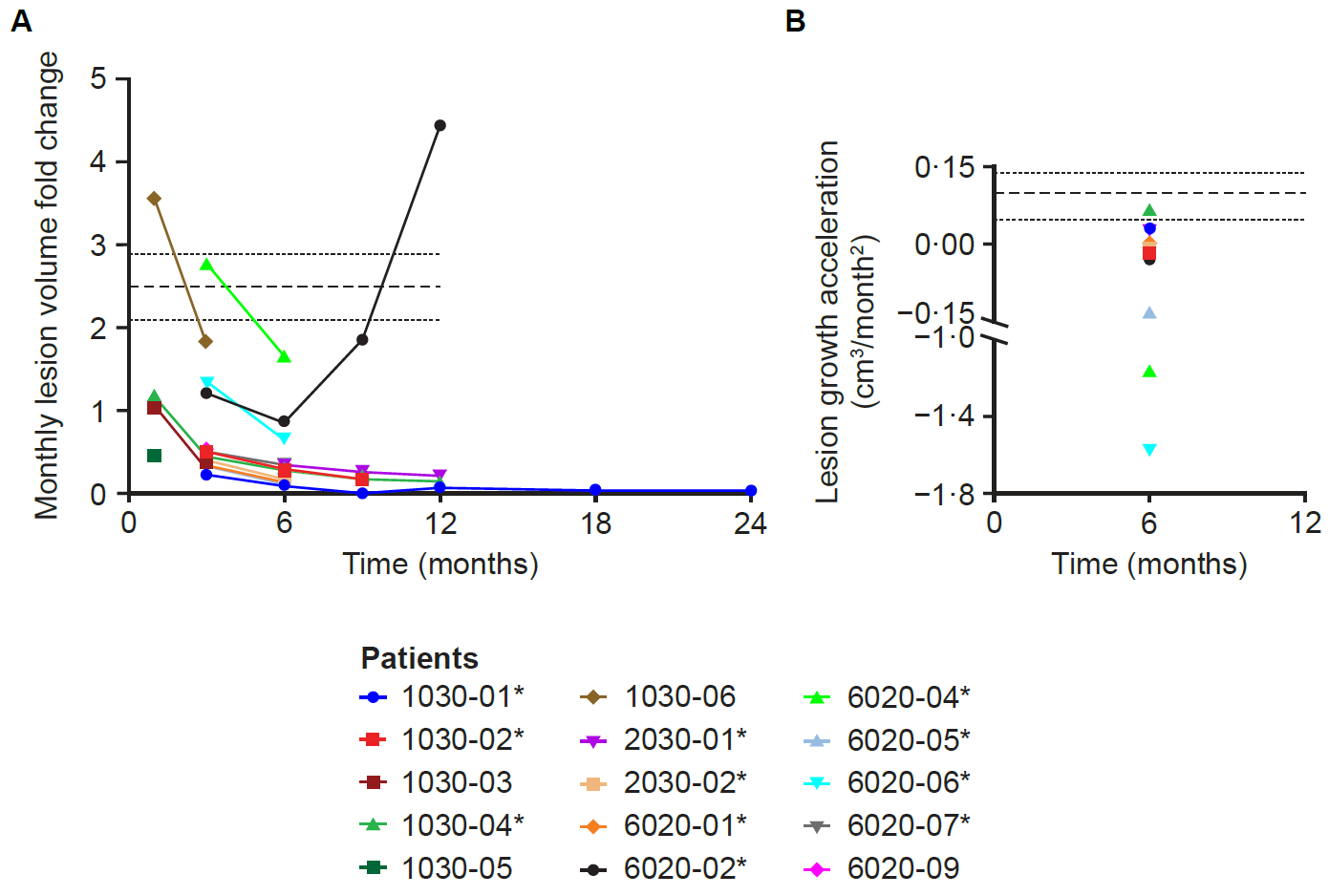
Supplementary Figure 2. Lesion volumetric data

(A) Monthly lesion volume fold change and (B) lesion growth acceleration. Data in (A) are presented from patients in the modified intent-to-treat analysis set (n=15) and from patients in the interim analysis set (n=11) in (B). Dashed lines represent the mean and 95% CIs from natural history data up to 12 months. *Patients included in interim analysis set.

## ***Supplementary Figure 3.* Change from baseline in neurofilament light chain levels and lesion volume**

Data are presented from post-baseline study visits for patients in the pharmacodynamics analysis set (n=15). Correlation was analysed using Spearman’s rank test.


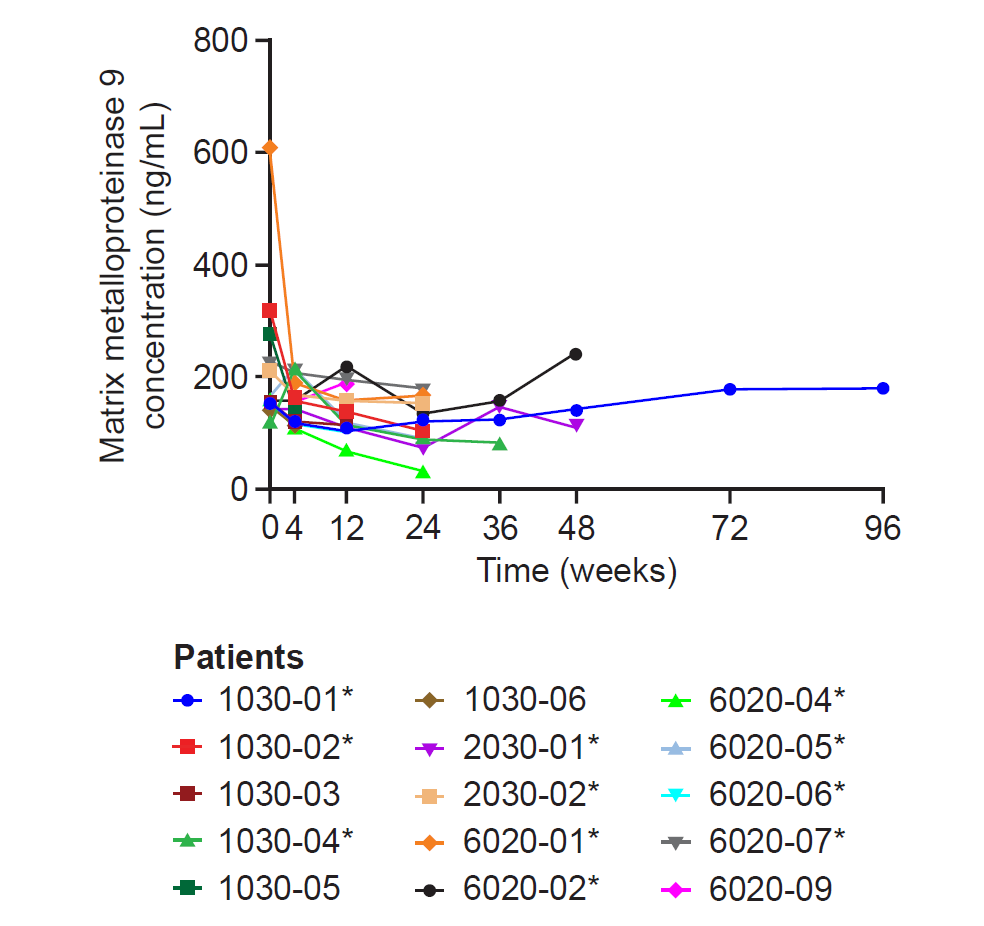


## Supplementary Figure 4. Matrix metalloproteinase 9 levels

Data are presented from patients in the pharmacodynamics analysis set (n=15).

*Patients included in interim analysis set.
