# Supplementary material for: Safety and efficacy of leriglitazone in childhood cerebral adrenoleukodystrophy (NEXUS): an interim analysis of an open-label, phase 2/3 trial
Source: eClinicalMedicine. 2025 May 24;84:103265. doi: 10.1016/j.eclinm.2025.103265 (PMC12159931; doi:10.1016/j.eclinm.2025.103265)
Supplement: Protocol [file mmc2.pdf]

CLINICAL STUDY PROTOCOL

AN OPEN-LABEL, MULTICENTER STUDY IN MALE PEDIATRIC PATIENTS WITH CEREBRAL X-LINKED ADRENOLEUKODYSTROPHY (CALD) TO ASSESS THE EFFECTS OF MIN-102 TREATMENT ON DISEASE PROGRESSION PRIOR TO HUMAN STEM CELL TRANSPLANT (HSCT)

CONFIDENTIAL

Sponsor code: **MT-2-02**
EudraCT number: **2019-000654-59**
EU CT number: **2024-513774-21-00**

Investigational product	MIN-102
Clinical phase	Phase II
Indication to be studied	Adrenoleukodystrophy
SPONSOR:	Minoryx Therapeutics S.L.<br>Av. Ernest Lluch 32, TCM3<br>08302 Mataró (Barcelona)<br>Spain
CONTRACT RESEARCH ORGANIZATION	CTI<br>100 E. RiverCenter Blvd.<br>Covington, KY 41011<br>USA

Version 7.0, 24 April 2024

This study will be conducted in compliance with Good Clinical Practice (GCP), the Declaration of Helsinki (with amendments) and in accordance with local legal and regulatory requirements.

Confidentiality Statement:
This document contains information that is the property of Minoryx S.L., Spain and therefore is provided to you in confidence for review by you, your staff, an applicable institutional review board and regulatory authorities. It is understood that this information will not be disclosed to others without written approval from Minoryx S.L.

AUTHORIZATION OF CLINICAL STUDY PROTOCOL

Sponsor

Minoryx Therapeutics, S.L.
Av. Ernest Lluch 32, TCM3
08302 Mataró (Barcelona)
Spain

Chief Executive Officer (CEO)

[redacted] *Signature:* .... *Date:* ....

Chief Medical Officer (CMO)

[redacted] *Signature:* .... *Date:* ....

INVESTIGATOR PROTOCOL AGREEMENT PAGE

I agree:

- To assume responsibility for the proper conduct of the study at this site.
- To conduct the study in compliance with this protocol, any future amendments, and with any other study conduct procedures provided by Minoryx Therapeutics S.L.
- Not to implement any changes to the protocol without written agreement from Minoryx Therapeutics S.L. and prior review and written approval from the Institutional Review Board (IRB) or Independent Ethics Committee (IEC) except where necessary to eliminate an immediate hazard to patients.
- That I am thoroughly familiar with the appropriate use of the study medication, as described in this protocol and any other information provided by Minoryx Therapeutics S.L. including, but not limited to, the current Investigator's Brochure.
- That I am aware of, and will comply with, Good Clinical Practice (GCP) and all applicable regulatory requirements.
- To ensure that all persons assisting me with the study are adequately informed about the Minoryx Therapeutics S.L. study medication and have been trained on their study-related duties and functions as described in the protocol.

Signature:

Date:

Name (print):

Investigator

Institution name and address (print):

SERIOUS ADVERSE EVENT CONTACT INFORMATION

In case of a serious adverse event (See section 6.1), SAE must be reported by the investigator or designee within 24 hours to:

Contract Research Organization:
Premier Research
3800 Paramount Parkway, Suite 400
Morrisville, North Carolina, USA

SAE Email:
US: PVDS-NA@premier-research.com
ROW: PVDS-ROW@premier-research.com

CONTACT INFORMATION

Role	Company / Address
**Sponsor**	Minoryx Therapeutics S.L.<br>Av. Ernest Lluch 32, TCM3<br>08302 Mataró (Barcelona)<br>Spain
**Sponsor's Main Contact**	[redacted]<br>Minoryx Therapeutics S.L.<br>[redacted]
**Sponsor's Medical Expert**	[redacted]<br>Minoryx Therapeutics S.L.<br>[redacted]
**CRO**<br>**For Regulatory, Monitoring, Site Management, Data Management, Electronic Data Capture systems, Statistics and Clinical Study Report**	CTI Clinical Trial & Consulting Services<br>100 E. RiverCenter Blvd.<br>Covington, KY 41011<br>USA
**Pharmacovigilance**	Premier Research<br>3800 Paramount Parkway, Suite 400<br>Morrisville, North Carolina, USA<br><br>SAE Email:<br>US: PVDS-NA@premier-research.com<br>ROW: PVDS-ROW@premier-research.com
**Centralized MRIs Reading & image collection**	MGH/HST Athinoula A. Martinos<br>Center for Biomedical Imaging<br>149 13th Street, Suite 2301<br>Charlestown, MA 02129<br>USA
**Central Laboratory**	Central Lab for safety<br>**PPD Central Laboratory**<br>929 North Front Street<br>Wilmington, NC 28401<br>USA<br><br>Laboratory for Analysis of Pharmaceutical Product, Plasma, and Cerebrospinal Fluid Samples for MIN-102<br>**ICON**<br>**(former PRA Health Sciences - Early Development Services)**<br>**Bioanalytical Laboratory**<br>Amerikaweg 18<br>9407 TK Assen<br>The Netherlands

Role	Company / Address
	Biomarkers analysis<br><br>**QPS Holdings, LLC**<br>Petrus Campersingel 123|9713 AG Groningen<br>P.O. Box 137, 9700 AC Groningen<br>The Netherlands<br><br>**Rules Based Medicine**<br>3300 Duval Road<br>Austin, TX 78759<br>USA
**Development and application of a dose adjustment algorithm for the use in pediatric patients**	**Calvagone Sarl**<br>772 Montée de Chalier<br>69400 Liergues<br>France
**Patient Travel & Reimbursement**	**MDE Services Group Limited**<br>Building 329, Doncastle Road<br>Bracknell, Berkshire, RG12 8PE<br>United Kingdom
**Drug Supplies Management & Distribution**<br><br>Drug Study Manufacturers, primary packaging and labelling, final configuration and final release of the clinical supplies	Laboratorium Sanitatis SL - TECNALIA<br>P.T. Alava – C/ Leonardo Da Vinci, 11<br>01510 Miñano (Álava)<br>Spain
**Home Nursing**	Illingworth Research Group Limited<br>St. George's House, 1 St George's Street<br>Macclesfield, Cheshire, SK11 6TG.<br>United Kingdom

SYNOPSIS

Protocol Title

AN OPEN-LABEL, MULTICENTER STUDY IN MALE PEDIATRIC PATIENTS WITH CEREBRAL X-LINKED ADRENOLEUKODYSTROPHY (CALD) TO ASSESS THE EFFECTS OF MIN-102 TREATMENT ON DISEASE PROGRESSION PRIOR TO HUMAN STEM CELL TRANSPLANT (HSCT)

Study codes

Sponsor code: MT-2-02
EudraCT number: 2019-000654-59
EU CT number: 2024-513774-21-00

Sponsor

Minoryx Therapeutics S.L., Av. Ernest Lluch 32, TCM3, 08302 Mataró (Barcelona), Spain
Sponsor's Contact: [redacted]

Study Sites

The study will be conducted at approximately 4 sites**.**

Clinical Phase

This is an open-label phase 2 study.

Number of Subjects

It is anticipated that approximately 13 subjects with early lesions will be included in the study.

Primary Objective and Outcomes

To evaluate whether MIN-102 can arrest disease progression of cALD at week 96, as determined by serial clinical and magnetic resonance imaging (MRI) investigations in pediatric subjects.

"Arrested disease" will be assessed at two timepoints:

- 24 weeks after start of treatment (Visit 8), and
- 96 weeks after start of treatment (Visit 12)

The first assessment will be conducted when up to 13 evaluable patients have reached 24 weeks of treatment. The second timepoint represents the confirmatory timepoint.

"Arrested disease" is defined using the following parameters:

- Change in Neurological Function Score (NFS) from Baseline ≤1 point (Week 24) or ≤5 points (Week 96)
- Free of MFD
- Lack of lesion progression on MRI, fulfilling the following:
    - No conversion to Gd+ lesions (Population 1) defined as GIS score of 0.
    - Disappearance of persistent Gd+ lesions (Population 2) defined as change in GIS score from 1, 2 or 3 to 0. Persistent Gd+ lesions is defined as those present in ≥ 2 consecutive MRIs spanning a minimum of 6 months.
    - No significant growth of T2/FLAIR lesions compared to the previous MRI (both Populations) as assessed by central reading.

The study will be continued if a minimum of 4 of 13 evaluable patients have met the endpoint of "arrested disease" at Week 24 or if the overall assessment from central reading suggests lesion growth deceleration ("continuation criteria"). Lesion deceleration shall be considered when lesion growth determined by central readers assessment is below what would be expected based on the literature (Liberato et al., 2019, Mallack et al., 2019, Mallack et al., 2020, Mallack et al., 2021) and/or when lesion growth rate of the latest measure is lower than previous ones. Growth rate is defined as the increase in T2/FLAIR lesion volume since previous MRI divided by the number of months elapsed since that previous MRI.

The study will be considered successful if a minimum of 4 patients meet all "arrested disease" criteria at Week 96, except for MFD where >70% of all evaluable patients at Week 96 have to be free of MFD.

Secondary Objectives and Outcomes

To determine the effects of MIN-102 treatment on further clinical and imaging parameters.

Secondary efficacy endpoints at 24 weeks, 96 weeks, or immediately prior to HSCT

- Sustained change from Baseline in the score composed of NFS items 1 (hearing/auditory processing), 2 (aphasia/apraxia), 4 (vision impairment), 10 (spastic gait) and 13 (incontinence)
- Sustained change from Baseline in total NFS score
  "Sustained change" is defined as the same total score for NFS items 1, 2, 4, 10, and 13 and no change >1 in NFS total score observed in two the consecutive Visits 6–8, and Visits 11-12, respectively. If "sustained change" definition is not met, the average of the two scores of Visit 6 and 8, and Visit 11 and 12, respectively, will be used. If NFS total score differs by 1 point between V6-8, or V11-12, the higher of the two scores is used.
- Change from baseline in Loes MRI severity score
- Change from baseline in Gadolinium Intensity Score (GIS)
- Overall survival of patients who have not undergone HSCT
- Number of patients meeting HSCT criteria

Exploratory Objectives and Outcomes

To determine the effects of MIN-102 treatment on further imaging and on biochemical parameters at 24 weeks, 96 weeks, or immediately prior to HSCT.

- Change from baseline in white matter T2/FLAIR hyperintensity lesion volume and fiber structure as determined by diffusion tensor imaging (DTI)
- Change from baseline in cerebral blood flow, blood volume, capillary mean transient time heterogeneity (CHT) and constants of permeability (K2 and Kapp) as determined by dynamic susceptibility contrast (DSC) MRI
- Changes in the levels of each biomarker in plasma and CSF (optional) from baseline

Pharmacokinetic Endpoints

- Area under the time-concentration curve (AUC), maximum plasma concentration (Cmax), minimum plasma concentration (Cmin).

Safety and Tolerability Endpoints

- Adverse events (AEs)
- Serious adverse events (SAEs) and suspected unexpected serious adverse reactions (SUSARs)
- Vital signs (body weight, height, blood pressure, pulse rate, and body temperature)
- Physical examination
- 12-lead electrocardiogram (heart rate and the following intervals: PR, RR, QRS, QT, QT corrected for heart rate using Fridericia's formula [QTcF])
- Echocardiogram (for all subjects developing peripheral edema)
- Clinical laboratory test results
- Palatability of study drug

Study Medication

Study drug

**MIN-102**	:5[redacted]
**INN**	**:** leriglitazone
Activity	: Peroxisome proliferator-activated receptor gamma (PPARγ) agonist
Strength	: [redacted]
Dosage form	: Oral suspension
Manufacturer	: Laboratorium Sanitatis SL<br>P.T. Alava – C/ Leonardo Da Vinci, 11<br>01510 Miñano (Álava) – Spain<br>Tel.: +34 902 760 000

The study drug is supplied as an oral aqueous-based suspension and is administered orally once daily (1 mL of oral suspension contains 13.66 mg of leriglitazone, 15 mg of leriglitazone hydrochloride). Company code MIN-102 is used to refer to the salt of leriglitazone (as hydrochloride) when administered, however, when reporting plasma or cerebrospinal fluid (CSF) concentrations/exposure MIN-102 refers to the free base (leriglitazone).

Subjects will take 1 dose of MIN-102 at approximately the same time each morning, administered with a 5-mL syringe. The starting dose will be established per body weight in each subject, as determined by PBPK modeling, to reach an exposure of approximately 170 µg•hr/mL(target exposure). Subsequent dose adjustments to achieve the target exposure may be made at any time during the study if deemed necessary, based on the analysis of PK samples obtained at all on-site visits.

Duration of Treatment and End-of-Treatment Criteria

Subjects will receive treatment until the first occurrence of any of the following end-of-treatment criteria:

1. Subject undergoes HSCT, having met the following criteria ("HSCT criteria"):
    - GIS of 1, 2 or 3, or
    - Significant T2/FLAIR lesion growth assessed by central reading

   These criteria must be present at two consecutive MRIs at least 12 weeks apart from each other.

2. Subject undergoes HSCT, if investigators' overall assessment of lesion progression mandates immediate scheduling of HSCT although "HSCT criteria" are not met.

   Subjects who undergo HSCT according to criteria 1 and 2 above may receive study drug until the day before the last study visit prior to initiating HSCT procedures ("Visit Prior to HSCT"). This visit will occur a minimum of 5 days before first administration of myeloablative medication.

3. Study fails to meet "continuation criteria" at week 24
4. Subject fails to meet primary endpoint of "arrested disease" at Week 96.
5. The confirmatory evaluation of the study is performed at Week 96 (Visit 12) and shows that less than 4 of 13 enrolled patients meet criteria for "arrested disease".
6. The subject's parent/legal guardian withdraws consent.
7. In the clinical judgment of the investigator, the subject's general health status declines to an extent that the risks of treatment outweigh the potential benefits.
8. ***Subjects do not tolerate the minimum allowed dose (refer to protocol 3.4.5)***[1]
9. ***Subjects show clinically significant out-of-range laboratory values, clinically significant abnormal findings on physical examination, or intolerable adverse events (AEs) that put him at additional risk, as judged by the investigator. For termination of treatment in case of drug induced liver injury (DILI) refer to section 6.2***[1].
10. MIN-102 is commercially available for the treatment of cALD.

Study Overview/Design

This is an open-label, multicenter study in male pediatric subjects, aged ≥2 and ≤12 years, with a diagnosis of the cALD phenotype of X-linked ALD. After written informed consent by the parent, legal guardian or authorized legal representative, and after completion of all screening evaluations with all inclusion criteria and no exclusion criteria being met, subjects will be treated with MIN-102 until the first occurrence of any of the end-of-treatment criteria described in the section "Duration of Treatment" above, or until initiation of HSCT induction procedures.

Each subject will undergo screening for a maximum of 15 days, followed by a treatment period. If the screening procedures are performed within 7 days prior to Baseline (V0), all results obtained from the screening evaluations will serve as the baseline values and the pre-dose baseline procedures will only consist of the clinical efficacy assessment (NFS-MFD), the blood sampling for biomarkers and MIN-102 plasma levels, and the optional CSF sampling for biomarkers. Blood and CSF (if applicable) sample remnants will be stored for the purpose of supporting future research for a maximum of 25 years. Consent to long-term storage is optional and requires additional informed consent.

Subjects will be evaluated at the Screening Visit, Baseline Visit when the first dose of study drug is administered, and at regular biweekly intervals thereafter until Visit 6 (Table 1). After Visit 6 (Week 12), evaluations will occur at Visit 7 (Week 18), Visit 8 (Week 24), and Visit 9 (Week 36), Visit 10

[1] ***Only applicable in France according to the current approved protocol version 6.2.***

(Week 48), Visit 11 (Week 72), Visit 12 (Week 96), and further at 24-week intervals until end-of-treatment criteria apply.

When patients meet the specified HSCT criteria, HSCT may be performed at any time point after the Baseline Visit.

HSCT criteria have to be present at two consecutive MRI assessments at least 12 weeks apart from each other. If HSCT criteria are met at the first visit, but not at the second visit, another visit will be scheduled 12 weeks after the second visit to confirm presence or absence of HSCT criteria. If patients meet HSCT criteria for the first time at Visit 10 (Week 48) or later, an unscheduled visit will occur 12 weeks (±15 days) afterwards. If HSCT criteria are not met at the unscheduled visit, the subject will continue with the regular visit schedule. If HSCT criteria are met at two consecutive visits, the subject will be scheduled for HSCT. However, the investigator may also terminate treatment if his/her overall assessment of MRI lesions mandates immediate scheduling of HSCT.

Subjects shortlisted for HSCT:

The subject will adhere to the visit schedule until initiation of HSCT procedures. Prior to initiation of HSCT procedures, the subject will be scheduled for another visit ("Visit Prior to HSCT") irrespective of the time since the Baseline Visit. This visit is the final visit in the study for subjects undergoing HSCT. The last dose of study drug may be administered on the day before the "Visit Prior to HSCT". The investigator may schedule the "Visit prior to HSCT" as close as up to 5 days prior to initiation of HSCT procedures, defined as the day of first administration of myeloablative medication. To lessen the burden for the subject, it is at the Investigator's discretion to cancel a visit within the regular schedule of visits, should such a visit be within 4 weeks from the "Visit Prior to HSCT".

If the subject is shortlisted for HSCT, but meets contraindications against HSCT, or HSCT is not performed due to unavailability of a donor, treatment with study drug may continue if decided by the PI. If treatment is stopped, all assessments equivalent to the "Visit Prior to HSCT" will be performed one day after last dose of study drug. The Follow-up Visit will be scheduled 4 weeks(±5 days) after last dose of study drug.

If HSCT criteria are not met:

Subject will adhere to the visit schedule with the assessments shown in Table 1. MIN-102 treatment will continue until the first occurrence of any of the end-of-treatment criteria defined in section 3.1.2. If a subject discontinues MIN-102 treatment prematurely or discontinues treatment per the end-of treatment criteria above, he will have a Follow-Up Visit 28 (± 5) days after the last administration of study drug. If a subject discontinues before planned HSCT, the Follow-up Visit should be performed 28 days after last administration of study drug, or immediately before initiation of HSCT procedures if these occur earlier than 28 days after last dose of study drug. Subjects undergoing HSCT will not have a Follow-up Visit.

An independent Data and Safety Monitoring Board (DSMB) will receive each subject's data after he has reached V8 at Week 24 or completed treatment with MIN-102 prior to HSCT (if earlier), or has completed the Follow-Up Visit. The DSMB may also review subject data ad hoc, as needed, in case of significant safety concerns. The DSMB can recommend stopping or modifying the study at any time if unacceptable safety risks become apparent or the number of dropouts is much higher than anticipated. The reasons for dropouts will be carefully investigated. The DSMB can also recommend a downward adjustment of the MIN-102 target exposure range if safety risks are identified. The composition and function of the DSMB has been described in a Charter signed by all DSMB members.

Study Population

This study will include patients with cALD who meet all inclusion criteria and none of the exclusion criteria listed below.

Inclusion Criteria

1. Written informed consent by parent/legal guardian, or authorized legal representative to participate in the study
2. Males aged ≥2 and ≤12 years with a diagnosis of X-linked ALD based on genetic testing; or, in absence of genetic testing, elevation of VLCFA and confirmed by family history of X-ALD with clinical symptoms and elevation of VLCFA or by genetic testing of a family member.
3. White matter involvement as determined by cerebral MRI lesions without Gd enhancement at baseline (Population 1), or with Gd enhancement at baseline (Population 2).
4. Major Functional Disabilities (MFD) score of 0, as determined by key measures in the Neurological Function Scale (NFS)
5. Baseline Loes score >0 and ≤10
6. Baseline Gadolinium Intensity Score (GIS) ≤3
7. No signs or symptoms of adrenal insufficiency and morning cortisol and aldosterone levels within normal laboratory ranges for age, or appropriate steroid replacement if adrenal insufficiency is present. A history of adrenal insufficiency is not exclusionary if the foregoing is currently met.
8. Glycated hemoglobin (HbA1c) within normal range

Exclusion Criteria

1. Other chronic neurological disease
2. Known intolerance to pioglitazone or other thiazolidinediones
3. Use of pioglitazone or other thiazolidinediones within the past 6 months prior to screening
4. Use of biotin at a daily dose of >50 mg per day within the past 3 months prior to screening
5. Current participation in another interventional clinical study or participation in such a study within 6 months prior to screening
6. Previous HSCT
7. Requirement for a prohibited concomitant medication
8. Previous or current history of bladder polyps, bladder cell hyperplasia, or cancer (other than successfully treated basal cell carcinoma)
9. ***Chronic or recurrent symptomatic urinary infections (≥2 per year over the past 2 years until Screening [V-1]).***[1]
10. ***Permanent indwelling urinary catheter or catheter port***[1]
11. ***Smoking with 25 cigarettes per day over the past 2 years until Screening (V-1***)[1]
12. Previous or current history of congestive heart failure
13. Clinically significant anemia with hemoglobin <10 g/dL

[1] ***Only applicable in France according to the current approved protocol version 6.2.***

14. Alanine aminotransferase (ALT) or aspartate aminotransferase (AST) level >2 times the ULN or total bilirubin >1.5 times the ULN (unless due to Gilbert's syndrome)
15. Moderate or severe hepatic impairment (groups B and C according to Child-Pugh classification)
16. eGFR < 90 ml/min or any evidence of renal disease or impairment, including proteinuria or hematuria
17. Pulmonary disease or cardiac disease of sufficient severity to limit participation in the study and/or completion of study procedures
18. Reduced left-ventricular ejection fraction or other clinically significant cardiac abnormalities on echocardiogram that, in the investigator's opinion, could predispose the subject to volume overload or its attendant consequences
19. Hereditary Fructose Intolerance
20. History of diabetes, or glycated hemoglobin (HbA1c) levels >6.4% and fasting blood glucose levels ≤ 0.9 times the lower limit of normal and ≥ 1.1 times the upper limit of normal at Screening
21. ***A positive result on laboratory tests for hepatitis B surface antigen, hepatitis C antibody or human immunodeficiency virus antibody***[1]
22. Contraindication to MRI procedure, such as presence of ferromagnetic materials (aneurysm clips, pacemaker, intraocular metal, cochlear implant) in the body
23. Conditions that could modify the absorption of the study drug
24. Inability or unwillingness of parent/legal guardian or subject to comply with the study procedures
25. Inability or unwillingness of parent/legal guardian or subject to resume standard of care at a local center once study is complete or criteria for HSCT is met and treatment available.
26. Other medical, neurologic, psychiatric, or social condition that, in the opinion of the investigator, is likely to unfavorably alter risk-benefit of study participation, confound interpretation of safety or efficacy results, or interfere with the satisfactory completion of study requirements

Statistics Overview

Interim assessment: Baseline to assessment after 6 months or last assessment immediately prior to HSCT, if earlier

Confirmatory assessment: Baseline to 2 years or last assessment immediately prior to HSCT, if earlier.

The study will enroll 13 subjects and use a criterion of at least 4 of 13 subjects achieving criteria for “arrested disease” at Week 24 and Week 96 with a one-sided significance level of 0.05. The study will have 80% power to distinguish a rate of 40% subjects with “arrested disease” with MIN-102 compared to a background rate of 10% as expected from natural history.

[1] ***Only applicable in France according to the current approved protocol version 6.2.***

Additional subjects may be enrolled after agreement between the investigator and the Sponsor to ensure that the study recruits 13 evaluable subjects who receive MIN-102 treatment. Decisions regarding subject replacement will be documented. Patients who meet HSCT criteria and patients who at week 96 do not meet "arrested" disease criteria will not be replaced. However, patients who drop out by withdrawal of consent by parents for non-treatment related causes may be replaced. Patients dropping out will be encouraged to remain in the study and attend all further scheduled efficacy and safety assessments.

Generally, outcome variables will be evaluated for change from baseline to the designated time point using descriptive statistics (N, mean, SD, median, minimum, and maximum). In addition, categorical outcomes such as first appearance of Gd-enhancing cerebral lesions in subjects not showing these lesions at baseline, overall survival for evaluable patients at Week 96 (V12) and number of patients meeting HSCT criteria will be analyzed using frequencies tables. The analyses of endpoints will include correlation analyses and graphical presentations.

Planned Study Timelines:

Start clinical phase:	September 2019
End clinical phase:	June 2024
Database lock:	July 2024
Top Line Results:	August 2024
Final report complete:	October, 2024

Figure 1. Study Design

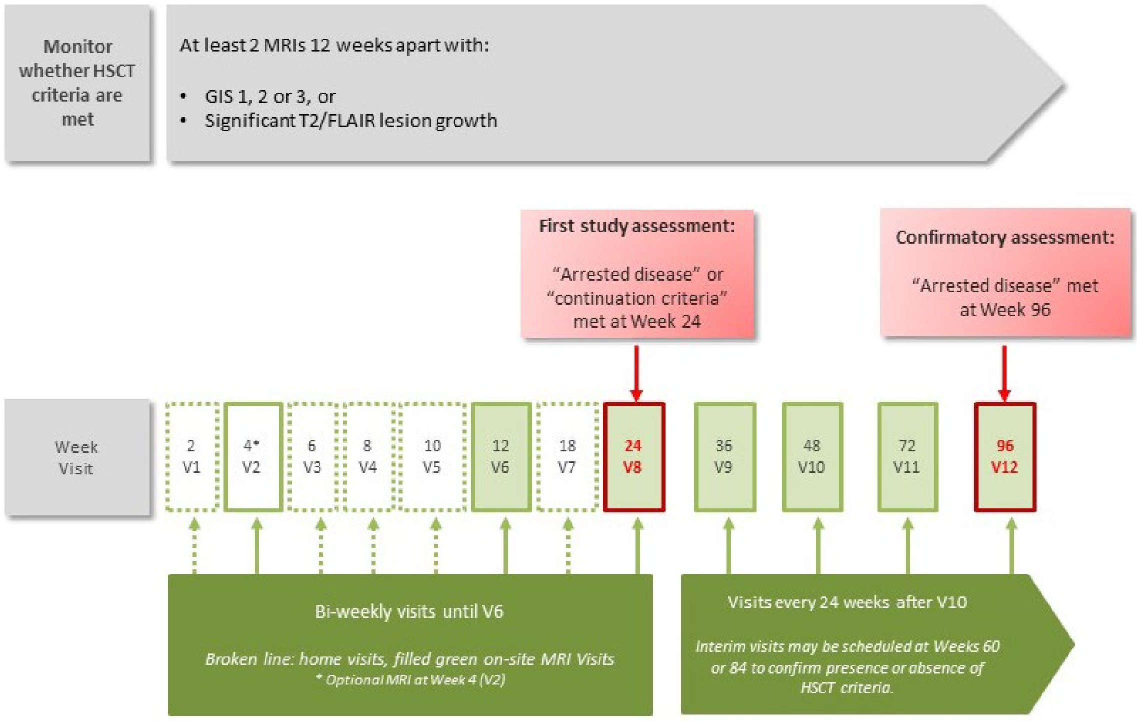

Table 1. Flow Chart of Study Procedures

Visit Number	-1	0[1]	1 *	2	3 *	4 *	5 *	6	7*	8[3]	9-12+		
**Visit Name**	**Screening**	**Baseline**	**Visit 1**	**Visit 2**	**Visit 3**	**Visit 4**	**Visit 5**	**Visit 6**	**Visit 7**	**Visit 8**	**Visit 9, 10, 11, 12[3]...**	**Visit Prior to HSCT[2]**	**FUV [4]**
**Week**	**-2**	**0**	**2 (±5 days)**	**4 (±5 days)**	**6 (±5 days)**	**8 (±5 days)**	**10 (±5 days)**	**12 (±5 days)**	**18 (±15 days)**	**24 (±15 days)**	**36, 48, 72, 96, ..[3] (±15 days)**	**To be scheduled before starting HSCT procedures**	**4 weeks after last dose (±5 days)**
Informed consent	X												
Inclusion & exclusion criteria	X	X [1,5]											
Medical history/concomitant disease	X	X[1, 5]											
Demographics	X												
Prior medication	X	X[1, 5]											
Vital signs (weight, height, BP, pulse rate, & temperature)[6]	X		X	X	X	X	X	X	X	X	X	X	X
Physical examination[20]	X		X	X	X	X	X	X	X	X	X	X	X
12-lead ECG (recorded in triplicate)[7]	X	X		X		X		X		X	X	X	X
Echocardiogram [8]	X												
NFS-MFD		X						X		X	X	X	X
Hematology, blood chemistry, PT [9]	X	X	X	X	X	X	X	X	X	X	X	X	X
Urinalysis, urine cytology	X			X				X		X	X	X	X
Virology testing for hepatitis B, hepatitis C and human immunodeficiency virus (HIV)[a]	X												

[a] ***Only applicable in France according to the current approved protocol version 6.2.***

Visit Number	-1	0[1]	1 *	2	3 *	4 *	5 *	6	7*	8[3]	9-12+		
**Visit Name**	**Screening**	**Baseline**	**Visit 1**	**Visit 2**	**Visit 3**	**Visit 4**	**Visit 5**	**Visit 6**	**Visit 7**	**Visit 8**	**Visit 9, 10, 11, 12[3]…**	**Visit Prior to HSCT[2]**	**FUV [4]**
**Week**	**-2**	**0**	**2 (±5 days)**	**4 (±5 days)**	**6 (±5 days)**	**8 (±5 days)**	**10 (±5 days)**	**12 (±5 days)**	**18 (±15 days)**	**24 (±15 days)**	**36, 48, 72, 96, ..[3] (±15 days)**	**To be scheduled before starting HSCT procedures**	**4 weeks after last dose (±5 days)**
Blood sampling for MIN-102 levels [10]		X		X				X		X	X	X	
Blood sampling for biomarkers [11]		X		X				X		X	X	X	
CSF sample for biomarkers [12]		X						X			X[21]	X	
Blood spot glucose check[13]		X	X	X	X	X	X	X	X	X	X	X	
Cerebral MRI	X[14]			X[15]				X		X[16]	X	X[16]	
Study drug dispensation[17]		X						X		X	X		
Study drug accountability				X				X		X	X	X	
Subject diary review[18]			X	X	X	X	X	X	X	X	X	X	X
Palatability assessment[19]		X		X				X		X	X		
Adverse event recording		X	X	X	X	X	X	X	X	X	X	X	X
Concomitant medication		X	X	X	X	X	X	X	X	X	X	X	X

*** Option for home visit.**

NT-proBNP = NT-proB-type Natriuretic Peptide; BP = blood pressure; CSF = cerebrospinal fluid; ECG = electrocardiogram; FUV = Follow-up Visit; HbA1c = glycated hemoglobin; HSCT = hematopoietic stem cell transplantation; MRI = magnetic resonance imaging; NFS-MFD = Neurological Function Score- Major Functional Disabilities; PT = prothrombin time; V = Visit; ULN = upper limit of normal

1 No separate baseline procedures are performed for those procedures that were already performed at the Screening Visit (V -1). All results obtained at the Screening Visit (V -1) will serve as the baseline values. Except for post-dose blood sampling for MIN-102 levels- post-dose ECG and palatability assessment, all assessments at the Baseline Visit (V0) are to be performed pre-dose.
2 If the "Visit Prior to HSCT" is scheduled within 4 weeks to another study visit, the Investigator may decide to omit the other study visit and perform only the "Visit Prior to HSCT" in its place. This is the end of study visit for subjects undergoing HSCT and will be scheduled up to 5 days prior to initiating HSCT procedures.
3 Unscheduled visits may occur at Weeks 60 and 84 to confirm presence or absence of HSCT criteria. Patients will continue visit schedule until any of the end-of-treatment criteria apply.

4 The Follow-up Visit will be scheduled 28 (±5) days after last dose of MIN-102 for subjects who discontinue MIN-102 treatment after a decision not to undergo HSCT. If a subject drops out of the study before HSCT, the Follow-up Visit will be performed 28 (± 5) days after the last administration of study drug, or immediately before initiation of HSCT procedures, if these occur earlier than 28 days after last dose.
5 Confirmation and/or update of the screening information.
6 Temperature will be measured at each visit until HSCT procedures are initiated
7 At the Baseline Visit, ECGs will be recorded pre-dose and 2 hours (±0.5 hours) after administration of MIN-102. At all other visits, ECGs will be recorded 2 hours (±0.5 hours) after administration of MIN-102.
8 Echocardiogram will be repeated only in subjects developing peripheral edema, or elevated NT-proBNP >1.5 times the Baseline value and out of normal range; in such instances, the echocardiogram will be repeated at each visit, until resolution of the peripheral edema or elevated NT-proBNP.
9 At the Screening Visit (V-1), the full panel of laboratory parameters will be assessed except for NT-proBNP and prothrombin time, which will be assessed at the Baseline Visit (V0). At Visits 1, 3, 4, 5 and 7, only total bilirubin, alkaline phosphatase, aspartate aminotransferase, alanine aminotransferase, and prothrombin time will be assessed. At all other visits, the full panel of laboratory parameters will be assessed. Adrenal function chemistries (ACTH, cortisol, aldosterone) will only be determined at V-1, and HbA1c will be determined only at V-1 and "Visit Prior to HSCT" or FUV. For patients whose body weight is lower than 13.75Kg (30.31 Lbs.), prothrombin time will only be analyzed if liver parameters show alterations suspicious of drug induced liver injury (DILI).
10 At the Baseline Visit (V0) blood samples will be drawn pre-dose, and 2, 6, 12, and 24 hours post-dose requiring an overnight stay. At Visits V2 and V6, respectively, blood samples will be drawn pre-dose, and 2 hours post-dose. At further visits, blood samples for MIN-102 levels will be drawn immediately before MIN-102 administration. At the Visit Prior to HSCT, only one sample will be drawn.
11 Blood samples for biomarker analysis will always be taken pre-dose.
12 Cerebrospinal fluid sampling for biomarkers is optional. The sample at the Baseline Visit (V0) should be taken any time before the first dose. CSF sampling should be performed while the patient in sedated during the MRI, if possible.
13 Blood spot glucose test to be performed at 2 hours (±0.5 hours) after administration of daily dose.
14 The MRI parameters obtained at the Screening Visit (V-1) will be considered the baseline values for the evaluations. If the MRI cannot be scheduled during the Screening Visit, it will be performed at the Baseline visit (V0) prior to first dose of study medication. If a valid MRI obtained with the same study-specific specifications and within 15 days before Screening is available, this will be considered the Screening MRI and no extra MRI will be performed.
15 MRI is optional.
16 To minimize subjects' exposure to sedation for the MRI and/or exposure to gadolinium, the Investigator may omit post-baseline scheduled MRIs at a given visit if they are not deemed necessary to monitor progression of cerebral lesions, except for V8 and/or the "Visit prior to HSCT".
17 For subjects who are eligible to HSCT, but HSCT has not been performed, study drug is dispensed every 12 weeks; for subjects with a final decision not to perform HSCT and continuing treatment, study drug is dispensed every 24 weeks.
18 A subject diary will be used in the study to keep track of IMP compliance, recording adverse events and any changes in concomitant medications
19 To be performed by child or parent depending on the age and cognitive ability.
20 At V1, V3, V4, V5, and V7 the patient will be only examined for the presence of peripheral edema
21 Only at Visit 12.

TABLE OF CONTENTS

AUTHORIZATION OF CLINICAL STUDY PROTOCOL 2
SERIOUS ADVERSE EVENT CONTACT INFORMATION 4
CONTACT INFORMATION..... 5
SYNOPSIS 7
TABLE OF CONTENTS 19
TABLE OF TABLES 22
TABLE OF FIGURES 22
LIST OF ABBREVIATIONS 23
1. INTRODUCTION 26
1.1. Overview of Cerebral Adrenoleukodystrophy 26
1.2. Study Drug 26
1.2.1. Nonclinical Pharmacology 27
1.2.2. Nonclinical Pharmacokinetics 28
1.2.3. Nonclinical Safety 28
1.3. Clinical Experience 29
1.3.1. Phase 1 Study MT-1-01 29
1.3.2. Phase 2/3 Study MT-2-01 31
1.4. Study Rationale 33
1.5. Risk benefit assessment 34
2. OBJECTIVES 35
2.1. Primary Objective 35
2.2. Secondary Objectives 35
2.3. Exploratory Objectives 35
3. INVESTIGATIONAL PLAN 36
3.1. Overall Study Design and Plan 36
3.1.1. Visits and Assessments 37
3.1.2. Study termination 43
3.1.3. Data Safety Monitoring Board 45
3.2. Discussion of Study Design 45
3.3. Selection of Study Population 47
3.3.1. Inclusion Criteria 47
3.3.2. Exclusion Criteria 48
3.4. Treatment 49
3.4.1. Treatments Administered 49
3.4.2. Identity of Investigational Product 49
3.4.3. Selection of Dose and Dose Adjustment Criteria 50

3.4.4. Dose Reductions for Safety or Tolerability Reasons 51
3.4.5. Study Drug Administration and Instructions for Subjects. 52
3.4.6. Study drug interruptions....52
3.4.7. Treatment Compliance and Study Drug Accountability 52
3.4.8. Packaging, Labeling and Resupply....53
3.4.9. Storage Conditions 53
3.4.10. Concomitant Medications....53
3.5. Study Assessments and Variables 54
3.5.1. Efficacy Assessments 54
3.5.2. Pharmacokinetics and Biomarkers Measurements....55
3.5.3. Assessment of Safety and Tolerability Measurements....56
3.5.4. Total Blood sampling volumes 59
3.6. Statistics....60
3.6.1. Analysis Sets 60
3.6.2. Interim Analysis....63
3.6.3. Determination of Sample Size 64
4. STUDY MANAGEMENT 64
4.1. Approval and Consent....64
4.1.1. Regulatory Guidelines 64
4.1.2. Institutional Review Board (IRB)/Independent Ethic Committee (IEC) 64
4.1.3. Written Informed Consent 65
4.2. Financing and Insurance....65
4.3. Discontinuation of the Study by the Sponsor 65
4.4. Changes to Final Study Protocol....65
4.5. Notification of Study Completion or Discontinuation....66
4.6. Quality Assurance and Quality Control 66
4.6.1. Study Monitoring....66
4.6.2. Audits and Inspections 67
4.6.3. Data Collection....67
4.6.4. Data Management 67
4.6.5. Storage and Retention of Study Records 68
4.6.6. Subject Confidentiality 69
4.7. Use of Study Findings 69
4.8. Publication Policy....69
5. LIST OF REFERENCES 70
6. APPENDICES....72
6.1. Adverse Events 72

6.1.1. Definitions 72
6.1.2. Recording and Reporting Adverse Events 73
6.1.3. Regulatory Agencies, Institutional Review Board (IRB)/Independent Ethic Committees (IEC), and DSMB Reporting 74
6.1.4. Follow-up of Adverse Events 75
6.2. Drug-Induced Liver Injury Management 75
6.2.1. Introduction 75
6.2.2. DILI Monitoring Schedule 75
6.2.3. DILI Follow-up 75
6.2.4. Communication Flow 76
6.3. Detailed blood sampling volumes 78
6.4. Neurological Functional Score – Major Functional Disabilities (NFS-MFDs) scale 79
6.5. NFS scale item definitions for standardized rating 80
6.6. Rationale of the amendment 82
6.7. Summary of changes 85

TABLE OF TABLES

Table 1. Flow Chart of Study Procedures 16

Table 2. Percent Change in Lesion FLAIR Over Time per-HSCT and After HSCT From University of Minnesota Database 46

Table 3. Composition of Study Medication....50

Table 4. Starting Doses MIN-102....51

Table 5. Permitted and Prohibited Concomitant Medications 54

Table 6. Total Blood sampling volumes per patient....60

Table 7. Analysis Sets....60

Table 8. Total Blood sampling volumes per visit per patient....78

TABLE OF FIGURES

Figure 1. Study Design 15

LIST OF ABBREVIATIONS

Abbreviation	Definition
ADC	apparent diffusion coefficient
AE	adverse event
ALD	adrenoleukodystrophy
ALT	alanine aminotransferase
AMN	adrenomyeloneuropathy
AST	aspartate aminotransferase
AUC	area under the concentration-time curve
AUC_{0-24}	AUC from time 0 to 24 hours
AUC_{∞}	AUC from time 0 extrapolated to infinity
AUC_t	AUC from time 0 to end of dosing period
NT-proBNP	NT-proB-type Natriuretic Peptide
cALD	cerebral adrenoleukodystrophy
C_{max}	maximum plasma concentration
C_{min}	minimum plasma concentration
CFR	Code of Federal Regulations
CSF	cerebrospinal fluid
CTH	Capillary Mean Transient Time Heterogeneity
CYP	cytochrome P450
DILI	drug-induced liver injury
DSC	dynamic susceptibility contrast
DSMB	Data Safety Monitoring Board
DTI	diffusion tensor imaging
ECG	electrocardiogram
eCRF	electronic case report form
EDC	Electronic data capture
eGFR	Estimated Glomerular Filtration Rate
FA	fractional anisotropy
FABP4	fatty acid binding protein 4
FDA	Food and Drug Administration
FE	food effect
FLAIR	fluid attenuation inversion recovery
GCP	Good Clinical Practice
Gd	gadolinium
GIS	gadolinium intensity score

Abbreviation	Definition
HbA1c	glycated hemoglobin
HSCT	hematopoietic stem cell transplantation or transplant
ICH	International Conference on Harmonisation
IL	interleukin
IND	Investigational New Drug Application
IP-10	interferon gamma-induced protein 10
IRB	Institutional Review Board
ITT	intent-to-treat
IV	Intravenous
Kapp	Constant of gadolinium permeability using advance perfusion software
K2	Constant of gadolinium permeability using conventional perfusion software
Lenti-D	Lenti-D lentiviral vector
LLOQ	lower limit of quantitation
MAD	multiple ascending dose
MCP-1	monocyte chemoattractant protein-1
MFD	Major Functional Disabilities
MRI	magnetic resonance imaging
NFS	Neurological Function Scale
NOAEL	no-observed-adverse-effect level
NfL	Neurofilament light chain
PBPK	physiologically based PK
PI	Principal Investigator
PK	pharmacokinetic or pharmacokinetics
PP	per-protocol
PPAR	peroxisome proliferator-activated receptor
PT	preferred term
QTcF	QT interval corrected for heart rate using Fridericia's formula
SAD	single ascending dose
SAE	serious adverse event
SD	standard deviation
SoC	Standard of Care
SUSAR	suspected unexpected serious adverse reaction
ULN	upper limit of normal
TBIL	total bilirubin
TEAE	treatment-emergent adverse event

**Abbreviation**	**Definition**
TESAE	treatment-emergent serious adverse event
U.S.	United States
VLCFA	very long chain fatty acids

1. INTRODUCTION

1.1. Overview of Cerebral Adrenoleukodystrophy

Cerebral adrenoleukodystrophy (cALD) is the most rapidly progressing and devastating phenotype of X-linked adrenoleukodystrophy (ALD). Age of onset is typically between 2 and 10 years, but may be later, including during adulthood. The first clinical symptoms of cALD are behavioral and/or learning deficits linked to a decline in kindergarten or school performance. With disease progression, more serious symptoms appear, including withdrawal or inattention, hyperactivity, apraxia, difficulty in understanding speech, difficulty in reading, hemiparesis or spastic quadriplegia, cerebellar ataxia, and seizures. After this stage, the progression of the disease accelerates rapidly, and within 6 months to 2 years, patients become completely disabled, typically dying 2 to 4 years after onset of the symptoms.

The only accepted treatment for cALD is HSCT. A similar procedure, the administration of autologous CD34+ hematopoietic stem cells, transduced ex vivo with Lenti-D lentiviral vector (Lenti-D), is currently under clinical development. Like HSCT, this procedure requires complete myeloablation with all the associated risks, although autologous transplantation obviates the need to find a suitable donor. The decision to schedule HSCT or an equivalent procedure is made after appearance of inflammatory, Gd-enhancing MRI lesions, and/or upon T2/FLAIR lesion growth. However, myeloablation and HSCT are burdened with a significant mortality risk. The estimated probability of survival 5 years post-HSCT is only 75%, with 38% of deaths due to disease progression, and transplant-related mortality 100 days post-transplant is 8% (Miller et al., 2011). Additionally, HSCT does not prevent the development of adrenomyeloneuropathy later in life (Engelen et al. 2012). Complication rates, long-term outcomes, and the potential for future regulatory approval of Lenti-D treatment are currently not known.

The clinical outcome of HSCT is significantly improved if the procedure is carried out before patients develop more severe MRI lesions (Miller et al., 2011; Engelen et al., 2012). More intense pre-transplant Gd enhancement has been shown to predict poorer clinical status by Neurologic Function Score (Miller et al., 2016). In addition, higher MRI severity scores at the time of transplant have been shown to be negatively correlated with better neurocognitive performance post-transplant (Pierpont et al., 2017).

Spontaneous arrest of the disease is rare in cALD, particularly in children aged up to 12 years. Depending whether radiological or clinical parameter are used, rates of self-arrest are estimated to be 10% or lower (Moser et al., 2000; Engelen et al., 2012, Eichler, 2016; Raymond et al., 2019; Liberato et al., 2019).

Thus, there is a high unmet medical need for a treatment that prevents or delays the onset of cerebral inflammatory lesions, reduces the speed of lesion progression, and improves clinical outcome after HSCT.

1.2. Study Drug

The study drug, 5-[[4-[2-[5-(1-hydroxyethyl)-2-pyridinyl]ethoxy]phenyl]methyl]-2,4-thiazolidinedione hydrochloride (company code: MIN-102; INN: leriglitazone), is a differentiated peroxisome proliferator-activated receptor γ (PPARγ) agonist. MIN-102 is a metabolite of pioglitazone, an approved treatment for type II diabetes. Approximately 25% of pioglitazone is metabolized to MIN-102 (also known as M4) and then on to the metabolite 5-[[4-[2-(5-acetyl-2-pyridinyl)ethoxy]phenyl] methyl]-2,4-thiazolidinedione (also known as M3) (MIN-102 Investigator's Brochure).

The study drug is supplied as an oral aqueous-based suspension and is administered orally once daily (1 mL of oral suspension contains 13.66 mg of leriglitazone, 15 mg of leriglitazone hydrochloride). Company code MIN-102 is used to refer to the salt of leriglitazone (as hydrochloride) when administered, however, when reporting plasma or cerebrospinal fluid (CSF) concentrations/exposure MIN-102 refers to the free base (leriglitazone).

In binding assays, MIN-102 associated with PPARγ with a half maximal inhibitory concentration of 7.3×10^{-6} M but did not associate with PPARα or PPARδ or various other receptors, ion channels, or transporter molecules.

1.2.1. Nonclinical Pharmacology

PPARγ agonists have shown potential in animal models that assess various symptoms associated with ALD and are the only class of agent in development for potential use across the main symptoms of the condition. PPARγ agonists have also shown efficacy in models of neurodegenerative disease. For further information, please refer to the Investigator's Brochure (MIN-102 Investigator's Brochure).

Pioglitazone induces neuroprotective and restorative effects in several preclinical models of neurodegenerative disease with motor dysfunction (single Abcd1- and double Abcd1-/Abcd2-/- knockout mice) and neuroinflammatory processes. This supports a role for PPARγ agonists in ALD treatment (Feinstein et al., 2002; Hunter et al., 2007; Ramkalawan et al., 2012; MIN-102 Investigator's Brochure). Similar effects have been seen with MIN-102, which was able to protect motor neurons and astrocytes from very long chain fatty acid (VLCFA)-induced toxicity, effect a dose-dependent improvement in disability score in mice with experimental autoimmune encephalitis, and restore mitochondrial expression of genes associated with biogenesis, reduced oxidative damage, and reversed proinflammatory status in rat spinal cord tissue (MIN-102 Investigator's Brochure). A 15% reduction in VLCFA is also seen in Abcd1 knock-out mice, due to a reduction in levels of an enzyme involved in synthesis; it is difficult to determine whether the relatively small decrease in VLCFA would have beneficial effects. However, the robust improvements in mitochondrial biogenesis and changes in antioxidant and anti-inflammatory markers levels may have an impact on ALD pathophysiology.

Beneficial effects of pioglitazone, MIN-102, and other PPARγ analogues in preclinical models of neuroinflammation were associated with direct stimulation of PPARγ in microglia and neurons and reduction of the neuroinflammatory response and oxidative stress (Hunter et al., 2007; Ramkalawan et al., 2012).

Based on preclinical data, pioglitazone was tested in clinical studies of amyotrophic lateral sclerosis, Alzheimer's disease, Parkinson's disease, and Friedreich's ataxia. Studies performed to date have not shown clinical benefit. However, the pioglitazone doses studied were in the range used to manage diabetes, for which the circulating levels achieved were 2 to 4 times lower than those with activity in preclinical studies.

MIN-102 is an active metabolite of pioglitazone and contributes a majority of the pharmacological activity of administered pioglitazone. It is suggested that MIN-102 may show greater efficacy in neuroinflammatory disease than pioglitazone, as metabolic conversion is not needed to produce the active agent.

1.2.2. Nonclinical Pharmacokinetics

MIN-102 is the main pioglitazone metabolite in species tested in toxicology studies. MIN-102 accounts for about 18% of pioglitazone metabolites in the rat; in dogs MIN-102 has an area under the concentration-time curve (AUC) that is 2.7 times greater than that of pioglitazone, and in humans MIN-102 has an AUC that is 2 to 3 times greater than that of pioglitazone (MIN-102 Investigator's Brochure). These species are, therefore, significantly exposed to MIN-102 during pioglitazone treatment.

MIN-102 has a simpler metabolic profile than pioglitazone: MIN-102 primarily metabolizes to M3 and both compounds are excreted; pioglitazone is metabolized to 6 different metabolites. After pioglitazone dosing in human subjects, exposure to M4 (MIN-102) is approximately 2 times higher than the parent compound, with both agents having a similar contribution to efficacy.

Various preclinical studies have shown that MIN-102 has lower cytochrome P450 inhibition/induction activity than pioglitazone (MIN-102 Investigator's Brochure), and is therefore likely to have a lower tendency to interact with other drugs.

1.2.3. Nonclinical Safety

MIN-102 is a well-characterized metabolite of pioglitazone (described as M4 in pioglitazone studies). A reduced toxicology assessment strategy was agreed with the European Medicines Agency (EMEA/H/SA/2862/1/2014/SME/III) based on the understanding that both animals and patients have been extensively exposed to study drug during pioglitazone treatment. Safety pharmacology for MIN-102 has been evaluated by considering pioglitazone literature data and MIN-102 stand-alone studies.

It is not expected that new safety findings will arise from MIN-102 studies compared with results from prior pioglitazone experience. Known side effects of pioglitazone are fluid retention, weight increase, and increased bone fracture in females; there are some suggestions that bladder cancer may be associated with long-term administration. Details on the safety profile of pioglitazone can be found in the summary of product characteristics for pioglitazone (Actos® SmPc 09 June 2016).

A 28-day repeat-dose toxicity study comparing MIN-102 with pioglitazone has been performed in rats (MIN-102 Investigator's Brochure). The no-observed-adverse-effect levels were 14.5 and 25 mg/kg for pioglitazone and MIN-102 respectively. At the NOAEL for MIN-102, the AUC from time 0 to time 24 hours (AUC_{0-24}) at steady state was 226 µg•hr/mL for males and 250 µg•hr/mL in females. Similar results were observed in male rats in a 13-week repeat dose toxicity study; NOAELs for MIN-102 and pioglitazone were not determined for female rats in this study due to changes in the reproductive tract. A 6-month repeat dose toxicity study in adult rats showed a lower NOAEL for MIN-102 than was observed in the 13-week or 28-day study (12.5 mg/kg; AUC from time 0 to end of dosing period (AUC_t) = 108 µg•hr/mL for males and 146 µg•hr/mL for females); reductions in NOAEL levels over time are as expected for PPARγ agonists. At higher doses in the 6-month study, a clear effect of MIN-102 on red cell parameters suggestive of hemodilution and increased heart weights (~20% greater than control group) not accompanied by microscopic cardiac changes were the most salient findings and suggested that the death of several rats at higher doses may be attributed to heart failure, a known PPARγ agonist safety concern in toxicology species and humans. In a 10-week toxicity study in juvenile rats, the NOAEL exposure in terms of AUC_{0-24} was approximately 168 µg•hr/mL for males and 240 µg•hr/mL for females.

All MIN-102-related findings in the repeated-dose toxicity studies in adult rats (4, 13 and 26 weeks) and in juvenile rats (10 weeks) and dogs (3 and 9 months) were similar to findings observed in rats and dogs administered pioglitazone. No unexpected new toxicities were observed in rats and dogs administered MIN-102. Thus, higher doses of both compounds did not show differences in toxicology findings, which were consistent with the safety profile of other PPARγ agonists. Additionally, no decreases in glucose plasma levels were observed.

In vitro genotoxicity studies with MIN-102 demonstrated lack of genotoxic potential. No relevant toxicity findings have been identified for respiratory, central nervous, or cardiovascular systems in animal models. (MIN-102 Investigator's Brochure). Therefore, the human target exposure of 170 µg•hr/mL (±20%) is expected to confer efficacy without exposing subjects to undue risk.

1.3. Clinical Experience

1.3.1. Phase 1 Study MT-1-01

This was a phase 1, randomized, double-blind, placebo-controlled, single-center clinical study in healthy male volunteers, divided into 3 parts: a single ascending dose (SAD) part A with 3 dose levels (30, 90, and 270 mg MIN-102) followed by a food effect (FE) part (SAD/FE part); a multiple ascending dose (MAD) part B in a parallel design with 2 dose levels (135 or 270 mg MIN-102); and an open-label MAD part C in a parallel design with 2 dose levels (135 or 270 mg MIN-102) including cerebrospinal fluid (CSF) collection. Part A of the study comprised the first administration of MIN-102 in man.

A total of 33 subjects were included in the study: 9 subjects aged between 21 and 50 years in Part A, 18 subjects aged between 19 and 54 years in Parts B1 and B2, and 6 subjects aged between 26 and 51 years in Part C. Of the 24 subjects in Parts B and C, 12 subjects received MIN-102 in Part B and 6 subjects in Part C. Six subjects in Part B were randomized to placebo. However, due to a dispensing error, 1 subject in Part B who was randomized to placebo received MIN-102 during the second half of the dosing period, and 1 subject who was randomized to MIN-102 received placebo during the second part of the dosing period. These two subjects were included in the safety analyses but were excluded from pharmacokinetic evaluations. One subject discontinued during the study (Part A) for personal reasons and 32 subjects completed the study.

1.3.1.1. Safety

There were no serious adverse events (SAEs) or suspected unexpected serious adverse reactions (SUSARs) in the healthy-volunteer study. All treatment-emergent adverse events (TEAEs) were of mild severity and resolved completely. No clinically significant changes occurred in safety laboratory values, electrocardiograms (ECGs), or vital signs.

1.3.1.2. Pharmacokinetics

1.3.1.2.1. Plasma Concentrations

Part A (SAD):

Following administration of a single oral dose of MIN-102 under fasting conditions in male subjects, MIN-102 first appeared in plasma after 0.25 hours (at first post-dose sampling time point) for all subjects at all dose levels tested. Based on descriptive statistics, the highest geometric mean MIN-102 concentrations were observed between 0.25 and 2.5 hours post-dose and increased proportionally with

dose. After reaching a maximum, the concentrations of MIN-102 showed a steady decline. Combined individual MIN-102 plasma concentration-time profiles showed modest inter-subject variation within dose levels.

Consumption of a standard high-fat breakfast of 918 kcal after oral administration of 90 and 270 mg MIN 102 resulted in a clear delay in MIN-102 uptake. Maximum plasma concentrations (C_{max}) of MIN-102 were reached after 3 to 6 hours. However, the extent of exposure (AUC) was essentially not different from the fasting state. Therefore, MIN-102 dosing in further studies will be conducted with food.

Part B and C (MAD):

Plasma concentration-time profiles for MIN-102 following the first dose of 135 or 270 mg were similar to the profiles after a single dose in fed conditions. Following the first oral dose, MIN-102 appeared in plasma after 0.25 hours (the first sampling time point) for all subjects at all dose levels tested. Maximum MIN-102 concentrations were reached after 0.25 to 4 hours for 135 mg MIN-102 and between 2.5 and 8 hours for 270 mg MIN-102. On Days 2 to 7, concentrations of MIN-102 and M3 both before dosing and 4 hours post-dose increased daily. On Day 8, the concentration-time profiles had comparable shapes to Day 1 but with higher concentrations. Maximum MIN-102 concentrations were reached at similar times as seen with the single dose, at 0.5 to 4 hours after 135 mg MIN-102 and 4 to 8 hours after 270 mg MIN-102. Maximum concentrations on Day 8 were approximately 1.7 times higher than on Day 1 (135 mg MIN-102, relative C_{max} [R_{Cmax}] 1.73; 270 mg MIN-102, R_{Cmax} 1.72). When considering AUC, the accumulation of MIN-102 was approximately 1.7 (135 mg MIN-102, relative AUC [R_{AUC}] 1.70; 270 mg MIN-102, R_{AUC} 1.81).

1.3.1.2.2. Physiologically Based Pharmacokinetic Modeling

A physiologically based pharmacokinetic modeling (PBPK) model for MIN-102 (Simcyp MIN-102 Final Report 19-Oct, 2018) that incorporates CYP3A4 and CYP2C8-mediated metabolism as well as biliary clearance derived from in-vitro data was developed by Certara using the Simcyp software. During model development, the single-ascending dose (SAD) data were used as the model training dataset and the multiple-ascending dose (MAD) data were used as the model verification dataset. The findings show that CYP induction was low and insignificant. The final MIN-102 model was prospectively applied to estimate an appropriate starting dose for pediatric clinical evaluation. The default Simcyp ontogeny functions for CYP3A4 and CYP2C8 as well as literature ontogeny functions (Upreti and Wahlstrom, 2016) were applied in separate simulations. Allometric scaling was also applied to scale doses based on body size and compared to the PBPK derived doses. Since application of the Simcyp default ontogeny versus the Upreti ontogeny functions did not show significant difference in the projected pediatric doses, the Upreti ontogeny were selected as more appropriate for calculating the starting doses.

1.3.1.3. Cerebral Spinal Fluid Concentrations

No quantifiable MIN-102 was detected in pre-dose CSF samples, but MIN-102 was measured in CSF at the post-dose sampling time point for both doses. Geometric mean concentrations of MIN-102 were 188 ng/mL (range 158 to 218 ng/mL) 4 hours after the last dose of 135 mg MIN-102 on Day 8, and 332 ng/mL (range 287 to 376 ng/mL) after 270 mg MIN-102. No clear correlation between MIN-102 plasma concentrations at the time of the CSF sampling and CSF concentrations was seen. Concentrations of MIN-102 in CSF, however, seem to correlate with the C_{max} and AUC for MIN-102.

1.3.1.4. **MIN-102 in Urine**

Only limited amounts of MIN-102 were excreted in urine. Total excretion did not increase above 0.4% of the administered dose over the 48-hour collection period (range 0.1 to 0.4%) at any dose level and did not appear to vary with dose but varied between subjects. Consumption of breakfast did not change the amount of MIN-102 excreted.

1.3.1.5. **Pharmacodynamics Effects of MIN-102 in Plasma and CSF**

Mean plasma concentrations of adiponectin, an indicator of PPARγ receptor engagement, showed a clear increase from Day 1 to Day 8. Adiponectin increased from 4,532 ng/mL at baseline (Day 1 pre-dose) to 13,907 ng/mL (1 hour post-dose on Day 8) after dosing with 135 mg MIN-102 for 8 days and from 5,014 ng/mL to 22,688 ng/mL 6 hours post-dose on Day 8 after dosing with 270 mg MIN-102 for 8 days. No change in adiponectin concentrations was observed after 8 days of placebo dosing.

Fatty acid binding protein 4 (FABP4) concentrations showed some variation over time after placebo administration over the sampling period, with mean values ranging from 7,082 pg/mL at baseline to 8,880 pg/mL pre-dose on Day 8, but no trend was observed. After MIN-102 administration for 8 days the mean plasma concentrations for FABP4 increased from 8,839 pg/mL pre-dose on Day 1 to 15,587 pg/mL on Day 8 pre-dose at 135 mg MIN-102 and from 9,275 pg/mL pre-dose on Day 1 to 16,726 pg/mL on Day 8 pre-dose at 270 mg MIN 102.

Plasma concentrations of Interferon gamma-induced protein 10 (IP-10, also known as CXCL10) showed a slight decrease after 8 days of MIN-102 dosing. Both doses showed similar mean values at Day 8 (216 pg/mL at the 135 mg dose level, and 185 pg/mL at the 270 mg dose level) that were clearly lower than the values found in the placebo group (297 pg/mL).

Clear increases were seen for adiponectin from baseline to Day 8. Mean adiponectin concentrations increased from 6.0 ng/mL to 9.4 ng/mL at the 135 mg dose level, and from 5.8 ng/mL to 9.8 ng/mL at the 270 mg dose level. While there was no visible increase in mean FABP4 concentrations at the 135 mg dose level (baseline: 0.74 ng/mL, Day 8: 0.76 ng/mL), a clear increase from 0.75 ng/mL at baseline to 1.18 ng/mL was apparent at the 270 mg dose level. Some trends were observed for decreases in cytokines and chemokines. Interleukin (IL)-8 in CSF showed a decrease after 8 days of dosing with 135 mg and 270 mg MIN-102. Mean baseline concentrations decreased from 36.3 pg/mL to 33.7 pg/mL, and from 45.7 pg/mL to 33.7 pg/mL to Day 8 in the 135 mg and the 270 mg dose groups, respectively.

Concentrations of monocyte chemotactic protein 1 (MCP-1) in CSF showed decreases for both dose groups: from 591 pg/mL baseline to 458 pg/mL at Day 8, and from 799 pg/mL to 476 pg/mL, for 135 mg and 270 mg, respectively.

1.3.2. **Phase 2/3 Study MT-2-01**

This is a phase 2/3, randomized, double-blind, placebo-controlled, multinational, multicenter study with open-label treatment extension to address the effect of MIN-102 on the progression of adrenomyeloneuropathy (AMN) in adult male subjects with X-linked ALD. This study is a 2-part study with enrollment in the U.S. and Europe. Part 1 is the double-blind part of the study that has been completed; Part 2 is the open-label treatment extension that is ongoing. Patient recruitment was closed for this study on November 28th, 2018, with 116 male subjects randomized to MIN-102 or placebo in a 2:1 ratio during the 96-weeks' double-blind treatment period (Part 1). This is an exposure-controlled

study where subjects receive an individualized daily dose to achieve a target exposure (AUC) of 200 µg•hr/mL. Of the 116 randomized patients, 96 patients (82.8%) completed the double-blind treatment period and overall 20 patients (17.2%) prematurely discontinued from treatment; 15 (19.5%) of these patients were in the leriglitazone group, and 5 (12.8%) in the placebo group. The most frequent reasons for premature treatment discontinuation were AEs, reported for 8 patients (10.4%) in the leriglitazone group, and 2 patients (5.1%) in the placebo group, and withdrawal of consent, reported and 6 patients (7.8%) in the leriglitazone group, and for 3 patients (7.7%) in the placebo group. For 1 patient in the leriglitazone group, the reason was reported as "other" without further information.

The primary objective of Part 2 is to assess the safety and tolerability of MIN-102 upon long-term treatment. A total of 87 (90.6%) of 96 completing patients entered extension phase. As of May 16, 2021, 11 patients (12.6%) have discontinued from treatment, of which 7 patients were previously in placebo and 4 in MIN-102 group. The most frequent reasons for treatment discontinuation were AEs, reported for 6 patients (54.5%) previously on the placebo group and 3 patients (27.2%) in the MIN-102 group, and withdrawal of consent, reported for 1 patient (9.0%) previously on the placebo group. For 1 patient previously in the MIN-102 group, the reason was reported as "other". Currently there are 76 patients in the ongoing open-label extension.

1.3.2.1. **Safety**

During Part 1 of the study, one suspected unexpected serious adverse reaction (SUSAR)(SAE) with term "increased hepatic enzyme" was reported that was considered by the investigator to be related to study drug. This event occurred in subject [redacted], a [redacted] male subject as mild in severity and with seriousness criteria of important medical event. The patient was diagnosed during the study with Gilbert's syndrome which can be a cause of increases of liver enzymes; enzyme levels normalized after temporary suspension of study drug, study drug was resumed, and he continued until the end of the double-blind part of the study at a reduced dose without further enzyme elevations and subsequently taking part in the open-label extension study.

No SUSARs have been reported during the open-label treatment extension (Part 2).

As expected, based on the mechanism of action of the compound, weight gain and oedema were the most frequent adverse events associated with MIN-102 treatment. The most frequently reported TEAEs in the MIN-102 group included weight increased (64.9%), peripheral oedema (62.3%), and increased lacrimation (15.6%). Weight increased and oedema peripheral were also among the most frequently reported TEAEs in the placebo group (23.1% and 17.9, respectively). No specific risk factors or at-risk groups for the development of weight gain/oedema have been identified in patients receiving MIN-102.

1.3.2.2. **Efficacy**

[redacted]

[redacted]

1.4. Study Rationale

This study will provide an assessment of the effects of MIN-102 on cALD disease progression prior to HSCT, currently the only approved treatment in this indication, or experimental ex-vivo autologous stem cell transplant. There is currently no treatment available during this phase from first detection of cerebral lesions until actual transplant, and self-arrest is rarely seen. Therefore, this study addresses an unmet medical need.

Cerebral ALD can be detected prior to the development of clinical symptoms via regular surveillance using magnetic resonance imaging (MRI) of the brain. Detection of the first brain MRI abnormalities precedes the onset of significant clinical symptoms by at least 6 months to 1 year (Miller et al., 2016). Several states in the United States (U.S.) and other countries have MRI surveillance programs for boys found to have the ALD genetic marker during newborn screening. It is recommended that boys with the marker have MRIs every 6 months starting at the age of 2 years, and every 3 months after the first occurrence of non-inflammatory lesions (Engelen et al., 2012). Patients showing inflammatory lesions on MRI or progression of overall white matter lesion volume may be shortlisted for allogeneic hematopoietic stem cell transplantation (HSCT) or equivalent procedures and remain under clinical surveillance until then. In the absence of biomarkers to predict disease evolution, brain MRI using gadolinium (Gd) remains the only tool to detect cALD at an early stage, before rapid progression to severe neuropsychiatric symptoms.

The protocol with its schedule of assessments is aligned with this surveillance program and follows standard of care until initiating HSCT procedures or a decision not to undertake HSCT. Patients are eligible for the study at the first occurrence of cerebral MRI abnormalities. Once subjects are identified, either via newborn screening, or during genetic testing, or elevation of VLCFA and confirmed family history by genetic testing of a family member, or having become symptomatic, scheduled MRI and clinical assessments take place at the same timepoints as demanded by the surveillance program. The protocol assesses whether MIN-102 has had an effect on the progression of the disease at two critical timepoints:

1. 24 weeks after inclusion in the study, when historical data suggest that the majority of cerebral lesions has converted from non-inflammatory (without Gd enhancement, Gd-) to inflammatory (Gd-enhancing, Gd+), or if Gd+ at baseline, when in the majority of patients there is rapid growth of lesions and clinical progression, and
2. 96 weeks after inclusion in the study, with a long-term confirmatory assessment on the effect on disease progression.

Hematopoietic stem cell transplantation may be performed at any time point, hence standard of care treatment will not be delayed. The subject will adhere to the visit schedule until initiation of HSCT

procedures. Prior to initiation of HSCT procedures, the subject will be scheduled for another visit irrespective of the time since the Baseline Visit: the "Visit prior HSCT".

The clinical outcome of HSCT is significantly improved if the procedures are carried out before patients develop more severe MRI lesions (Miller et al., 2011; Engelen et al., 2012). More intense pre-transplant Gd enhancement has been shown to predict poorer clinical status by NFS (Miller et al., 2016). In addition, higher MRI severity scores at the time of transplant have been shown to be negatively correlated with better neurocognitive performance post-transplant (Pierpont et al., 2017).

Therefore, there is a high unmet medical need for a treatment that prevents or delays the onset of cerebral inflammatory lesions, and/or halts disease progression.

1.5. Risk benefit assessment

[redacted]

[redacted]

[redacted]

[redacted]

[redacted]

[redacted]

On balance, MIN-102 exhibits favorable efficacy and a safe profile for treatment of cALD, which warrants testing in these patients who currently have no approved pharmacological treatment options during the phase before HSCT.

2. OBJECTIVES

2.1. Primary Objective

The primary objective of the study is to evaluate whether MIN-102 can halt disease progression of cALD at week 96 as determined by serial clinical and magnetic resonance imaging (MRI) investigations in pediatric subjects.

2.2. Secondary Objectives

The secondary objectives of the study are as follows:

- To assess the changes in neurological function.
- To evaluate the effects of pre-HSCT MIN-102 treatment on:
  - Loes scores
  - Gadolinium intensity score (GIS)
  - Overall survival of patients who have not undergone HSCT
  - Number of patients meeting HSCT criteria
- To assess the pharmacokinetics (PK), safety, tolerability, and palatability of MIN-102 in pediatric subjects

2.3. Exploratory Objectives

To evaluate the effects of MIN-102 treatment on:

- White matter T2/FLAIR hyperintensity lesion volume
- White matter fiber structure as determined by diffusion tensor imaging (DTI)
- Cerebral blood flow, blood volume, capillary mean transient time heterogeneity (CHT) and constants of permeability (K2 and Kapp) as determined by DSC MRI
- Plasma biomarkers, and cerebrospinal fluid (CSF) biomarkers (optional)

Study endpoints are defined in sections 3.6.1.3, 3.6.1.4, 3.6.1.5 and 3.6.1.6.

3. INVESTIGATIONAL PLAN

3.1. Overall Study Design and Plan

This is an open-label, phase 2, multicenter study in male pediatric subjects, aged ≥2 and ≤12 years, with a diagnosis of the cALD phenotype of X-linked ALD. After written informed consent by the parent/legal guardian, or authorized legal representative and completed assent as appropriate, is obtained, and after completion of all screening evaluations with all inclusion criteria and exclusion criteria satisfied, subjects will be enrolled to be treated with MIN-102. Study subjects enrolled may present different MRI status prior to first dose of study drug: subjects without Gd-enhancing cerebral lesions (referred to as 'Population 1' in this protocol) and subjects presenting Gd-enhancing lesions (Population 2). It is anticipated that approximately 13 subjects will be included in the study. Additional subjects may be enrolled after agreement with the Sponsor to ensure that the study recruits 13 evaluable subjects who receive MIN-102 treatment. Decisions regarding subject replacement will be documented. Patients who meet HSCT criteria and patients who at week 96 do not meet "arrested disease" criteria will not be replaced. However, patients who drop out by withdrawal of consent by parents for non-treatment related causes may be replaced. Patients dropping out will be encouraged to remain in the study and attend all further scheduled efficacy and safety assessments. Decisions regarding subject replacement will be documented.

The starting dose will be established per body weight in each subject, as determined by PBPK modeling to achieve an exposure of approximately 170 µg•hr/mL and then re-adjusted, if needed to this target exposure. Subjects will take one dose of MIN-102 at approximately the same time each morning, administered orally with a 5-mL syringe.

Each subject will undergo screening for a maximum of 15 days, followed by a treatment period. If the screening procedures are performed within 7 days prior to Baseline (V0), all results obtained from the screening evaluations will serve as the Baseline values and pre-dose Baseline procedures will only consist of the clinical efficacy assessment (NFS-MFD), the blood sampling for biomarkers and MIN-102 plasma levels, and the optional CSF sampling for biomarkers. Subjects will be evaluated at the Screening Visit, at the Baseline Visit when the first dose of study drug is administered, and at regular biweekly intervals thereafter until the Week 12 (Visit 6). After Visit 6, evaluations will occur at Week 18 (Visit 7), Week 24 (Visit 8), Week 36 (Visit 9), Week 48 (Visit 10), Week 72 (Visit 11), Week 96 (Visit 12), and at further 24-week intervals thereafter until end-of-treatment criteria apply (Table 1).

Hematopoietic stem cell transplantation may be performed at any time point after the Baseline Visit. The subject will adhere to the visit schedule until initiation of HSCT procedures. Prior to initiation of HSCT procedures, the subject will be scheduled for another visit irrespective of the time since the Baseline Visit: the "Visit prior HSCT". Last dose of study drug will be administered one day prior to this visit. This visit is the final visit in the study for patients undergoing HSCT.

Patients meeting the following criteria will be scheduled for HSCT ("HSCT criteria"):

- GIS of 1, 2 or 3, or
- Significant T2/FLAIR lesion growth as assessed by central reading, present at two consecutive MRIs at least 12 weeks apart from each other

If HSCT criteria are met at the first visit, but not at the second visit, another visit will be scheduled 12 weeks after the second visit to confirm presence or absence of criteria for HSCT. If patients meet HSCT

criteria for the first time at Visit 10 (Week 48) or later, an unscheduled visit will occur 12 weeks (±15 days) afterwards. If HSCT criteria are not met at the unscheduled visit, the subject will continue with the regular visit schedule. If HSCT criteria are met at two consecutive visits, the subject will be scheduled for HSCT.

The investigator may schedule the ”Visit Prior to HSCT” as close as up to 5 days prior to initiation of HSCT procedures, defined as the day of first administration of myeloablative medication. To lessen the burden for the subject, it is at the investigator's discretion to cancel a visit within the regular schedule of visits, should such a visit be within 4 weeks from the "Visit Prior to HSCT". Treatment with MIN-102 will be discontinued with the last dose of study drug administered on the day immediately before the "Visit Prior to HSCT".

If the subject is shortlisted for HSCT, but meets contraindications against HSCT, or HSCT is not performed due to unavailability of a donor, treatment with study drug may continue if decided by the PI. If treatment is stopped, all assessments equivalent to the "Visit Prior to HSCT" will be performed one day after last dose of study drug. The Follow-up Visit will be scheduled 4 weeks (±5 days) after last dose of study drug.

If a subject drops out of the study before a planned HSCT, the Follow-up Visit should be performed 28 days after last administration of study drug, or immediately before initiation of HSCT procedures if these occur earlier than 28 days after last dose of study drug. Subjects who stay in the study and undergo HSCT will not have a Follow-up Visit.

Figure 1 depicts the overall study design, and Table 1 presents a flow chart of study procedures. Section 3.1.1 describes the study evaluations by visit.

3.1.1. **Visits and Assessments**

During the first 12 weeks of treatment (until V6), safety assessments will be performed every 2 weeks. To reduce the burden of travel for subjects, an experienced certified nurse trained in Good Clinical Practice (GCP) may conduct the following visits in the subject's home: Visits 1, 3, 4, 5 and 7. The investigator will propose subjects eligible for home visits to the sponsor for approval. Criteria for conducting home visits will be the distance of the subject's home from the study site, time and burden required for travel, and the subject's clinical condition. Visit 2 and all visits from Visit 6 onwards (except Visit 7) will be on-site visits.

3.1.1.1. **Screening Visit (V-1)**

The screening phase must be completed within 15 days prior to the Baseline Visit and should be kept as short as possible. The Screening Visit will consist of the following assessments and procedures:

1. Subject informed consent. The subject's parent/legal guardian or authorized legal representative will sign the consent form prior to any study-specific screening procedures. The signed consent form will be retained and archived at the study site, and a copy will be provided to the subject's parent/legal guardian. An assent form will be completed with the subject as applicable.
2. Assignment of subject identification number
3. Review of inclusion/exclusion criteria
4. Recording of demographics data

5. Medical history/concomitant disease review
6. Prior medication review
7. Assessment of vital signs (body weight, height, blood pressure, pulse rate, and temperature)
8. Physical examination. Findings will be reported as medical history or concomitant disease
9. 12-lead ECG recorded in triplicate
10. Echocardiogram
11. Laboratory safety tests (see Section 3.5.3.2 or Table 8)
12. Cerebral MRI: performed after confirmation that all other inclusion criteria and none of the exclusion criteria are met. If the MRI cannot be scheduled during the Screening Visit, it will be performed at the Baseline visit (V0) prior to first dose of study medication.
    Note: If a valid MRI obtained with the same study-specific specifications and obtained within 15 days before Screening is available, this will be considered the Screening MRI and no extra MRI will be performed.

After completion of all screening evaluations, and provided that all inclusion criteria and no exclusion criteria are met, the Baseline Visit will be scheduled.

3.1.1.2. **Baseline Visit (V0)**

After confirming that all inclusion and no exclusion criteria are fulfilled, subjects will receive the first dose of study drug on-site. No separate baseline procedures are to be performed for those procedures that were already performed at the Screening Visit (V -1). All results obtained at the Screening Visit (V -1) will serve as the baseline values.

The Baseline Visit will consist of the following assessments and procedures:

1. Confirmation of inclusion and exclusion criteria
2. Update of medical history
3. Update of previous medications
4. Pre-dose 12-lead ECG recorded in triplicate
5. Laboratory safety tests (see Section 3.5.3.2 or Table 8)
6. Cerebral MRI (if not performed at the Screening Visit)
7. Clinical efficacy assessment: NFS-MFD
8. Optional CSF sampling via lumbar puncture to assess biomarkers
9. Blood sampling for biomarkers and PK.
10. Study drug dispensation
11. On-site administration of first dose of MIN-102
12. Palatability assessment (immediately after MIN-102 administration)
13. Blood spot glucose check 2 hours (±0.5 hours) after MIN-102 administration

14. 12-lead ECG recorded in triplicate 2 hours (± 0.5 hours) after MN-102 administration
15. Blood sampling for MIN-102 levels 2, 6, 12, and 24 hours after MIN-102 administration (requiring an overnight stay).
16. AE recording
17. Record of concomitant medication

3.1.1.3. Visit 1 (at Week 2, V1, possible home visit)

The Visit 1 will consist of the following assessments and procedures:

1. Vital signs (body weight, blood pressure, pulse rate, and temperature)
2. Laboratory safety tests (see Section 3.5.3.2 or Table 8)
3. Examination for peripheral edema
4. Administration of daily dose of MIN-102
5. Blood spot glucose check
6. AE recording
7. Record of concomitant medication review

3.1.1.4. Visit 2 (at Week 4, V2)

The Visit 2 will consist of the following assessments and procedures:

1. Vital signs (body weight, height, blood pressure, pulse rate, and temperature)
2. Physical examination
3. Cerebral MRI (optional)
4. Laboratory safety tests (see Section 3.5.3.2 or Table 8)
5. Urinalysis and urine cytology
6. Subject diary review
7. Study drug accountability
8. Blood sampling for biomarkers (prior to MIN-102 administration)
9. Blood sampling for MIN-102 level (immediately before MIN-102 administration)
10. On-site administration of daily dose of MIN-102
11. Palatability assessment (immediately after MIN-102 administration)
12. 12-lead ECG recorded in triplicate 2 hours (± 0.5 hours) after MIN-102 administration
13. Blood spot glucose check 2 hours (±0.5 hours) after MIN-102 administration)
14. Blood sampling for MIN-102 levels 2 hours after MIN-102 administration
15. AE recording

16. Record of concomitant medication

3.1.1.5. **Visit 3 (at Week 6, V3; possible home visit)**

The Visit 3 will consist of the same assessments and procedures performed at the Visit 1 (section 3.1.1.3).

3.1.1.6. **Visit 4 (at Week 8, V4; possible home visit)**

The Visit 4 will consist of the same assessments and procedures performed at the Visit 1 (section 3.1.1.3), with the addition of 12-lead ECG, recorded in triplicate 2 hours (± 0.5 hours) of MIN-102 administration.

3.1.1.7. **Visit 5 (at Week 10, V5; possible home visit)**

The Visit 5 will consist of the same assessments and procedures performed at the Visit 1 (section 3.1.1.3).

3.1.1.8. **Visit 6 (at Week 12, V6)**

The Visit 6 will consist of the following assessments and procedures:

1. Vital signs (body weight, height, blood pressure, pulse rate, and temperature)
2. Physical examination
3. 12-lead ECG recorded in triplicate 2 hours (± 0.5 hours) after MIN-102 administration)
4. Clinical efficacy assessment: NFS-MFD
5. Cerebral MRI
6. Optional CSF sampling via lumbar puncture to assess biomarkers
7. Laboratory safety tests (see Section 3.5.3.2 or Table 8)
8. Urinalysis and urine cytology
9. Subject diary review
10. Study drug accountability
11. Blood sampling for biomarkers (prior to MIN-102 administration)
12. Blood sampling for MIN-102 level (prior to MIN-102 administration)
13. Study drug dispensation
14. On-site administration of daily dose of MIN-102
15. Palatability assessment (immediately after MIN-102 administration)
16. Blood spot glucose check 2 hours (±0.5 hours) after MIN-102 administration
17. Blood sampling for MIN-102 levels 2 hours after MIN-102 administration
18. AE recording
19. Record of concomitant medication

3.1.1.9. Visit 7 (at Week 18, V7; possible home visit)

The Visit 7 will consist of the same assessments and procedures performed at the Visit 1 (section 3.1.1.3).

3.1.1.10. Visit 8 (at Week 24, V8) and Subsequent Regular Visits (V9, V10 andV11) (at Week 36, 48 and 72 respectively)

The Visit 8 and any subsequent regular visits will consist of the following assessments and procedures:

1. Vital signs (body weight, height, blood pressure, pulse rate, and temperature)
2. Physical examination
3. 12-lead ECG recorded in triplicate 2 hours (± 0.5 hours) after MIN-102 administration
4. Clinical efficacy assessment: NFS-MFD
5. Cerebral MRI
6. Laboratory safety tests (see Section 3.5.3.2 or Table 8)
7. Urinalysis and urine cytology
8. Subject diary review
9. Study drug accountability
10. Blood sampling for biomarkers (prior to MIN-102 administration)
11. Blood sampling for MIN-102 level (immediately before MIN-102 administration)
12. Dispensation of study drug
13. On-site administration of daily dose of MIN-102
14. Palatability assessment (immediately after MIN-102 administration)
15. Blood spot glucose check 2 hours (±0.5 hours) after MIN-102 administration
16. Study drug accountability
17. AE recording
18. Record of concomitant medication

3.1.1.11. Visit 12 (at Week 96, V12)

The Visit 12 will consist of the following assessments and procedures:

1. Vital signs (body weight, height, blood pressure, pulse rate, and temperature)
2. Physical examination
3. 12-lead ECG recorded in triplicate 2 hours (± 0.5 hours) after MIN-102 administration
4. Clinical efficacy assessment: NFS-MFD
5. Cerebral MRI
6. Optional CSF sampling via lumbar puncture to assess biomarkers

7. Laboratory safety tests (see Section 3.5.3.2 or Table 8)
8. Urinalysis and urine cytology
9. Subject diary review
10. Study drug accountability
11. Blood sampling for biomarkers (prior to MIN-102 administration)
12. Blood sampling for MIN-102 level (immediately before MIN-102 administration)
13. Dispensation of study drug
14. On-site administration of daily dose of MIN-102
15. Palatability assessment (immediately after MIN-102 administration)
16. Blood spot glucose check 2 hours (±0.5 hours) after MIN-102 administration
17. Study drug accountability
18. AE recording
19. Record of concomitant medication

Regular visits will continue to be scheduled at 24-week intervals until end-of-treatment criteria are met and will consist of the same assessments as Visit 12 above, except optional CSF sampling. Once end-of-treatment criteria are met, Follow-up visit will be scheduled.

3.1.1.12. **Visit Prior to HSCT**

Hematopoietic stem cell transplantation may be performed at any time point after the Baseline Visit provided the patient has met "HSCT criteria" or that overall lesion progression in the assessment of the investigator mandates immediate scheduling of HSCT. Prior to initiation of HSCT procedures, and irrespective of the time from the Baseline Visit, this visit will be scheduled. Should this "Visit prior to HSCT" be scheduled within 4 weeks to another study visit, the Investigator may decide to omit this other study visit and perform only the "Visit prior to HSCT" in its place. The "Visit Prior to HSCT" will consist of the following assessments and procedures:

1. Vital signs (body weight, height, blood pressure, pulse rate, and temperature)
2. Physical examination
3. 12-lead ECG recorded in triplicate
4. Clinical efficacy assessment: NFS-MFD
5. Cerebral MRI
6. Optional CSF sampling via lumbar puncture to assess biomarkers
7. Laboratory safety tests (see Section 3.5.3.2 or Table 8)
8. Subject diary review
9. Study drug accountability
10. Blood sampling for biomarkers

11. Blood sampling for MIN-102 level
12. Blood spot glucose check
13. AE recording
14. Record of concomitant medication

Treatment with MIN 102 will be discontinued at **least five days** prior to initiating HSCT procedures, defined as the day of first administration of myeloablative medication. The last dose of study drug will be administered on the day before this visit. This visit is the final visit in the study for subjects undergoing HSCT.

3.1.1.13. Follow-up Visit

Subjects who either drop out of the study before HSCT procedures are initiated or discontinue MIN-102 after the decision not to undergo HSCT will return, if possible and as far as their general health status permits, for a Follow-up Visit 28 (± 5) days after last administration of MIN-102. If a subject drops out of the study before planned HSCT, the Follow-up Visit should be performed 28 days after last administration of study drug, or immediately before initiation of HSCT procedures if these occur earlier than 28 days after last dose of study drug. The Follow-up Visit will consist of the following assessments and procedures:

1. Vital signs (body weight, height, blood pressure, pulse rate, and temperature)
2. Physical examination
3. 12-lead ECG recorded in triplicate
4. Clinical efficacy assessment: NFS-MFD
5. Laboratory safety tests (see Section 3.5.3.2 or Table 8)
6. AE recording
7. Record of current medication

Subjects who stay in the study and undergo HSCT will not have a Follow-up Visit.

3.1.1.14. Unscheduled Visits

Additional (unscheduled) visits during the study may also be performed as necessary in case of any concerns e.g. to follow up on any adverse event, or to follow up on MRI findings. Unscheduled visits will be recorded in the eCRF. In case any unscheduled laboratory tests are necessary during or after the study completion, these should be performed by the centralized companies.

3.1.2. Study termination

3.1.2.1. End-of-treatment criteria

Subjects will receive treatment until the first occurrence of any of the following end-of-treatment criteria:

1. Subject undergoes HSCT, having met HSCT criteria (see section 3.1.)
2. Subject undergoes HSCT, if investigators' overall assessment of lesion progression mandates immediate scheduling of HSCT although "HSCT criteria" are not met.

   Subjects who undergo HSCT according to criteria 1 and 2 above may receive study drug until the day before the last study visit prior to initiating HSCT procedures ("Visit Prior to HSCT"). This visit will occur a minimum of 5 days before first administration of myeloablative medication.
3. Study fails to meet "continuation criteria" at Week 24.
4. Subject fails to meet primary endpoint of "arrested disease" at Week 96.
5. The final evaluation of the study is performed at Week 96 and shows that less than 4 of 13 enrolled patients meet criteria for "arrested disease".
6. The subject's parent/legal guardian, or authorized legal representative withdraws consent.
7. In the clinical judgment of the investigator, the subject's general health status declines to an extent that the risks of treatment outweigh the potential benefits.
8. ***Subjects do not tolerate the minimum allowed dose (see section 3.1.2)***
9. ***Subjects show clinically significant out-of-range laboratory values, clinically significant abnormal findings on physical examination, or intolerable adverse events (AEs) that put him at additional risk, as judged by the investigator. For termination of treatment in case of drug induced liver injury (DILI) refer to section 6.2.***[1]
10. MIN-102 is commercially available for the treatment of cALD.

3.1.2.2. Removal of Subjects from the study

Participation in the study is strictly voluntary. Parents, the legal guardian or authorized legal representative may withdraw consent and discontinue their child from the study for any reason at any time.

If a subject is withdrawn from the study, the study monitor will be informed immediately. If there is a medical safety reason for withdrawal, the subject will remain under the supervision of the investigator or the subject's local health care center until satisfactory health has returned.

In case steady elevations of NT-proBNP >300 pg/mL occur, the patient will undergo exploration of clinical symptoms indicative of heart failure, a physical examination and an echocardiogram including the suggested examinations. Treatment with study drug may be suspended during these investigations. If these examinations reveal a clinically significant risk of heart failure by judgment of the investigator and cardiologist, the patient will be excluded from the study. Otherwise, if physical examination shows no signs of heart failure along with preserved LVEF% (>50%), study drug, if suspended, may be resumed by judgment of the investigator and administration of diuretics must be considered. After

[1] ***Only applicable in France according to the current approved protocol version 6.2***

resumption of study drug and administration of diuretics (if no contraindications), unscheduled laboratory assessments of NT-proBNP, clinical examinations and an echocardiogram with evaluations such as myocardial deformation (strain) assessed by Tissue Doppler Imaging (TDI) or on bidimensional images (speckle tracking) should be considered as appropriate. If a risk of heart failure cannot be excluded in the clinical judgment of the investigator and cardiologist, the subject will be permanently discontinued.

3.1.2.3. Study completion

The study will be considered to be completed:

- When up to 13 evaluable subjects have performed Visit 12 (V12), or "Visit Prior to Transplant".
- If a minimum of 4 of 13 evaluable subjects have met the definitions of "arrested disease" at V12. However, subjects who are beyond V12 may continue in the study until end-of-treatment criteria are reached.

In addition, in the following cases the entire study will be terminated:

- If the study fails to meet the primary endpoint of "arrested disease" at Week 96.
- Study fails to meet "continuation criteria" at Week 24.
- If more than 9 subjects met the predefined "HSCT criteria" irrespective of whether they perform "Visit Prior to Transplant"
- In case of an unfavorable risk/benefit ratio due to occurrence of significant safety risk.

The sponsor (Minoryx Therapeutics S.L.) reserves the right to discontinue the study, a study site, or multiple study sites for safety or administrative reasons at any time. Should the study be terminated, and/or a site closed for whatever reason, all documentation, clinical supplies, and study drug must be returned to the sponsor or its representative.

3.1.3. Data Safety Monitoring Board

An independent Data and Safety Monitoring Board (DSMB) will receive each subject's data after he has reached V8 at 6 months, or completed treatment with MIN-102 prior to HSCT (if earlier), or has completed the Follow Up Visit. The DSMB may also review subject data ad hoc, as needed. The DSMB can recommend stopping or modifying the study at any time if unacceptable safety risks become apparent or the number of dropouts is much higher than anticipated. The reasons for dropouts will be carefully investigated. The DSMB can also recommend a downward adjustment of the MIN-102 target exposure range if safety risks are identified. The composition and function of the DSMB has been established by a separate charter.

3.2. Discussion of Study Design

Sections 3.6.1.3, 3.6.1.4, 3.6.1.5 and 3.6.1.6 discuss the rationale for the study endpoints, and section 3.4.3 discusses the rationale for MIN-102 dose selection. Because cALD is a life-threatening rare disease with no accepted treatments other than HSCT, the study design is an open-label design with no comparator.

A comparison of study results will be performed with natural history data available in the scientific literature on the rates of self-arrest (Moser et al., 2000; Engelen et al., 2012, Eichler, 2016; Raymond et al., 2019; Liberato et al., 2019).

A comparison of MRI study results will also be performed with a separate sample of patients who received HSCT between the years 2007 and 2016. This is a sample of 13 patients derived from the database at the University of Minnesota. Criteria for the selection of these patients were:

- Age between 2 and 12 years, inclusive
- Two MRI scans (V1 and V2) before and 1 scan after HSCT (V3)
- HSCT ≤1.5 years after first MRI
  - A subpopulation of 8 patients was evaluated separately who fulfilled all above criteria but had HSCT performed ≤0.5 years since first MRI.

The following MRI parameters were evaluated in this sample:

1. FLAIR based overall lesion burden volume
2. Volume of inflammatory (Gd enhancing) lesions
3. Percent-volume of the total FLAIR based volume

Due to technical limitations of the archived data that were made accessible to Minoryx, the full set of parameters could not be evaluated in all patients.

The objective of this investigation was to confirm that there is lesion evolution in this group of patients who did not receive treatment until HSCT, and that HSCT as the only approved treatment in this indication is able to positively influence lesion evolution.

Table 2 shows the percent change from first to second pre-HSCT scan (V1 to V2) and from the second scan before HSCT (V2) to the scan after HSCT (V3).

Table 2. Percent Change in Lesion FLAIR Over Time per-HSCT and After HSCT From University of Minnesota Database

Parameter	Evaluated population (n=13)		Subpopulation[1] (n=8)	
	% change V1 – V2	% change V2 – V3	% change V1 – V2	% change V2 – V3
**FLAIR overall lesion volume**	291	33	69	-15
**Volume Gd-enhancing lesión[2]**	192	-64	357	-68
**Volume Gd-enhancing lesion as % of total FLAIR volume***	39	-36	56	-19

FLAIR = fluid attenuation inversion recovery; Gd = gadolinium

[1] The evaluated population had HSCT ≤ 1.5 years after first MRI. A subpopulation of 8 patients was evaluated separately who had HSCT performed ≤0.5 years since first MRI.

[2] n=10 in eligible population, n=7 in subpopulation

These changes demonstrate that there is lesion evolution in untreated subjects within the timeframe of MRI investigations prior to transplant, and stabilization or reduction of lesion evolution after HSCT. Overall lesion volume by FLAIR and volume of the inflammatory (Gd-enhancing) lesions show marked increases prior to transplant and stabilization or reduction after HSCT. As well, before HSCT the volume of the Gd-enhancing lesion increases more than the overall volume increase of lesion per FLAIR, and recesses to larger extent than overall FLAIR lesion volume after HSCT.

These findings confirm the choice of the primary MRI parameters for this study. Evaluation of the same MRI parameters in this study is possible and will allow an assessment of the evolution of these parameters with treatment with MIN-102 until HSCT. In addition to comparisons from Baseline (V0) to immediately prior to transplant using each patient as his own control, comparisons between these parameters and those obtained from the external cohort will support an exploratory assessment whether MIN-102 is able to influence the evolution of cerebral lesions prior to transplant.

3.3. Selection of Study Population

Approx. 13 male subjects will be included in the study. Additional subjects may be enrolled after agreement with the Sponsor to ensure that the study recruits 13 evaluable subjects who receive MIN-102 treatment. Decisions regarding subject replacement will be documented. Patients who meet HSCT criteria and patients who at week 96 do not meet “arrested disease” criteria will not be replaced. However, patients who drop out by withdrawal of consent by parents for non-treatment related causes may be replaced. Patients stopping treatment will be encouraged to remain in the study and attend all further scheduled efficacy and safety assessments.

3.3.1. Inclusion Criteria

Only subjects who meet all following criteria will be eligible for inclusion in the study:

1. Written informed consent by parent/legal guardian, or authorized legal representative to participate in the study
2. Males aged ≥2 and ≤12 years with diagnosis of X-linked ALD based on genetic testing; or, in absence of genetic testing, elevation of VLCFA and confirmed by family history of X-ALD with clinical symptoms and elevation of VLCFA or by genetic testing of a family member
3. White matter involvement as determined by cerebral MRI lesions without Gd enhancement at baseline (Population 1), or with Gd enhancement at baseline (Population 2)
4. Major Functional Disabilities (MFD) score of 0, as determined by key measures in the Neurological Function Score (NFS)
5. Baseline Loes score >0 and ≤10
6. Baseline GIS ≤3
7. No signs or symptoms of adrenal insufficiency and morning cortisol and aldosterone levels within normal laboratory ranges for age, or appropriate steroid replacement if adrenal insufficiency is present. A history of adrenal insufficiency is not exclusionary if the foregoing criteria are currently met.
8. Glycated hemoglobin (HbA1c) within normal range

3.3.2. Exclusion Criteria

Subjects will be excluded from the study if they meet any of the following criteria:

1. Other chronic neurological disease
2. Known intolerance to pioglitazone or other thiazolidinediones
3. Use of pioglitazone or other thiazolidinediones within the past 6 months prior to screening
4. Use of biotin at a daily dose of >50 mg per day within the past 3 months prior to screening
5. Current participation in another interventional clinical study, or participation in such a study within 6 months prior to screening
6. Previous HSCT
7. Requirement for a prohibited concomitant medication (section 3.4.10)
8. Previous or current history of bladder polyps, bladder cell hyperplasia, or cancer (other than successfully treated basal cell carcinoma)
9. ***Chronic or recurrent symptomatic urinary infections (≥2 per year over the past 2 years until Screening [V-1])[1]***
10. ***Permanent indwelling urinary catheter or catheter port[1]***
11. ***Smoking with 25 cigarettes per day over the past 2 years until Screening (V-1)[1]***
12. Previous or current history of congestive heart failure
13. Clinically significant anemia with hemoglobin <10 g/dL
14. Alanine aminotransferase (ALT) or aspartate aminotransferase (AST) level >2 times the upper limit of normal (ULN) or total bilirubin >1.5 times the ULN (unless due to Gilbert's syndrome)
15. Moderate or severe hepatic impairment (groups B and C according to Child-Pugh classification)
16. eGFR < 90 ml/min or any evidence of renal disease or impairment, including proteinuria or hematuria
17. Pulmonary disease or cardiac disease of sufficient severity to limit participation in the study and/or completion of study procedures
18. Reduced left-ventricular ejection fraction or other clinically significant cardiac abnormalities on echocardiogram that, in the investigator's opinion, could predispose the subject to volume overload or its attendant consequences
19. Hereditary Fructose Intolerance
20. History of diabetes, or glycated hemoglobin (HbA1c) levels >6.4% and fasting blood glucose levels ≤ 0.9 times the lower limit of normal and ≥ 1.1 times the upper limit of normal at Screening

[1]***Only applicable in France according to the current approved protocol version 6.2***

21. ***A positive result on laboratory tests for hepatitis B surface antigen, hepatitis C antibody or human immunodeficiency virus antibody***[1]
22. Contraindication to MRI procedure, such as presence of ferromagnetic materials (aneurysm clips, pacemaker, intraocular metal, cochlear implant) in the body
23. Conditions that could modify the absorption of the study drug
24. Inability or unwillingness of parent/legal guardian or subject to comply with the study procedures as per investigator's criteria.
25. Inability or unwillingness of parent/legal guardian or subject to resume standard of care at a local center once study is complete or criteria for HSCT is met and treatment available.
26. Other medical, neurologic, psychiatric, or social condition that, in the opinion of the investigator, is likely to unfavorably alter risk-benefit of study participation, confound interpretation of safety or efficacy results, or interfere with the satisfactory completion of study requirements.

3.4. Treatment

3.4.1. Treatments Administered

All subjects will receive MIN-102 (open label). No randomization or blinding will be used in this study.

3.4.2. Identity of Investigational Product

The active substance in MIN-102 is a small molecule differentiated peroxisome proliferator-activated receptor (PPAR) γ agonist. The active drug substance is a metabolite of pioglitazone, an approved treatment for type 2 diabetes.

Active substance:	[redacted]
INN:	leriglitazone
Activity:	PPARγ agonist
In development for:	ALD
Strength:	[redacted]
Dosage form:	Oral suspension
Posology:	Once-daily dosing with a volume specified by the pharmacokinetic specialist to achieve the target plasma exposure (170 µg·hr·mL^{-1})
Manufacturer:	Laboratorium Sanitatis SL

---

[1] ***Only applicable in France according to the current approved protocol version 6.2.***

Table 3. Composition of Study Medication

[redacted]	[redacted]
[redacted]	[redacted]
[redacted]	[redacted]
[redacted]	[redacted]
[redacted]	[redacted]
[redacted]	[redacted]
[redacted]	[redacted]
[redacted]	[redacted]
[redacted]	[redacted]
[redacted]	[redacted]
[redacted]	[redacted]

The study drug is supplied as an oral aqueous-based suspension and is administered orally once daily (1 mL of oral suspension contains 13.66 mg of leriglitazone, 15 mg of leriglitazone hydrochloride). Company code MIN-102 is used to refer to the salt of leriglitazone (as hydrochloride) when administered, however, when reporting plasma or cerebrospinal fluid (CSF) concentrations/exposure MIN-102 refers to the free base (leriglitazone).

3.4.3. Selection of Dose and Dose Adjustment Criteria

The target dose and exposure in humans are based on the PK findings of the phase 1 study MT-1-01 in healthy adult males and on PBPK modeling, and supported by calculations of MIN-102 exposure with respect to pharmacodynamic effects in preclinical pharmacology. Dose response based on gene expression was determined in *Abcd1* knockout mice. Dose response based on disability scores was established in *Abcd1*/*Abcd2* double-knockout mice serving as an experimental model of autoimmune encephalomyelitis, and in rats serving as an experimental model of autoimmune neuritis. These results consistently confirmed efficacious doses of 50 mg/kg in mice and 18 mg/kg in rats which correspond to exposure between 100 to 130 µg•hr/mL.

A PBPK model for MIN-102 (Simcyp MIN-102 Final Report 19-Oct, 2018) that incorporates CYP3A4 and CYP2C8-mediated metabolism as well as biliary clearance derived from in-vitro data was developed by Certara using the Simcyp software. During model development, the single-ascending dose (SAD) data were used as the model training dataset and the multiple-ascending dose (MAD) data were used as the model verification dataset. The findings show that CYP induction was low and insignificant. The final MIN-102 model was prospectively applied to estimate an appropriate starting dose for pediatric clinical evaluation. The default Simcyp ontogeny functions for CYP3A4 and CYP2C8 as well as literature ontogeny functions (Upreti and Wahlstrom 2016) were applied in separate simulations. Allometric scaling was also applied to scale doses based on body size and compared to the PBPK derived doses. Since application of the Simcyp default ontogeny versus the Upreti ontogeny functions did not show significant difference in the projected paediatric doses, the Upreti ontogeny were selected as more appropriate for the starting doses. An individualized starting dose based on PBPK will be chosen to yield a geometric mean AUC_t of approximately 170 µg•hr/mL with an expected standard deviation of approximately 20%.

Table 4 shows the starting doses for 3 age ranges.

Table 4. Starting Doses MIN-102

Age Range (years)	Dose (mg/kg per day)	Dose (mL/kg per day)
2 – 5	2.6	0.17
≥ 6 – 11	2.4	0.16
12	2.2	0.15

Blood sampling to determine MIN-102 plasma concentrations will occur at the Baseline Visit (V0) at the following timepoints: pre-dose, 2, 6, 12, and 24 h post-dose. The plasma concentration data of these samples will be reviewed by an experienced pharmacokineticist who will predict steady state exposure from these data and make dose recommendations to achieve the target plasma exposure of 170µg•hr/mL. Subjects should remain at the starting dose until the Investigator receives the PK expert recommendation.

The model will be updated and adjusted, if needed, with PK results obtained during the ongoing study. Additional blood samples to determine MIN-102 plasma concentration will be taken during the study at all on-site visits. Further dose adjustments to achieve the target AUC of 170 µg•hr/mL ± 20% may be made at any time during the study if deemed necessary.

3.4.4. Dose Reductions for Safety or Tolerability Reasons

Individual downward adjustments of the dose at any time point after the Baseline Visit may be implemented by the investigator for safety/tolerability reasons; however, the minimum permitted dose in terms of corresponding volume rounded to the nearest 0.1 mL must achieve a plasma exposure of ≥100 µg•hr/mL.

3.4.4.1. Dose Reductions Before First PK Results

It is highly advisable not to perform dose reductions until the first PK expert's recommendation. In case a dose reduction is unavoidable in the judgement of the Investigator for safety/tolerability reasons before the first PK expert's recommendation, this reduction should be less than 20% from the starting dose, if possible, to prevent subjects from falling below the minimum efficacious exposure. All percentages of dose reduction will be calculated in terms of the corresponding volume rounded to the nearest 0.1 mL.

3.4.4.2. Dose Reductions After PK Expert's Recommendation

If subjects experience safety and/or tolerability issues after a dose recommendation by the PK expert and the investigator determines that a dose reduction is necessary, the dose may be reduced by up to 20% of the most recent dose recommended by the PK expert. If an additional dose reduction is required to control safety/tolerability issues, the Investigator should discuss the amount of the reduction with the Sponsor. In this case, the maximum allowed dose reduction may be up to 40% of the dose recommended by the PK expert. The percent of dose reduction will be calculated in terms of the corresponding volume rounded to the nearest 0.1 mL.

In case the PK expert recommends a dose higher than the dose previously taken by the subject following a dose reduction for safety/tolerability reasons, it is at the discretion of the Principal Investigator to increase the dose or keep it unchanged.

If AEs resolve after a dose reduction, the dose may be increased again, but in no case above the dose that was most recently recommended by the PK expert.

Subjects not tolerating study drug despite the maximum allowed dose reduction will be discontinued from the study.

For those subjects who experience safety/tolerability issues, additional pharmacokinetic analyses may be performed.

3.4.5. **Study Drug Administration and Instructions for Subjects.**

Subjects will take one daily dose of MIN-102 at approximately the same time each morning. Drug will be administered using a 5-mL syringe. Subjects and/or parents/caregivers will be instructed to fill the syringe to the appropriate volume, which will be rounded to the nearest 0.1 mL. After each use, the syringe should be washed with clean water and dried externally with paper until no remains of the study drug suspension are visible on the outer shell of the syringe. A paper diary with instructions for taking study drug will be provided to the subject's parents, legal guardian or authorized legal representative for them to record study drug dosing.

The starting dose of study drug administered will be the dose that achieves a plasma exposure of 170 µg•hr/mL and will then be adjusted in the following visits to this target exposure if needed as described in section 3.4.3.

If a subject misses a dose, the regular, once-daily dosing schedule will be resumed on the day immediately following the day when the dose was missed. Missing doses must not be substituted. The day when a dose was missed will be recorded in the diary.

3.4.6. **Study drug interruptions**

Administration of study drug may be temporarily interrupted in case a subject experiences safety and/or tolerability issues or other conditions that prevent him from taking study drug. Interruptions should be kept to a minimum.

3.4.7. **Treatment Compliance and Study Drug Accountability**

The study drug manufacturer will provide study drug to the sites on a per-subject basis. The study site staff will dispense study drug to the subject as detailed in Table 1. Each dispensation will cover the time period until next visit, including a reserve amount. The investigator is responsible for proper documentation of receipt, storage, and disposition of study drug supplies. Subjects and parents/caregivers will be instructed to return empty and partly used bottles to the study site to assess compliance with the dosing schedule.

The first dose of study drug will be administered at the study site, with a demonstration of the use of the syringe and bottle by study staff. The subject will take subsequent doses at home. On the days of blood sampling to determine MIN-102 plasma concentration and biomarkers, subjects will be instructed not to take their study drug before the study visit; study drug will be taken after blood sampling as

directed by the investigator or study staff. Subjects will be provided with a study diary to record the date, time, and volume of study drug administered each day.

Compliance will be determined at the study site at each visit after the Baseline Visit, when subjects return all previously used and unused bottles. The number of empty bottles will be determined, and the amount as volume in mL of suspension left in returned bottles will be assessed. Compliance with study drug will be calculated as the actual volume in milliliters taken divided by the scheduled volume in milliliters as prescribed by the investigator, expressed as a percentage.

Compliance will also be assessed by determination of MIN-102 levels in plasma samples.

3.4.8. **Packaging, Labeling and Resupply**

Study medication will be provided by Laboratorium Sanitatis SL

MIN-102 will be provided as an oral suspension in 125-mL bottles. Each bottle will contain 100 mL of suspension with a drug concentration of 15 mg/mL.

Each individual bottle will be packaged in an individual box and labelled. Each kit will contain 1 bottle and 1 syringe packed in individual boxes.

The assignment of study medication kits to a subject will be performed at each site by delegated study personnel. The kit numbers dispensed to every subject at every visit will be noted in source documents.

Study drug supplies will be labelled according to applicable regulations.

The Sponsor or the study medication manufacturer will provide study medication to a site on a per subject basis. From there, the medication will be handed to the subject at every visit. Each study medication supply will cover the time until the next study visit, including a reserve amount. Re-supply of study medication may occur due to the long duration of the study. Medication for re-supply will be packaged in an identical manner to the initial supply.

After completion of the study, the study site is required to return all study drug to the sponsor or its representative.

3.4.9. **Storage Conditions**

MIN-102 will be stored at the study site in its original packaging at room temperature (25ºC ± 10 ºC). The investigational product must be stored securely. In the event of temperature deviations outside the range of 15ºC to 35ºC, the sponsor will be notified as soon as possible to determine whether the study drug should be quarantined or can be released.

Subjects and parents/caregivers will be instructed to store drug kits in their original packaging at room temperature and in a cool and dry place (i.e., not in the refrigerator).

3.4.10. **Concomitant Medications**

Any concomitant medications (including over-the-counter medicinal and herbal products) used during the study are to be recorded in the electronic case report form (eCRF), specifying the name of the drug, start and stop dates, daily dose, dose regimen, administration route, and reason for administration.

Subjects and parents/legal guardians must be made aware that they must inform the investigator before taking any new treatment during participation in the study, including over-the-counter medicinal and herbal products.

Table 5 summarizes prohibited and permitted concomitant medications.

Concomitant physiotherapy and/or subject-initiated regular, scheduled physical activities (such as swimming, walking, or exercising in a gym) will be allowed.

Table 5. Permitted and Prohibited Concomitant Medications

**Prohibited and requiring a minimum washout time until screening**	Pioglitazone and other thiazolidinediones (6-month washout)<br>Honokiol (6-month washout)<br>Biotin (MD-1003) at a dose of >50 mg per day (3-month washout)
**Prohibited**	**CYP2C8 inhibitor:** gemfibrozil<br>**Moderate to strong CYP3A inhibitors:**<br>Strong: boceprevir, clarithromycin, conivaptan, grapefruit juice, indinavir, itraconazole, ketoconazole, lopinavir/ritonavir, mibefradil, nefazodone, nelfinavir, posaconazole, ritonavir, saquinavir, telaprevir, telithromycin, voriconazole<br>Moderate: fosamprenavir, imatinib, verapamil, amprenavir, aprepitant, atazanavir, ciprofloxacin, crizotinib, darunavir/ritonavir, diltiazem, erythromycin, fluconazole<br>**CYP3A inducer:** rifampicin
**Allowed, provided dose has been stable for ≥6 months prior to screening and is kept constant during study, or is initiated after decision not to conduct HSCT**	Lorenzo's oil<br>Baclofen<br>Benzodiazepines<br>Opiates and cannabis preparations<br>Fampridrine<br>Antioxidants (e.g. N-acetylcysteine, vitamin E, lipoic acid)
**Allowed**	Benzodiazepines or propofol for sedation during MRI procedures and optional CSF sampling

CYP = cytochrome P450

3.5. Study Assessments and Variables

3.5.1. Efficacy Assessments

3.5.1.1. Clinical Assessments

Clinical function will be assessed by the NFS-MFD scale (Moser et al., 2000). This scale rates 15 domains of neurological status that are most affected in cALD; 6 of these domains rate the most serious symptoms, summarized as "Major Functional Disabilities" (MFD). The scale together with item definitions for standardized rating are attached in Appendix 6.4 and Appendix 6.5 respectively.

3.5.1.2. Cerebral MRI

Magnetic Resonance Imaging assessments will be performed in 3 Tesla MRI machines. All MRI assessments will be performed during the same session in the MRI machine. These MRI sessions will require the study subject to be in the MRI machine for approximately 60 minutes. MRI assessments will be conducted using gadolinium as a contrast medium. Gadolinium will be administered intravenously (IV) and, if unavoidable, the subject may be sedated for the MRI assessments per standard practices at each investigational site. Post contrast sequences will be acquired not earlier than 5 minutes after injection and should be completed 15 minutes after injection at the latest. A separate protocol will be provided to explain how cerebral MRI should be conducted.

MRI scans will be sent for central reading to a specialized vendor (Athinoula A. Martinos Center for Biomedical Imaging, Boston) who is experienced with these imaging techniques and analysis in this patient population. The presence of inflammatory lesions as a qualitative assessment for eligibility and throughout the study will be assessed by local site radiologist or investigator.

The Martinos Center will provide a detailed imaging charter to each participating site prior to start of the study with standardized acquisition parameters.

The MRI parameters obtained at the Screening Visit (V0) and will be considered the baseline values for the efficacy evaluations. If the MRI cannot be scheduled at the Screening Visit (V-1), it will be scheduled at Baseline Visit (V0) prior to first dose of study drug. The first on-treatment MRI may be performed at Visit 2 (Week 4) but is optional. The next on-treatment MRI will be performed at Visit 6 (Week 12) and Visit 8 (Week 24) or the "Visit Prior to HSCT", as applicable. Afterwards, they will be scheduled every 12 weeks and/or at the "Visit Prior to HSCT" for subjects who are eligible for HSCT, and every 24 weeks for subjects who do not undergo HSCT.

In order to minimize subjects' exposure to sedation for the MRI and/or exposure to gadolinium, the Investigator may use a clinical MRI if less than 15 days apart from baseline visit and acquired with same standards as per study protocol. Investigator may also omit post-baseline scheduled MRIs at a given visit, except for V6 and/or the "Visit prior to HSCT", if they are not deemed necessary to monitor progression of cerebral lesions. The investigator may also perform an MRI as an unscheduled examination at any time, if deemed necessary to monitor lesion evolution.

Only macrocyclic gadolinium agents (gadobutrol, gadoteric acid and gadoteridol) are to be used.

3.5.2. Pharmacokinetics and Biomarkers Measurements

3.5.2.1. Blood Sampling

Blood sampling to determine MIN-102 plasma concentrations will be performed at all visits during the treatment period to determine potential dose adjustments to achieve the target plasma exposure range.

At the Baseline Visit, blood samples of 1 mL each will be drawn pre-dose, then at 2, 6, 12, and 24 hours post dose to obtain a full PK profile in children. These post-dose samples will require an overnight stay. Blood samples will be taken via an indwelling IV catheter or direct venipuncture. The exact time of blood sampling will be recorded in the eCRF.

Details on sample collection, handling, storage and shipping will be described in the laboratory manual. At Visits 2 and 6 blood samples will be drawn immediately before MIN-102 administration and at 2 hours post dose. The allowed time window for obtaining post-dose blood samples will be ±15 minutes.

At Visit 8 and all subsequent visits, blood will be drawn pre-dose. Blood will also be drawn at the "Prior to HSCT Visit", i.e. one day after administration of last dose of study drug.

3.5.2.2. **Cerebrospinal Fluid Sampling**

The CSF assessment for biomarkers will be optional for subjects whose parents, legal guardian or authorized legal representative give separate informed consent to lumbar puncture and have no contraindications against lumbar puncture, such as risk of bleeding or significant deformities of the spine.

Qualified medical personnel will perform a lumbar puncture according to best practices at each site to collect a minimum of 3 mL, but not more than 5 mL, of CSF samples for analysis of biomarkers. Samples will be collected before the first intake of study medication at V0 and before study medication intake at V6. The time of CSF sampling and the time of the last intake of study medication before sampling will be recorded.

The investigator will aim at performing the lumbar puncture, if possible, while the patient is sedated for the MRI investigations.

3.5.2.3. **Drug Concentration and Biomarker Measurements**

The analysis of MIN-102 and M3 in plasma will be performed at bioanalytical laboratory using validated liquid chromatography-mass spectrometry/mass spectrometry methods. The analysis will be conducted in a Good Laboratory Practice (GLP)-compliant facility and laboratory procedures will be in accordance with the current GLP guidelines for the Organization for Economic Cooperation and Development.

Biomarkers analyzed in plasma and CSF will include: neurofilament light chain (NfL), adiponectin, matrix metalloproteinase (MMP)-9, interleukin (IL)-18, interleukin (IL)-1β, interleukin-1 receptor antagonist (IL-1ra), monocyte chemoattractant protein-1 (MCP-1) and chitotriosidase.

Further parameters may be added with emerging science. Blood (and CSF if applicable) sample remnants will be stored for this purpose for a maximum of 25 years to support future research. Consent to long-term storage is optional and requires additional informed consent.

3.5.3. **Assessment of Safety and Tolerability Measurements**

Safety and tolerability assessments will comprise recording of AEs, clinical laboratory parameter assessment, vital signs, 12-lead ECG, echocardiogram and physical examination. Assessments will be performed in accordance with Table 1.

3.5.3.1. **Adverse Events**

An AE is any untoward medical occurrence associated with the use of a drug in humans, whether or not considered drug related. An adverse event (also referred to as an adverse experience) can be any unfavorable and unintended sign (e.g. an abnormal laboratory finding), symptom, or disease temporally associated with the use of a drug, without any judgment about causality.

Pre-existing conditions (present before intake of study drug) or pretreatment AEs (onset before intake of study drug) are considered concomitant diseases and should not be recorded as AEs but should be recorded on the concomitant diseases eCRF page. However, if the subject experiences a worsening, increased frequency, or complication of such a concomitant disease, the worsening, increased frequency,

or complication should be recorded as an AE. Investigators should ensure that the AE term recorded captures the change in the condition (e.g. "worsening of…").

Test findings (not including PK results) and physical examination findings are considered AEs if they:

- Are associated with accompanying symptoms, and/or
- Require additional diagnostic testing or medical/surgical intervention, and/or
- Lead to a change in study drug dosing (excluding PK results) or discontinuation from the study; result in the addition of significant additional concomitant drug treatment or other therapy, and/or
- Lead to any of the outcomes included in the definition of an SAE, and/or
- Are considered to be an AE by the investigator or sponsor

Pre-planned procedures (surgeries or therapies) that were scheduled prior to the start of data collection are not considered AEs. However, if a pre-planned procedure is performed early (e.g. as an emergency) due to a worsening of the pre-existing condition, the worsening of the condition should be reported as an AE.

Cases of drug overdose without manifested side effects are not considered AEs.

For more detailed information and for Serious Adverse Events see Section 6.1

3.5.3.2. **Laboratory Safety Tests.**

Blood and urine samples for clinical laboratory assessments will be collected at study visits as indicated in Tables 1 and 8. Blood samples will be taken in fasting state (if possible). The following parameters will be analyzed:

- ***Testing for hepatitis B and C, and HIV at visit V-1***[1].
- Blood chemistry: cortisol, adrenocorticotropic hormone (ACTH), aldosterone (only at V-1), and HbA1c (only at V-1, Visit Prior to HSCT or FUV); total bilirubin, alkaline phosphatase, AST, ALT (at all visits except Visit 0); gamma glutamyl transferase, lactate dehydrogenase, creatinine, urea, uric acid, cholesterol, triglycerides, total protein, albumin, glucose, inorganic phosphate, sodium, potassium, calcium, and chloride (at all visits except Visit 0, 1, 3, 4, 5 and 7); NT-proB-type Natriuretic Peptide (NT-proBNP) (at all visits except Visit -1, 1, 3, 4, 5 and 7).
- Prothrombin time (at all visits except Visit -1); for patients of less than 13.75 kg (30.31 lbs) of body weight, prothrombin time will only be analyzed if liver parameters show alterations suspicious of DILI (see 6.2).
- Hematology: leukocytes, erythrocytes, hemoglobin, hematocrit, thrombocytes, partial automated differentiation: lymphocytes, monocytes, eosinophils, basophils, neutrophils, mean corpuscular volume, mean corpuscular hemoglobin (MCH), and mean corpuscular hemoglobin concentration (at all visits except Visit 0, 1, 3, 4, 5 and 7).

---

[1] ***Only applicable in France according to the current approved protocol version 6.2***

- Blood spot glucose check (at all visits starting at V0)
- Urinalysis (qualitative): hemoglobin, urobilinogen, ketones, glucose, protein, bilirubin, leukocytes, pH, and nitrite (at all visits except Visit 0, 1, 3, 4, 5 and 7).
- Cytological examination will assess the presence of abnormalities in bladder epithelial cells (at all visits except Visit 0, 1, 3, 4, 5 and 7).

In the event the investigator considers a laboratory abnormality in the biochemistry or hematology analysis as "clinically relevant", additional samples for repeated analysis should be collected within a reasonable time, as deemed appropriate and sent to the central laboratory for analysis. A comment should be provided for these abnormalities on the laboratory report. These laboratory samples should be identified as "unscheduled" samples.

The clinical laboratory will flag laboratory test values that are outside of the normal range and the investigator will give his/her overall interpretation of the results. Clinically significant findings will be recorded as AEs and a relationship to study medication will be indicated and recorded in the CRF.

3.5.3.3. **Vital Signs**

Systolic and diastolic blood pressure and pulse rate will be recorded after the subject has been resting in the supine position for at least 5 minutes. Assessments will be made using an automated device using the same arm throughout the study, if possible. Body temperature and weight will be measured subsequently. Height will be measured at screening only.

Subjects can be contacted by phone between scheduled visits and be advised to monitor their weight frequently and report back to the investigator in case of weight gain. If required, an unscheduled visit may be scheduled at any time.

3.5.3.4. **Electrocardiogram**

A standard 12-lead ECG will be recorded after the subject has been resting in the supine position for at least 5 minutes. The following ECG parameters will be recorded using an ECG machine equipped with computer-based interval measurement heart rate, PR-interval, RR-interval, QRS-duration, QT-interval, and QTcF-interval (Fridericia's).

The investigator will provide interpretation of the ECG profile. The ECG must be recorded in triplicate with 3 serial readings performed 5 minutes apart approximately 2 hours (±0.5 hours) after the daily dose has been administered.

3.5.3.5. **Echocardiogram**

An echocardiogram will be performed only at Screening. It will be recorded locally and assessed by the local cardiologist or qualified delegate.

Subjects developing peripheral edema or NT-proBNP elevations >1.5 times the baseline value and out of normal range will require the echocardiogram repeated at each regular on-site visit (V1 – Vx), until resolution of the peripheral edema or elevated NT-proBNP. Changes in echocardiogram from baseline value for all subjects developing peripheral edema will be evaluated.

3.5.3.6. **Physical examination**

Physical examination will be performed at the designated on-site visits. Physical examination includes evaluation of the heart, lungs, abdomen, extremities, and skin, with specific attention to the presence of peripheral edema and signs or symptoms of heart failure. The physical examination will be conducted by the investigator or a medically qualified delegate with MD degree. At V1, V3, V4, V5, and V7 the patient will be only examined for the presence of peripheral edema. If this is a home visit, the examination may be performed by the home nurse.

3.5.3.7. **Palatability assessment**

The palatability of study drug will be evaluated by the subject himself (depending on the age and cognitive ability), or by the parents' rating of ease of administration and subject's facial expression and/or verbal expression on a five-point hedonic from "super-good" to "super-bad" (Guinard, 2000).

3.5.4. **Total Blood sampling volumes**

Total volume of blood drawn during the study depends on each patient's duration in the trial. Table 6 outlines the number and volume of blood samples that would be collected per patient during the first 8 weeks (until V3) and thereafter. Table 8 in Section 6.3 details the exact number of samples and volume collected. If deemed necessary by the investigator or Sponsor, an extra blood sample may be taken, for example for safety reasons.

The planned blood sampling volumes do not exceed any of the following:

- 5% of the total blood volume over a period of 8 weeks.
- 3% of the total blood volume over a period of 4 weeks.
- 1% of the total blood volume at any single time.

Table 6. Total Blood sampling volumes per patient.

Assessment			Volume per sample	Number of samples			Total volume (mL) during the study		
				V-1 to V3 (first 8 weeks)	V-1 to Visit prior to HSCT[1]	V8, V9, V10…[2] + FUV	V-1 to V3 (first 8 weeks)	V-1 to Visit prior to HSCT[1]	V8, V9, V10…[2] + FUV
**MIN-102 in plasma**			1 mL	7	10	1	7	10	1
**Biomarkers**			2 mL	2	4	1	4	8	2
**Clinical laboratory tests**	Blood chemistry	Cortisol, ACTH, aldosterone	6 mL	1	1	-	6	6	-
		Hemoglobin A1c	1 mL	1	2	1	1	2	1
		Total bilirubin, AP, AST, ALT, gamma glutamyl transferase, lactate dehydrogenase, creatinine, urea, uric acid, total protein, albumin, glucose, inorganic phosphate, sodium, potassium, calcium, and chloride	2 mL	4	9	2	8	18	4
		NT-proB-type Natriuretic Peptide (NT-proBNP)	2 mL	2	4	2	4	8	4
	Hematology		1 mL	2	4	2	2	4	2
	Prothrombin time[3]		2 mL	4	9	2	8	18	4
**Total volume of blood sampled**							40	74	18

[1] Assumes HSCT takes place after V7
[2] Each individual visit including the Follow-up visit
[3] Patients whose body weight is lower than 13.75Kg (30.31 Lbs.) will only be drawn blood samples for prothrombin time analysis if liver parameters show alterations suspicious of DILI.

3.6. Statistics

3.6.1. Analysis Sets

The analysis sets are defined in Table 7. The primary analyses will be conducted using the mITT analysis set. In addition, a per-protocol (PP) analysis will be conducted.

Table 7. Analysis Sets

Analysis Set	Subjects Included
Safety analysis set	All subjects who took at least 1 dose (partial or complete) of MIN-102
mITT analysis set	All subjects who took at least 1 dose (partial or complete) of MIN-102 and had at least 1 post-baseline MRI assessment with gadolinium
PP analysis set	All subjects in the mITT analysis set without a major protocol violation
PK analysis set	All subjects who took at least 1 dose (partial or complete) of MIN-102 and had at least 1 plasma sample with MIN-102 concentration

MRI = magnetic resonance imaging; mITT = modified intent-to-treat; PP = per-protocol

3.6.1.1. Protocol Deviations

All protocol deviations will be summarized in the clinical study report. After database closure, the study team will assess all protocol deviations on a subject-by-subject basis to ascertain how the deviation affects subject inclusion into the various analysis sets. Any deviation may affect the subject's eligibility for 1 or more of the analysis sets. The impact of protocol deviations on assessment of the primary endpoint and the handling of missing/invalid data will be carefully investigated.

3.6.1.2. Statistical and Analytical Plan

Full details of the statistical methods to be used for analysis of study data will be provided in the Statistical Analysis Plan.

3.6.1.3. Efficacy Evaluation

Comparisons will be made for all efficacy and safety parameters, as available at the given time points:

- Visit 8 (24 weeks after start of treatment) or Visit Prior to HSCT if earlier, and
- Visit 12 (96 weeks after start of treatment) or Visit Prior to HSCT if earlier

Primary efficacy endpoint.
The primary efficacy endpoint will be the number of patients meeting "arrested disease" criteria at Visit 12.

A first assessment will be conducted when up to 13 evaluable patients have reached 24 weeks of treatment. The second timepoint represents the confirmatory timepoint.

"Arrested disease" is defined using the following parameters:

- Change in Neurological Function Score (NFS) from Baseline ≤1 point (Week 24) or ≤5 points (Week 96)
- Free of MFD
- Lack of lesion progression on MRI, fulfilling any of the following:
  - No conversion to Gd+ lesions (Population 1) defined as GIS score of 0.
  - Disappearance of Gd+ lesions (Population 2) defined as change in GIS score from 1, 2 or 3 to 0
  - No significant growth of T2/FLAIR lesions as assessed by central reading
  - No significant growth of cerebral lesions in the overall assessment of the investigator

The study will be continued when a minimum of 4 of 13 evaluable patients have met the endpoint of "arrested disease" at Week 24 or if the overall assessment from central reading suggests lesion growth deceleration ("continuation criteria"). Lesion deceleration shall be considered when lesion growth determined by central readers assessment is below what would be expected based on the literature (Liberato et al., 2019, Mallack et al., 2019, 2020 and 2021) and/or when lesion growth rate of the latest measure is lower than previous ones. Growth rate is defined as the increase in T2/FLAIR lesion volume since previous MRI divided by the number of months elapsed since that previous MRI.

The study will be considered successful if a minimum of 4 patients meet all "arrested disease" criteria at Week 96, except for MFD where >70% of all evaluable patients at Week 96 have to be free of MFD.

Secondary efficacy endpoints

The secondary efficacy endpoints will be:

- Sustained change from Baseline in the score composed of NFS items 1 (hearing/auditory processing), 2 (aphasia/apraxia), 4 (vision impairment), 10 (spastic gait) and 13 (incontinence)

- Sustained change from Baseline in total NFS score
  "Sustained change" is defined as the same total score for NFS items 1, 2, 4, 10, and 13 and no change >1 in NFS total score observed in two the consecutive Visits 6–8, and Visits 11-12, respectively. If "sustained change" definition is not met, the average of the two scores of Visit 6 and 8, and Visit 11 and 12, respectively, will be used. If NFS total score differs by 1 point between V6-8, or V11-12, the higher of the two scores is used.
- Change from baseline in Loes MRI severity score
- Change from baseline in Gadolinium Intensity Score (GIS)
- Overall survival of patients who have not undergone HSCT
- Number of patients meeting HSCT criteria

Exploratory efficacy endpoints

The exploratory endpoints will be:

- Change from Baseline in T2/FLAIR lesion volume
- Change from Baseline in white matter fiber structure as determined by fractional anisotropy (FA) and/or apparent diffusion coefficient (ADC) on DTI
- Change from Baseline in cerebral blood flow and blood volume, capillary mean transient time heterogeneity (CHT) and constant of permeability (K2 and Kapp) as determined by DSC MRI
- Changes from Baseline in the levels of each biomarker in plasma and CSF (optional)

Further details on the statistical procedures are provided in a separate Statistical Analysis Plan

3.6.1.4. **Pharmacokinetic Evaluation**

Pharmacokinetic data will be summarized using appropriate descriptive statistics and listed and summarized in tabular and/or graphical form.

The PK endpoints include the following parameters: AUC, maximum plasma concentration (C_{max}), C_{min}. Additional PK analyses may be performed if deemed appropriate.

3.6.1.5. **Biomarker Evaluation**

Biomarker data will be summarized using descriptive statistics and listed and summarized in tabular and/or graphical form. Correlation methods will be used to investigate the relationship between biomarkers and disease progression.

Biomarkers analyzed in plasma and CSF will include: neurofilament light chain (NfL), adiponectin, matrix metalloproteinase (MMP)-9, interleukin (IL)-18, interleukin (IL)-1β, interleukin-1 receptor antagonist (IL-1ra), monocyte chemoattractant protein-1 (MCP-1) and chitotriosidase. Further parameters may be added with emerging science.

3.6.1.6. **Evaluation of Safety and Tolerability**

Safety assessments will focus on the type, severity, and frequency of individual AEs and laboratory tests, vital signs, and ECG abnormalities. The analyses will generally be descriptive in nature and will be based on the safety population.

Adverse event data will be displayed in listings by subject. The number and percentage of subjects with AEs will be tabulated by system organ class (SOC) and preferred term (PT). A subject with multiple AEs within a SOC or PT will be counted once toward the total for the total for the SOC or PT. Further AE tables, for example, by severity, relationship, or outcome will be presented if the number of events makes such information useful.

Other safety parameters will be displayed by visit and other key variables as applicable. Summary statistics for continuous variables will include: n (number of subjects with non-missing values), mean, SD, median, minimum, and maximum. Statistics for categorical variables will consist of counts and percentages of subjects falling into each category (frequency tables). If appropriate, change from baseline will be described using summary statistics for continuous variables and by shift tables (including absolute and relative frequencies) for categorical variables.

The Medical Dictionary for Regulatory Activities (MedDRA [latest version]) will be used for coding AEs and medical history (past and/or concomitant disease). The World Health Organization Drug Dictionary (latest version) will be used to code concomitant and previous medications.

The safety endpoints include the following:

- Adverse events (AEs)
- Serious adverse events (SAEs) and suspected unexpected serious adverse reactions (SUSARs)
- Vital signs (body weight, height, blood pressure, pulse rate, and body temperature)
- Physical examination
- 12-lead ECG (heart rate and the following intervals: PR, RR, QRS, QT, QT corrected for heart rate using Fridericia's formula [QTcF]).
- Echocardiogram
- Clinical laboratory tests

Tolerability endpoints apply to the period of MIN-102 treatment. They include the safety endpoints listed above and the palatability of study drug (parent rating of ease of administration and subject's facial expression).

3.6.2. **Interim Analysis**

No formal interim analysis for efficacy is planned for this study. However, the first assessment of the number of patients fulfilling "arrested disease" criteria at Week 24 (Visit 8) will inform the decision on study continuation.

3.6.3. Determination of Sample Size

Sample size has been calculated to estimate the proportion of patients meeting "arrested disease" criteria to distinguish a rate of 40% with treatment vs. a rate of spontaneous 10% "arrested disease" in untreated patients. With 80% power, the study would enroll 13 participants and use a criterion of at least 4 participants achieving "arrested disease" for meeting the primary study objective with a one-sided significance level of 0.05.

The null hypothesis is that the proportion of patients meeting "arrested disease" criteria will be less than or equal to 10% (threshold comes from well-established natural history), and the alternative hypothesis is that the proportion will be greater than 10%.

The primary efficacy endpoint analysis will be based on a one-sided 95% confidence interval for the proportion of evaluable subjects meeting "arrested disease." If the lower bound of the one-sided 95% confidence interval is greater than 10%, then the null hypothesis will be rejected in favor of the alternative hypothesis.

Additional subjects may be enrolled after agreement between the investigator and the Sponsor to ensure that the study recruits 13 evaluable subjects who receive MIN-102 treatment. Decisions regarding subject replacement will be documented. Patients who meet "HSCT criteria" and patients who at week 96 do not meet "arrested disease" criteria will not be replaced. However, patients who drop out by withdrawal of consent by parents or termination of treatment by the investigator for non-treatment related causes may be replaced. Patients stopping treatment will be encouraged to remain in the study and attend all further scheduled efficacy and safety assessments.

4. STUDY MANAGEMENT

4.1. Approval and Consent

4.1.1. Regulatory Guidelines

The study will be performed under GCP in accordance with the guidelines of the ICH, Declaration of Helsinki, and in accordance with U.S. Investigational New Drug regulations (21 Code of Federal Regulations [CFR] 312) and local laws (as applicable).

4.1.2. Institutional Review Board (IRB)/Independent Ethic Committee (IEC)

Conduct of the study must be approved by an appropriately constituted IRB or IEC. Approval is required for the study protocol, Investigator's Brochure, protocol amendments, consent forms, subject information sheets, and advertising materials for recruitment of subjects. No drug will be shipped to a site until written IRB/IEC authorization has been received by the sponsor or its representative.

In the U.S., the investigator is also responsible for notifying the IRB of any reportable serious adverse drug reactions from any other study conducted with the investigational product. Minoryx Therapeutics S.A. will provide this information to the investigator. Outside the US, Sponsor or CRO will perform notification or submissions to IRBs.

Progress reports, notifications of serious adverse drug reactions, and predefined protocol deviations will be provided to the IRB/IEC according to local regulations and guidelines.

4.1.3. Written Informed Consent

For each study subject, written informed consent must be obtained from the parent/legal guardian prior to initiating any protocol-related procedures. As applicable, assent of the subject will be obtained. As part of this procedure, the investigator or an associate must explain orally and in writing the nature, duration, and purpose of the study and the action of the drug in such a manner that the subject/parent is aware of the potential risks, inconveniences, or adverse effects. The parent/legal guardian should be informed that the subject may withdraw from the study at any time. The consent process should provide the parent/legal guardian with all information required by local regulations and ICH guidelines. The investigator will provide the sponsor or its representative with a copy of the IRB-approved consent form prior to the start of the study.

During the consent process, the parent/legal guardian should be informed that the results of the screening evaluations may indicate that the subject is eligible for HSCT. The parent/legal guardian should be asked whether he/she elects for HSCT to be undertaken if his/her child is eligible. The parent/legal guardian will be asked to consent for the subject, if HSCT eligible, to receive treatment with MIN-102 and attend the scheduled visits until procedures for HSCT are initiated, whenever this occurs.

The informed consent discussion will also include the possible scenario of continued treatment with MIN-102 if the subject is found to be ineligible for HSCT after the Baseline Visit, for example, because of stabilization or worsening of disease or inability to find a suitable donor. After the decision is made not to conduct HSCT, continued treatment with MIN-102 will be offered to the subject as part of the study until the first end-of-treatment criteria occurs.

4.2. Financing and Insurance

Prior to study commencement, the sponsor (or its designee) and the investigator (or the institution, as applicable) will agree on costs necessary to perform the study. This agreement will be documented in a financial agreement that will be signed by the investigator (or the institution signatory) and the sponsor (or its designee).

The Sponsor has insurance coverage for trial-related, medicine-induced injury, and other liabilities incurred during clinical trials which will provide compensation for any study-related injury. The Sponsor will provide local country-specific insurance, as required.

4.3. Discontinuation of the Study by the Sponsor

The sponsor reserves the right to discontinue the study at a site or multiple sites for safety or administrative reasons at any time. In particular, a site that does not recruit at a reasonable rate may be discontinued. Should the study be terminated, and/or the site closed for whatever reason, all documentation, clinical supplies, and study drug pertaining to the study must be returned to the sponsor or its representative.

4.4. Changes to Final Study Protocol

All protocol amendments must be submitted to the IRB. Protocol modifications that affect subject safety, the scope of the investigation, or the scientific quality of the study must be approved by the IRB before implementation of such modifications to the conduct of the study. If required by local law, such modifications must also be approved by the appropriate regulatory agency prior to implementation. However, Minoryx Therapeutics S.L., at any time, can amend this protocol to eliminate an apparent

immediate hazard to a subject. In this case, the appropriate regulatory authorities will be notified subsequent to the modification. In the event of a protocol modification, the subject consent form may require similar modifications with subsequent IRB approval and re-consenting of subjects.

4.5. Notification of Study Completion or Discontinuation

The Health Authority and the IRB/IEC in each country will be notified per applicable regulations.

4.6. Quality Assurance and Quality Control

4.6.1. Study Monitoring

This study will be regularly monitored at all stages of development by the clinical research personnel employed by the sponsor or its representative contract research organization to ensure adherence to the study protocol, to the International Conference on Harmonization (ICH) GCP and any applicable regulatory requirements.

The progress of the study will be monitored by on-site visits and written, e-mail, and telephone communications between personnel at the study site and the sponsor. The investigator will allow sponsor monitors or designee(s) to inspect all eCRFs; subject records (source documents); signed consent forms; records of study drug receipt, storage, and disposition; and regulatory files related to the study.

The study monitor is responsible for visiting sites at regular intervals throughout the study according to the clinical monitoring plan to verify adherence to the protocol; completeness, accuracy, and consistency of the data; and adherence to ICH GCP and local regulations on the conduct of clinical research. The monitor is responsible for inspecting the eCRFs and ensuring completeness of the study essential documents. The monitor should have access to subject medical records and other study-related records needed to verify the entries on the eCRFs.

The monitor will communicate any deviations from the protocol, standard operating procedures, GCP, and applicable regulations to the investigator and will ensure that appropriate action designed to prevent recurrence of the detected deviations is taken and documented.

By signing this protocol, the investigator agrees to cooperate with the monitor to ensure that any problems detected during these monitoring visits are addressed and documented.

Accurate and reliable data collection will be assured by the study monitor's 100% verification and cross-check of the eCRFs against source documents and the investigator's records. Note that a variety of original documents, data, and records may be considered as source documents in this study. Any data to be recorded directly in the eCRF (to be considered as source data) will be identified at the start of the study.

The investigator and appropriate personnel will be requested to attend meetings and/or trainings required by the sponsor or its representative contract research organization to assure acceptable protocol execution.

Medical advisors and clinical research associates or assistants may request to witness subject evaluations occurring as part of this protocol.

4.6.2. **Audits and Inspections**

In accordance with ICH GCP and the sponsor's audit plans, this study may be selected for audit by representatives from the sponsor. Inspection of site facilities (e.g. pharmacy, drug storage areas) and review of study-related records will occur to evaluate the study conduct and compliance with the protocol, ICH GCP, and applicable regulatory requirements. The study may also be subject to audit or inspection by regulatory authorities or the IRB/IEC.

If such an audit occurs, the investigator must allow access to required subject records. By signing this protocol, the investigator grants permission to personnel from the sponsor, its representatives, appropriate regulatory authorities, and the IRB/IEC for on-site monitoring of all appropriate study documentation, as well as on-site review of the procedures employed in eCRF generation, as clinically appropriate. Direct access includes permission to examine, analyze, verify, and reproduce any records and reports that are important to the evaluation of a clinical study.

4.6.3. **Data Collection**

All relevant observations and data related to the study, as per the study protocol, will be recorded on eCRF pages. A representative of sponsor or its designee will provide instructions for completing the eCRF. Adequate and accurate case records should be maintained, including the evaluation of inclusion and exclusion criteria, medical history, physical examinations, imaging and clinical assessments, a record of clinical safety laboratory sample collection, drug administration, AEs, and final evaluation.

The eCRFs must be completed for each subject who signs a consent form and undergoes any screening procedure. For subjects who are screened but not randomized, minimal data will be recorded on the eCRF, including demography, subject status, and AEs. All study-related data for these subjects will be maintained in the medical records at the site. All study-related data for subjects will be maintained in the medical records at the site.

The eCRF data entry will be completed on the day of the visit or as soon as possible thereafter. The investigator must electronically sign and date the eCRF. The signature will indicate that the investigator has reviewed the data and data queries recorded on eCRFs and the site notifications and that he/she agrees with the content. After the completion of the study, eCRFs, including the audit trail, will be returned to the sponsor and stored in the archives.

4.6.4. **Data Management**

Each subject will be identified in the database by a unique subject identification number.

To ensure the quality of clinical data across all subjects and sites, a clinical data management review on subject data will be performed according to specifications given to the sponsor or designee. Data will be vetted electronically and/or manually as appropriate. During this review, subject data will be checked for consistency, omissions, and any apparent discrepancies. In addition, the data will be reviewed for adherence to the protocol and GCP.

For eCRFs, the data will be electronically vetted by programmed data rules within the application. Queries generated by rules and/or manually raised by reviewers will be generated within the electronic data capture application and also resolved within the eCRF application by the investigator.

Data received from external sources such as central laboratories will be reconciled with the clinical database.

Martinos Center will be the central storage site for all MRI data, and MRI data obtained at all sites will be centrally evaluated there.

Serious AEs in the clinical database will be reconciled with the safety database.

All medical history (except terms pre-specified on the eCRF) and AEs will be coded using the Medical Dictionary for Regulatory Activities. All prior and concomitant medications will be coded using the World Health Organization Drug Dictionary.

At the time of database lock, the clinical database will undergo a quality control audit check to ensure accuracy of the data. The audit will involve a comparison of eCRF values with values from data listings generated from the clinical database. Values identified as critical safety and efficacy variables will be confirmed for 100% of the subjects. In addition, a random sample of subjects will be selected to have all data values checked. The number of subjects whose data will be randomly reviewed will be determined to provide sufficient checks of accuracy of the clinical database.

4.6.5. **Storage and Retention of Study Records**

The investigator and study staff are responsible for maintaining a comprehensive and centralized filing system (Investigator Site File) of all study-related (essential) documentation, suitable for inspection at any time by representatives from the sponsor and/or applicable regulatory authorities.

Essential documents include the following:

- Subject files containing completed consent forms and supporting copies of source documentation (if kept)
- Study files containing the protocol with all amendments, Investigator's Brochure, copies of relevant essential documents required prior to starting a clinical study, and all correspondence to and from the IRB/IEC and the sponsor.
- Records related to the investigational product, including acknowledgment of receipt at site, accountability records, final reconciliation, and applicable correspondence

In addition, all original source documents must be maintained by sites and be readily available. Source documents include all recordings and observations or notations of clinical activities and all reports and records supporting entries in the eCRFs and necessary for the evaluation and reconstruction of the clinical study.

Essential documents (e.g. protocol and amendments, IRB/IEC correspondence and approvals, approved and signed consent forms, Investigator's Agreement, clinical supplies receipts, distribution and return records, inventory of study product), records of subjects, source documents, monitoring visit logs, data correction forms, and other sponsor correspondence pertaining to the study must be kept in appropriate study files in a secure location at the site.

The investigator must arrange for retention of study records at the site. The duration of the retention period must meet the requirements of the relevant regulatory authority. The investigator should take measures to prevent accidental or premature destruction of these documents.

No study document should be destroyed without prior written agreement between the sponsor and the investigator. Should the investigator wish to assign the study records to another party or move them to

another location, he/she must notify the sponsor in writing of the new responsible person and/or the new location.

Prior to transfer or destruction of study records, the sponsor must be notified in writing and be given the opportunity to further store such records.

The investigator agrees to comply with all applicable laws and regulations related to the privacy and protection of patient health information.

4.6.6. **Subject Confidentiality**

All laboratory specimens, evaluation forms, reports, and other records will be identified in a manner designed to maintain patient confidentiality.

Records will be kept in a secure storage area with limited access.

Clinical information will not be released without the written permission of the patient (or the patient's guardian), except as necessary for monitoring and auditing by the Sponsor, its designee, the authorities, or the EC.

Other patient confidentiality will be addressed in the clinical trial agreement with the sites and in the informed consent form signed by each study participant.

The investigator must ensure that the study is performed in accordance with the applicable data protection laws, including Regulation (EU) 2016/679 of the European Parliament and of the Council of 27 April 2016 on the protection of natural persons with regard to the processing of personal data and on the free movement of such data, and repealing Directive 95/46/EC (General Data Protection Regulation or GDPR). The investigator must ensure that the subject's anonymity is maintained and keep in strict confidence documents not de-identified (e.g. signed informed consent form). The investigator must also provide all reasonable assistance to the sponsor in the exercise of their duties as Data Controller (as defined by the GDPR) for the study.

4.7. **Use of Study Findings**

By signing the study protocol, the investigator agrees to the use of study results for the purposes of national and international registration. If necessary, the authorities will be notified of the investigator's name, address, qualifications, and extent of involvement. Reports covering clinical and biometric aspects of the study will be prepared by the sponsor or its representative.

4.8. **Publication Policy**

The intention of this study will be to publish the results at study conclusion. All information obtained during the conduct of the study will be regarded as confidential, and written permission from the sponsor is required prior to disclosing any information related to the study. A formal publication of data collected as a result of the study will be considered a joint publication by all investigators and appropriate sponsor personnel. Authorship will be determined by mutual agreement. Submission to the sponsor for review and comment is required prior to submission to the publisher. This requirement should not be construed as a means of restricting publication, but is intended solely to ensure concurrence regarding data, evaluations, and conclusions, and to provide an opportunity to share with the investigator any new or unpublished information of which he or she may be unaware.

5. LIST OF REFERENCES

Actos® SmPc (09 June 2016).

Cartier N, Aubourg P. Hematopoietic stem cell transplantation and hematopoietic stem cell gene therapy in X-linked adrenoleukodystrophy. Brain Pathol. 2010 Jul;20(4):857-62.

Eichler, F. (2016) Research update on ALD. ALD Life meeting 26th May, 2016. London

Eichler F, Duncan C, Musolino PL, Orchard PJ, De Oliveira S, Thrasher AJ, Armant M, Dansereau C, Lund TC, Miller WP, Raymond GV, Sankar R, Shah AJ, Sevin C, Gaspar HB, Gissen P, Amartino H, Bratkovic D, Smith NJC, Paker AM, Shamir E, O'Meara T, Davidson D, Aubourg P, Williams DA. Hematopoietic Stem-Cell Gene Therapy for Cerebral Adrenoleukodystrophy. N Engl J Med. 2017 Oct 26;377(17):1630-1638.

Engelen, M., S. Kemp, M. de Visser, B. M. van Geel, R. J. Wanders, P. Aubourg and B. T. Poll-The (2012). "X-linked adrenoleukodystrophy (X-ALD): clinical presentation and guidelines for diagnosis, follow-up and management." Orphanet J Rare Dis **7**: 51.

Feinstein, D. L., E. Galea, V. Gavrilyuk, C. F. Brosnan, C. C. Whitacre, L. Dumitrescu-Ozimek, G. E. Landreth, H. A. Pershadsingh, G. Weinberg and M. T. Heneka (2002). "Peroxisome proliferator-activated receptor-gamma agonists prevent experimental autoimmune encephalomyelitis." Ann Neurol **51**(6): 694-702.

Guinard, J.-X. (2000). "Sensory and consumer testing with children." Trends in Food Science & Technology 11(8): 273-283.

Hunter, R. L., N. Dragicevic, K. Seifert, D. Y. Choi, M. Liu, H. C. Kim, W. A. Cass, P. G. Sullivan and G. Bing (2007). "Inflammation induces mitochondrial dysfunction and dopaminergic neurodegeneration in the nigrostriatal system." J Neurochem **100**(5): 1375-1386.

Liberato, A.P., Mallack, E.J., Aziz-Bose, R., Hayden, D., Lauer, A., Caruso, P.A., Musolino, P.L., and Eichler, F.S. (2019). MRI brain lesions in asymptomatic boys with X-linked adrenoleukodystrophy. Neurology.

Mallack, E.J., Turk, B., Yan, H., and Eichler, F.S. (2019). The Landscape of Hematopoietic Stem Cell Transplant and Gene Therapy for X-Linked Adrenoleukodystrophy. Curr Treat Options Neurol *21*, 61.

Mallack, E.J., van de Stadt, S., Caruso, P.A., Musolino, P.L., Sadjadi, R., Engelen, M., and Eichler, F.S. (2020). Clinical and radiographic course of arrested cerebral adrenoleukodystrophy. Neurology *94*, e2499–e2507.

Mallack, E.J., Askin, G., van de Stadt, S., Caruso, P.A., Musolino, P.L., Engelen, M., Niogi, S.N., and Eichler, F.S. (2021). A Longitudinal Analysis of Early Lesion Growth in Presymptomatic Patients with Cerebral Adrenoleukodystrophy. AJNR Am J Neuroradiol ajnr;ajnr.A7250v1.

Miller, W. P., S. M. Rothman, D. Nascene, T. Kivisto, T. E. DeFor, R. S. Ziegler, J. Eisengart, K. Leiser, G. Raymond, T. C. Lund, J. Tolar and P. J. Orchard (2011). "Outcomes after allogeneic hematopoietic cell transplantation for childhood cerebral adrenoleukodystrophy: the largest single-institution cohort report." Blood **118**(7): 1971-1978.

Miller, W. P., L. F. Mantovani, J. Muzic, J. B. Rykken, R. S. Gawande, T. C. Lund, R. M. Shanley, G. V. Raymond, P. J. Orchard and D. R. Nascene (2016). "Intensity of MRI Gadolinium Enhancement in Cerebral Adrenoleukodystrophy: A Biomarker for Inflammation and Predictor of Outcome following Transplantation in Higher Risk Patients." AJNR Am J Neuroradiol **37**(2): 367-372.

Leriglitazone (MIN-102) Investigator's Brochure.

Moser, H. W., D. J. Loes, E. R. Melhem, G. V. Raymond, L. Bezman, C. S. Cox and S. E. Lu (2000). "X-Linked adrenoleukodystrophy: overview and prognosis as a function of age and brain magnetic resonance imaging abnormality. A study involving 372 patients." Neuropediatrics **31**(5): 227-239.

Pierpont, E. I., J. B. Eisengart, R. Shanley, D. Nascene, G. V. Raymond, E. G. Shapiro, R. S. Ziegler, P. J. Orchard and W. P. Miller (2017). "Neurocognitive Trajectory of Boys Who Received a Hematopoietic Stem Cell Transplant at an Early Stage of Childhood Cerebral Adrenoleukodystrophy." JAMA Neurol **74**(6): 710-717.

Ramkalawan, H., Y. Z. Wang, A. Hurbungs, Y. F. Yang, F. F. Tian, W. B. Zhou, J. Li, H. Yang, B. Xiao and W. Zhang (2012). "Pioglitazone, PPARgamma agonist, attenuates experimental autoimmune neuritis." Inflammation 35(4): 1338-1347.

Raymond, G.V., Aubourg, P., Paker, A., Escolar, M., Fischer, A., Blanche, S., Baruchel, A., Dalle, J.-H., Michel, G., Prasad, V., et al. (2019). Survival and Functional Outcomes in Boys with Cerebral Adrenoleukodystrophy with and without Hematopoietic Stem Cell Transplantation. Biol. Blood Marrow Transplant. *25*, 538–548.

Simcyp MIN-102 Final Report 19-Oct (2018). PBPK Model Development For MIN-102 And Estimation of Starting Doses For Pediatric Evaluation.

Upreti, V. V. and J. L. Wahlstrom (2016). "Meta-analysis of hepatic cytochrome P450 ontogeny to underwrite the prediction of pediatric pharmacokinetics using physiologically based pharmacokinetic modeling." J Clin Pharmacol **56**(3): 266-283.

6. APPENDICES

6.1. Adverse Events

6.1.1. Definitions

6.1.1.1. Serious Adverse Event

An adverse event is considered "serious" if, in the view of either the investigator or sponsor, it results in any of the following outcomes:

- Death
- A life-threatening adverse event. This refers to an event that, in the view of either the investigator or sponsor, places the subject at immediate risk of death. It does not include an AE that, if it had occurred in a more severe form, might have caused death.
- Inpatient hospitalization or prolongation of existing hospitalization. This refers to hospital admission required for treatment of the AE. This does not include "social or convenience" hospitalization for nonmedical causes such as lack of transportation to home; admissions of less than 24 hours for purposes of observation; confinement in, for example, a respite unit, a skilled nursing unit, or rehabilitation facility; or confinement due to a planned or an unplanned reason unrelated to the study. Emergency room visits that do not result in admission to the hospital should be evaluated for one of the other serious outcomes (e.g. life-threatening; required intervention to prevent permanent impairment or damage; other serious medically important event).
- Persistent or significant disability/incapacity or substantial disruption of the ability to conduct normal life functions
- Congenital anomaly/birth defect

Important medical events that may not result in death, be life threatening, or require hospitalization may be considered serious when, based upon appropriate medical judgment, they may jeopardize the subject and may require medical or surgical intervention to prevent one of the outcomes listed in this definition. Examples of such medical events include allergic bronchospasm requiring intensive treatment in an emergency room or at home, blood dyscrasias or convulsions that do not result in inpatient hospitalization, and the development of drug dependency or drug abuse.

Note that all SAEs are also AEs.

6.1.1.2. Unexpected Adverse Event or Serious Adverse Event

An AE or SAE is considered "unexpected" if it is not listed in the Investigators Brochure or is not listed at the specificity or severity that has been observed. For example, under this definition, hepatic necrosis would be unexpected (by virtue of greater severity) if the Investigator Brochure referred only to elevated hepatic enzymes or hepatitis. Similarly, cerebral thromboembolism and cerebral vasculitis would be unexpected (by virtue of greater specificity) if the Investigator's Brochure listed only cerebral vascular accidents. "Unexpected," as used in this definition, also refers to AEs that are mentioned in the Investigator Brochure as occurring with a class of drugs or as anticipated from the pharmacological

properties of the drug, but are not specifically mentioned as occurring with the particular drug under investigation.

6.1.1.3. Suspected Unexpected Serious Adverse Reaction

A SUSAR is an SAE that is unexpected and has a reasonable possibility of being caused by the drug (i.e., is an adverse reaction).

6.1.1.4. Severity of Adverse Events

The severity of AEs will be graded using the most current version of the Common Terminology Criteria for Adverse Events 5-point scale:

- Mild (grade 1): asymptomatic or mild symptoms: clinical or diagnostic observations only; intervention not indicated
- Moderate (grade 2): minimal, local or noninvasive intervention indicated; limited age-appropriate instrumental activities of daily living
- Severe (grade 3): severe or medically significant but not immediately life threatening: hospitalization or prolongation of hospitalization indicated; disabling; limiting self-care activities of daily living
- Life threatening (grade 4): life-threatening consequences; urgent intervention indicated
- Death (grade 5): death related to AE

It is emphasized that the term "severe" is a measure of severity; thus, a severe AE is not necessarily serious. For example, itching for several days may be rated as severe but may not be clinically serious.

6.1.1.5. Relationship of Adverse Events to Study Drug

The relationship of any AE to the study drug will be assessed and graded as related or not related. Adverse events will be considered "related" when there is a reasonable possibility that the drug caused the event. Adverse events will be considered "unrelated" when it appears very unlikely that the drug caused the event, such as when an alternate cause of the event is evident.

6.1.2. Recording and Reporting Adverse Events

6.1.2.1. Recording and Reporting of All Adverse Events

Adverse events will be recorded from the Baseline Visit (after first dose of study drug) until completion of the Follow-up Visit (at least 28 days after last dose of study drug). Any clinically significant observation, as assessed by the investigator, in clinical laboratory parameters, 12-lead ECG, echocardiogram, vital signs, or physical examinations may be recorded as an AE.

Adverse events will be elicited by asking the subject non-leading questions (e.g. "How do/did you feel?") at various times before and after study drug administration and at regular intervals throughout the study. Adverse events may also be spontaneously reported by the subject.

Subjects and parents/caregivers should be instructed to record AEs in the subject diary on a daily basis between visits. They should be told to report any untoward medical occurrence during the clinical study

from the time of first dose of study drug to the end study participation (at least 4 weeks after last dose of study drug).

During the defined AE collection period, the investigator will record all elicited and spontaneously reported AEs on the eCRF. The investigator will also transcribe any AEs recorded in the subject's paper diary on the eCRF. Each AE should be recorded to represent a single diagnosis. Accompanying signs or symptoms should not be recorded as additional AEs. If a diagnosis is unknown, signs and symptoms should be recorded as AEs.

The severity of AEs will be rated as mild, moderate, severe, life threatening, or death, and the relationship between the AE and the study drug will be indicated as not related or related. Other information to be collected for AEs include onset date, end date, frequency, seriousness, action taken, and outcome.

6.1.2.2. **Recording and Reporting of Serious Adverse Events**

All SAEs must be reported by the investigator to the sponsor or designee through Electronic Data Capture (EDC) system. Any SAE that occurs between the first dose of study drug until immediately prior to starting HSCT procedures, or 28 days after the last dose of study drug, if applicable, must be promptly (not later than 1 business day after the study site becomes aware of its occurrence) reported to the sponsor or designee. Scheduled hospitalization for initiation of HSCT procedures will not be considered an SAE. Any SAEs occurring more than 28 days after the last dose of study drug should be reported to the sponsor only if the investigator suspects a causal relationship to the study drug.

The initial SAE report should be submitted on the SAE eCRF and requires the following information (at a minimum):

- Subject identification number
- Reporter name/site number
- Adverse event term
- Suspect investigational product
- Relationship of the AE to study drug
- Criteria for seriousness

If there are any questions regarding the reporting of SAEs, the investigator should contact sponsor or designee. For protocol- or safety-related issues, the investigator should contact the Medical Monitor (specified in Contact details section).

6.1.3. **Regulatory Agencies, Institutional Review Board (IRB)/Independent Ethic Committees (IEC), and DSMB Reporting**

The sponsor or designee will submit all safety updates and periodic reports to the Food and Drug Administration (FDA), European Medicines Agency (EMA), and investigators in accordance with the FDA and EMA regulations.

For each SAE, the investigator and sponsor (or designee) will independently assess whether there is a reasonable possibility that the event may have been caused by the study drug (is "drug-related"). If the SAE is assessed to be both drug-related and unexpected, the sponsor or designee will report it to

the appropriate regulatory authorities and notify investigators as required by applicable local regulations.

The sponsor or designee will submit an expedited report of SUSARs to the FDA/EMA no later than 15 calendar days after the sponsor or designee first had knowledge of the adverse reaction. In fatal or life-threatening cases, a report will be submitted within 7 calendar days, and a complete report will be submitted within another 8 days.

It is the responsibility of the investigator to promptly notify the site's IRB/IEC of any Investigational New Drug Application (IND) safety reports or other matters involving risk to subjects as mandated by the IRB/IEC.

The sponsor will provide the DSMB with data on all SAEs on an ongoing basis.

6.1.4. Follow-up of Adverse Events

Any AE will be followed until the event returns to baseline or becomes stable with no further change expected. In the event of an abnormality considered to be clinically significant by the investigating physician, subjects will be followed with appropriate medical management until values are considered clinically acceptable and no further change is expected; referral or collaborative care will be arranged if required.

All SAEs should be monitored until they have resolved or stabilized.

6.2. Drug-Induced Liver Injury Management

6.2.1. Introduction

Transaminase increases combined with total bilirubin (TBIL) increases following MIN-102 exposure may be indicative of DILI and should be considered clinically important events. Laboratory parameters indicating potential DILI will be monitored according to the FDA Guidance "Guidance for Industry – Drug Induced Liver Injury".

6.2.2. DILI Monitoring Schedule

Monitoring for DILI will include tests for total bilirubin (TBIL, alkaline phosphatase, aspartate transaminase (AST), alanine transaminase (ALT), and prothrombin time at all the post-Baseline visits. For patients whose body weight is lower than 13.75Kg (30.31 Lbs.), prothrombin time will only be analyzed if the rest of the liver parameters show alterations suspicious of DILI.

6.2.3. DILI Follow-up

Detection:

For elevations in transaminases or bilirubin (AST or ALT > 3.0 x ULN combined with TBIL > 2.0 x ULN) in the absence of cholestasis (serum alkaline phosphatase < 2 x upper limit of normal (ULN) and no clinical evidence of biliary obstruction), the investigator will immediately request that the subject attend an unscheduled visit within 48 hours. In case close observation cannot be performed, study drug will be interrupted. If symptoms persist or repeat testing does not show a clinically significant reduction in levels of transaminase, INR, and/or bilirubin, a close observation will be initiated to determine whether the abnormalities are improving or worsening, and study drug will be interrupted.

Close observation will include:

- Repeating liver enzyme and serum bilirubin tests two times per week. Frequency of retesting can decrease to once a week if abnormalities stabilize and the subject is asymptomatic. Additional laboratories (such as fractionated bilirubin, other chemistries, and complete blood count [CBC]) may be ordered as necessary to more fully assess the subject's clinical status and/or the cause or effects of the hepatic abnormalities.
- Obtaining a more detailed history of symptoms and prior or concurrent diseases.
- Obtaining a history of concomitant drug use (including nonprescription medications and herbal and dietary supplement preparations), alcohol use, recreational drug use, and special diets in addition to exposure to environmental chemical agents.
- Further testing for acute hepatitis A, B, C, or E infection, other hepatotropic viral infection (cytomegalovirus, Epstein-Barr, or herpes simplex) or autoimmune hepatitis may be ordered if needed to clarify or confirm the cause of the liver function test abnormalities. Additional testing such as liver imaging or liver biopsy may be considered as clinically indicated or after consultation with specialist/hepatologist.

Additionally, study drug must be immediately discontinued in the following situations and subjects must be followed until full resolution:

- ALT or AST > 8 x ULN
- ALT or AST > 5 x ULN for more than 2 weeks
- ALT or AST > 3 x ULN and total bilirubin > 2 x ULN or INR > 1.5
- ALT or AST >3 x ULN with the appearance of fatigue, nausea, vomiting, right upper quadrant pain or tenderness, fever, rash, or eosinophilia (> 5%)

Follow-Up to Resolution

All trial subjects showing possible DILI should be followed until all abnormalities return to normal or to the baseline state.

6.2.4. Communication Flow

In this protocol, a central lab is used. Investigators should review all results issued by the central laboratory in an ongoing basis.

In case liver function test results are abnormal, the investigator will inform the subject that an unscheduled visit should be scheduled, at site if possible, as soon as possible, preferably within 48 hours. If the subject lives far away from the trial site, the investigator will inform the home nurse to obtain another blood sample. The following measurements will be performed at this unscheduled visit: TBIL, alkaline phosphatase, AST, ALT, and INR, as well as other tests (e.g. additional chemistries and CBC) deemed necessary by the investigator to more fully assess the subject's clinical status and/or the cause or effects of the hepatic abnormalities. Results will be made available to the investigator to be evaluated as soon as possible.

The investigator should communicate any events that meet the DILI as a serious adverse event (SAE) in 24 hours, as per procedures described in the protocol in section 6.1. All complementary tests

performed should be explained. Any confirmed DILI events will be reported to Competent Authorities as SUSARs as described in the protocol in Section 6.1.1.3.

All laboratory results analyzed by the central laboratory will be available in the database. All information regarding a potential DILI event will be recorded in the SAE forms and will be included in Suspect Adverse Reaction Report CIOMs form.

6.3. Detailed blood sampling volumes

Table 8. Total Blood sampling volumes per visit per patient

Assessment			Volume per sample	V-1	V0	V1	V2	V3 (8 weeks)	V4	V5	V6	V7	V8, 9, 10, 11, 12…	Visit prior to HSCT	FUV
**MIN-102 in plasma**			**1 mL**		5		2				2		1	1	
**Biomarkers**			**2 mL**		2		2				2		2	2	
**Clinical laboratory tests**	Blood chemistry	Cortisol, ACTH, aldosterone	**6 mL**	6											
		Hemoglobin A1c	**1 mL**	1										1	1
		Total bilirubin, AP, AST, ALT, gamma glutamyl transferase, lactate dehydrogenase, creatinine, urea, uric acid, total protein, albumin, glucose, inorganic phosphate, sodium, potassium, calcium, and chloride[1]	**2 mL**	2		2	2	2	2	2	2	2	2	2	2
		NT-proB-type Natriuretic Peptide (NT-proBNP)	**2 mL**		2		2				2		2	2	2
	Hematology		**1 mL**	1			1				1		1	1	1
	Prothrombin time[2]		**2 mL**		2	2	2	2	2	2	2	2	2	2	2
**Total volume of blood sampled**				**10**	**11**	**4**	**11**	**4**	**4**	**4**	**11**	**4**	**10**	**11**	**8**

[1] At Visits 1, 3, 4, 5 and 7 only total bilirubin, AP, AST and ALT will be analyzed.

[2] For patients whose body weight is lower than 13.75Kg (30.31 Lbs.), blood samples for prothrombin time analysis will only be drawn if liver parameters show alterations suspicious of DILI.

6.4. Neurological Functional Score – Major Functional Disabilities (NFS-MFDs) scale

nexus
A clinical study in cALD

NFS - MFDs
(Neurological Function Score – Major Functional Disabilities)

SUBJECT ID ____________ VISIT Nº __________

RATER ______________ Date [|] [|] [| | |]
MM DD YYYY

Symptoms/neuroexam	Score	
Hearing/auditory processing problems	1	☐
Aphasia/apraxia	1	☐
Loss of communication (MFD)	3	☐
Vision impairment/fields cut	1	☐
Cortical blindness (MFD)	2	☐
Swallowing/other CNS dysfunctions	2	☐
Tube feeding (MFD)	2	☐
Running difficulties/hyperreflexia	1	☐
Walking difficulties/spasticity/spastic gait (no assistance)	1	☐
Spastic gait (needs assistance)	2	☐
Wheelchair dependence (MFD)	2	☐
No voluntary movement (MFD)	3	☐
Episodes of incontinency	1	☐
Total incontinency (MFD)	2	☐
Nonfebrile seizures	1	☐

Total NFS: [Auto-calculated in eCRF]

Nº MFD: [ ]

Moser, H.W., Loes, D.J., Melhem, E.R., Raymond, G.V., Bezman, L., Cox, C.S., and Lu, S.E. (2000). X-Linked adrenoleukodystrophy: overview and prognosis as a function of age and brain magnetic resonance imaging abnormality. A study involving 372 patients. Neuropediatrics 31, 227–239

NFS-MFD scoresheet version 2.0 – 24 October 2019

6.5. NFS scale item definitions for standardized rating

MT-2-02
NFS-MFD rating rules
Version 2.0 –15 Jan 2020

	impairment is minimal and does not impair ambulation. Activities can still be carried out without or with minimal restrictions, either without aid or with minimal assistance. Hyperreflexia may be present.	• New onset of hyperreflexia (3 and 4+ deep tendon reflexes or ankle clonus).<br>• Child can still walk/ambulate with minimal assistance which does not limit activity level (daycare or school activities, navigating home environment and playground play).
**Walking difficulties / spasticity / spastic gait (no assistance)**	Subject shows spasticity with spastic gait but is still ambulatory without aid. Walking difficulties impair usual daily activities.	• New difficulty walking due to spasticity that limits access or ability to complete previous daily activities (daycare or school activities, navigating home environment and playground play)<br>• Evaluate gait during encounter.<br>• No need of walking aid such as crutches or walker (braces/AFO walking boots do not count).
**Spastic gait (needs assistance)**	Subject needs constant bilateral walking aids.	Child needs constant bilateral walking aids such as crutches or walker.
**Wheelchair dependence (MFD)**	Subject cannot walk more than few steps but requires a wheelchair to get around. Subject can move the wheelchair by him/herself but may need assistance for longer distances or motorized wheelchair to maintain full range of daily activities.	• Wheelchair is needed for all activities (not due to fatigue but true inability to ambulate).<br>• Child still can move arms and legs (maybe even able to do few steps with full assistance) and is able to move wheelchair for short distances.
**No voluntary movements (MFD)**	Subject is unable to perform simple voluntary activities with arms or legs.<br><br>*Note that isolated random, but non-purposeful movements can occur*	• Wheelchair or bed bound with no voluntary movement of arms and legs<br>• Reflexive limb movements maybe present
**Episodes of incontinence**	Subjects without previous incontinence for at least 6 months presents with frequent episodes of urge or incontinence during the day or night, or with bowel or bladder retention.	• Only applicable to children who have completed toilet training and have none or sporadic episodes of incontinence over previous 6 months.<br>• New episodes of urine or stool incontinence during the day or night compared with 6 months period prior capacity.
**Total incontinence (MFD)**	Subject without previous incontinence shows permanent and complete loss of control over bladder and/or bowel.	• Only applicable to children who have completed toilet training.<br>• Permanent and complete bladder and/or bowel incontinence.
**Nonfebrile seizures**	Subject develops non-febrile seizure	Includes partial or generalized seizures that occur in the absence of fever (>24 hours apart from febrile episode)

	impairment is minimal and does not impair ambulation. Activities can still be carried out without or with minimal restrictions, either without aid or with minimal assistance. Hyperreflexia may be present.	• New onset of hyperreflexia (3 and 4+ deep tendon reflexes or ankle clonus).<br>• Child can still walk/ambulate with minimal assistance which does not limit activity level (daycare or school activities, navigating home environment and playground play).
**Walking difficulties / spasticity / spastic gait (no assistance)**	Subject shows spasticity with spastic gait but is still ambulatory without aid. Walking difficulties impair usual daily activities.	• New difficulty walking due to spasticity that limits access or ability to complete previous daily activities (daycare or school activities, navigating home environment and playground play)<br>• Evaluate gait during encounter.<br>• No need of walking aid such as crutches or walker (braces/AFO walking boots do not count).
**Spastic gait (needs assistance)**	Subject needs constant bilateral walking aids.	Child needs constant bilateral walking aids such as crutches or walker.
**Wheelchair dependence (MFD)**	Subject cannot walk more than few steps but requires a wheelchair to get around. Subject can move the wheelchair by him/herself but may need assistance for longer distances or motorized wheelchair to maintain full range of daily activities.	• Wheelchair is needed for all activities (not due to fatigue but true inability to ambulate).<br>• Child still can move arms and legs (maybe even able to do few steps with full assistance) and is able to move wheelchair for short distances.
**No voluntary movements (MFD)**	Subject is unable to perform simple voluntary activities with arms or legs.<br><br>*Note that isolated random, but non-purposeful movements can occur*	• Wheelchair or bed bound with no voluntary movement of arms and legs<br>• Reflexive limb movements maybe present
**Episodes of incontinence**	Subjects without previous incontinence for at least 6 months presents with frequent episodes of urge or incontinence during the day or night, or with bowel or bladder retention.	• Only applicable to children who have completed toilet training and have none or sporadic episodes of incontinence over previous 6 months.<br>• New episodes of urine or stool incontinence during the day or night compared with 6 months period prior capacity.
**Total incontinence (MFD)**	Subject without previous incontinence shows permanent and complete loss of control over bladder and/or bowel.	• Only applicable to children who have completed toilet training.<br>• Permanent and complete bladder and/or bowel incontinence.
**Nonfebrile seizures**	Subject develops non-febrile seizure	Includes partial or generalized seizures that occur in the absence of fever (>24 hours apart from febrile episode)

6.6. Rationale of the amendment

Amendment leading to Protocol v7.0 dated 24 April 2024

This amendment is an administrative change to generate a consolidated Protocol version to adapt to new regulation in Europe (CTIS transition).

Minor non-substantial changes have been included.

Amendment leading to Protocol v6.1 dated 26 Sep 2022/Protocol v6.2 dated 26 Sep 2022.

This amendment is an administrative change of Pharmacovigilance vendor. It also contains minor changes in PK parameters to be determined (apparent clearance will not be determined as it cannot be estimated reliably with the current design).

Amendment leading to Protocol v5.1 dated 17 Nov 2021

This amendment is mainly driven by the availability of new natural history data which has provided a better understanding of the natural course of the disease in this population as well as a further knowledge of the disease evolution under the current Standard of Care (SoC).

The amendment revolves around the following three main points:

1. The study primary objective has been more strictly defined according to the recent natural history and how disease evolves under the SoC

2. The study "end of treatment criteria" have been aligned accordingly to the mentioned current evidence

3. One inclusion criteria has been widened to allow more patients participating in the study

1. Primary objective

The concept of "arrested disease" accounts for two types of assessments:

- Clinical symptoms related with cerebral function (assessed by NFS & MFD) and
- Brain MRI assessments (Gadolinium Intensity Score (GIS) & lesion growth assessed by T2/FLAIR).

In terms of clinical assessment (NFS & MFD) the definition of "arrested disease" does not change. However, regarding the MRI assessments, the most recent and extensive natural history studies in ALD patients presenting cerebral lesions (Mallack et al., 2020 and 2021) consider cALD as "arrested disease" if assessed "≥2 consecutive MRIs spanning a minimum of 6 months with no disease progression on MRI, no contrast enhancement and no progression of cerebral symptoms". Additionally, patients may show fluctuations between Gd-positive and Gd-negative lesions, indicating that lesion progression can be better characterized by change in lesion volume rather than by presence of Gd enhancement (Mallack et al., 2021).

Therefore, the definition based on MRI assessment has been updated according to these publications, as described below.

For "lack of disease progression" (assessed by MRI) to be considered, patients are now required to fulfill both the defined criteria instead of either of them individually (i.e., no contrast enhancement, AND no significant growth of lesions) due to the published data supporting a better predictive value when

assessing lesion growth/volume. This is resulting in a stricter assessment. Hence, both of the following criteria need to be fulfilled:

- No contrast enhancement present is defined by GIS=0 instead of GIS ≤1 and Gd enhancement has to persist in 2 consecutive MRIs (persistent Gd-positive lesion) to consider that lesion is progressing to account for any potential fluctuation. This resulting in a stricter assessment.
- No significant growth of T2/FLAIR lesions is compared to the previous MRI to account for the fact after SoC there may be an initial period of growth with lesion deceleration, followed by lesion stabilization.

2. Study end-of-treatment criteria

In addition, it has been recently reported that patients treated under current SoC, i.e. HSCT (Cartier and Aubourg, 2010 and Raymond et al., 2019), or with gene therapy (Eichler et al., 2017), may need up to 18 months after transplantation to stabilize lesion progression, while, lesion growth deceleration is usually observed during this period (Eichler et al., 2017 and Mallack et al., 2019). Therefore, having a study "End of Treatment criteria" at week 24 only based on the "arrested disease" definition described above would not be aligned with the current evidence on lesion dynamics from the SoC, as patients with decelerating lesion progression would have not been eligible for treatment anymore. Therefore, with the proposed protocol amendment v5.1, a "continuation criteria" has been added allowing the study to continue at week 24 if lesion growth deceleration is observed by central readers, hence continuing the follow up of these patients who may potentially benefit of longer-term treatment.

3. Inclusion criteria

Inclusion Criteria #6 has been expanded to include patients with GIS scores up to 3. Therefore, the proposed design is now more inclusive by allowing this more affected patient population in the study, hence, potentially conferring benefit to a broader population.

A comprehensive list of the changes can be found in section 6.7.

<u>Amendment leading to Protocol v4.1 dated 10 June 2021</u>

The rationale for this amendment is to remove the exclusion criterion #7 (in former current protocol version 3.2 dated 28 May 2020): *Current use of immunosuppressant medication, excluding corticosteroids* in addition of removing the use of immunosuppressant medication from the prohibited medication. This criterion and the prohibited use of corticosteroids during the study was initially introduced preventively based on concerns over potential hemodilution effects of MIN-102 that could aggravate neutropenia and anemia that have been described for immunosuppressants. However, available safety data from completed and ongoing clinical studies with a total of 141 patients exposed for up to 3 years have shown that the reports blood and lymphatic system adverse events are low, can be explained by hemodilution, are generally mild, recovered in short term and no actions regarding treatment had to be taken. It was therefore concluded that the risk of further lowering white and red blood cell counts is very low. In addition, long-term toxicology studies have not revealed evidence of myelosuppressive effects of MIN-102.

Further exclusion criteria to address the safety of patients, such as #14 "Clinically significant anemia with hemoglobin <10 g/dL" remain in place. In case patients are taking concomitant immunosuppressants, the investigator has to observe the warnings and precautions per the prescribing information of the respective immunosuppressants.

In addition, some prohibited medication will be removed from the list based on the following: The risk assessment of pharmacokinetic (PK) drug-drug interactions (DDI) has been re-evaluated based on a physiologically-based PK (PBPK) model which ruled out the potential risk for MIN-102 when co-administered with OATP1B1 and OATP1B3 substrates, MDR1 and BCRP inhibitors and CYP12A substrates. Also, as MIN-102 is predominantly metabolized by CYP2C8 and CYP3A4 (≥25%), inducers of these enzymes could reduce the efficacy of MIN-102 and thus the CYP3A inducer rifampicin should be included in the prohibited co-med list.

6.7. Summary of changes

Sections Changed	Description of Change	Reason for Change
Header and Cover page	Update versioning to Version 7.0, 24 April 2024	To create a consolidated protocol for CTIS transition.
Header and Cover page	EU CT number added: **2024-513774-21-00**	For CTIS transition: EU number created in CTIS system.
AUTHORIZATION OF CLINICAL STUDY PROTOCOL	Added CMO as a Sponsor Signatory	Added as per updated Minoryx SOPs from last approved version.
CONTACT INFORMATION	• Sílvia Pascual's role updated<br>• Sponsor's medical Expert contact updated<br>• CRO Global Study Manager contact information removed.<br>• Biomarkers lab name updated	Administrative changes.
SYNOPSIS<br><br>Sections 3.1 (specially sub-section 3.1.2)<br><br>Section 3.3 (subsection 3.3.2)	The following 2 End-of-treatment criteria are specific for France upon request during approval process (section has been updated as non substantial change as it was not referring to the proper section):<br><br>*8.* ***Subjects do not tolerate the minimum allowed dose (see section 3.4.5)***<br><br>9.***Subjects show clinically significant out-of-range laboratory values, clinically significant abnormal findings on physical examination, or intolerable adverse events (AEs) that put him at additional risk, as judged by the investigator. For termination of treatment in case of drug induced liver injury (DILI) refer to section 6.2***.<br><br>The following 4 Exclusion criteria are specific of France protocol:<br><br>***9. Chronic or recurrent symptomatic urinary infections (≥2 per year over the past 2 years until Screening [V-1]).***<br><br>***10. Permanent indwelling urinary catheter or catheter port***<br><br>***11. Smoking with 25 cigarettes per day over the past 2 years until Screening (V-1)***<br><br>***21. A positive result on laboratory***<br><br>***tests for hepatitis B surface antigen, hepatitis C antibody or human immunodeficiency virus antibody***<br><br>In the Flow chart of study procedures the following procedure is specific only for France only at Screening so no longer applicable as recruitment is closed:<br>***Virology testing for hepatitis B, hepatitis C and human immunodeficiency virus (HIV)***	To consolidate the 2 approved protocol versions 6.1 in Spain and Germany and 6.2 in France.<br><br>The main differences are:<br>- some specific exclusion criteria in France that are no longer applicable since the recruitment is closed.<br>- 2 specific end-of-treatment criteria requested during the approval process in France<br>- Virology testing at screening applicable only in France.

Sections Changed	Description of Change	Reason for Change
Non-substantial Changes<br>Miscellaneous	In 3.1 section:<br>• in the current approved protocol version 6.2 it said: "to achieve a target plasma exposure of 170 µg•hr/mL" but in this consolidated protocol a non- substantial change has been done to match the Protocol version 6.1: "to achieve an exposure of approximately 170 µg•hr/mL".<br>• End-of-treatment criteria described in the approved protocol 6.1 is now moved as in the protocol 6.2 in section 3.1.2.<br>In section 3.3:<br>• A non-substantial change from approved Protocol version 6.2 in France is made: instead of "dropping out", "stopping treatment"<br>In section 3.4.2:<br>• Posoloy wording: in approved version 6.1 it says: "*Once-daily dosing with a volume specified by the pharmacokinetic specialist to achieve the **target** plasma exposure (170 µg.hr.mL-1)*" in protocol version 6.2 it says: "*Once-daily dosing with a volume specified by the pharmacokinetic specialist to achieve the **desired** plasma exposure (170 µg.hr.mL-1)*" For consistency with it is left as "target".<br>In section 3.4.3:<br>• An individualized ***starting*** dose based on PBPK will be chosen to yield a geometric mean AUCt of approximately 170 µg•hr/mL with an expected standard deviation of approximately 20%. in the French version 6.2 "starting" was not present, for the sake of consistency it has been added.<br>• The model will be updated and adjusted, if needed, with PK results obtained during the ongoing study. Additional blood samples to determine MIN-102 plasma concentration will be taken during the study at all on-site visits. Further dose adjustments to achieve the target AUC of 170 µg•hr/mL **± 20%** may be made at any time during the study if deemed necessary. The range of 20% was missing in the version 6.1, updated for consistency.<br>In section 3.5.3.2:<br>• Last 2 paragraphs of the section of Protocol version 6.1 has been eliminated for the sake of consistency and can be found are replaced by the updated section 3.1.2.3 in this consolidated protocol.<br><br>In section 3.6.1.3 and section 3.6.2:<br>• Protocol version 6.2 had the wording corresponding to protocol version 4.2, not consistent with Synopsis and section 2 (objectives) so it has been updated to match the approved version 6.1.<br>In section 3.6.3:<br>• According to protocol version 6.2 "Decisions regarding subject replacement will be documented. Patients who drop out as treatment failures defined as patients meeting "HSCT	For the sake of consistency in this consolidated protocol.

Sections Changed	Description of Change	Reason for Change
	criteria", shortlisted for HSCT for overall lesion progression in the judgment of the investigator, or not meeting "short-term arrested disease" criteria will not be replaced. However, patients who drop out by withdrawal of consent by parents or termination of treatment by the investigator for non-treatment related causes may be replaced. Patients dropping out will be encouraged to remain in the study and attend all further scheduled efficacy and safety assessments." For the sake of consistency the the paragraph used in approved Protocol v6.1 is left and is considered a non substantial change.	
