# Supplementary material for: Safety and efficacy of leriglitazone in childhood cerebral adrenoleukodystrophy (NEXUS): an interim analysis of an open-label, phase 2/3 trial
Source: eClinicalMedicine. 2025 May 24;84:103265. doi: 10.1016/j.eclinm.2025.103265 (PMC12159931; doi:10.1016/j.eclinm.2025.103265)
Supplement: Signed Final.pdf [file mmc3.pdf]

OPEN-LABEL, MULTICENTER STUDY IN MALE PEDIATRIC PATIENTS WITH CEREBRAL X-LINKED ADRENOLEUKODYSTROPHY (CALD) TO ASSESS THE EFFECTS OF MIN-102 TREATMENT ON DISEASE PROGRESSION PRIOR TO HUMAN STEM CELL TRANSPLANT (HSCT)

Protocol Number:	MT-2-02
Protocol Version:	5.1,5.2
Protocol Date:	17 Nov 2021

STATISTICAL ANALYSIS PLAN

Version 3.0

Open-Label, Multicenter Study in Male Pediatric Patients With Cerebral X-Linked Adrenoleukodystrophy (cALD) to Assess the Effects of MIN-102 Treatment on Disease Progression Prior to Human STEM Cell Transplant (HSCT)

STATISTICAL ANALYSIS PLAN

Version 3.0

Author:

Seth Bennett
Biostatistician II, CTI

DocuSigned by:
Seth Bennett
Signer Name: Seth Bennett
Signing Reason: I am the author of this document
Signing Time: 21-Oct-2022 | 6:20:03 AM EDT
1D9E9FC1398F45089B4F8325E01BB911

CTI Biostatistics Reviewer:

David McCollum, Director, Global Biostatistics, signing on behalf of Rachael Gilbert Runyan

Rachael Gilbert Runyan
Assistant Director, Biostatistics, CTI

DocuSigned by:
David McCollum
Signer Name: David McCollum
Signing Reason: I have reviewed this document
Signing Time: 21-Oct-2022 | 8:12:37 AM EDT
D431E5EDB26F4E75A11AD083AEFDB1F6

Sponsor Approval: Marc Martinell
Chief Executive Officer
Minoryx Therapeutics S.L.

DocuSigned by:
Marc Martinell
Signer Name: Marc Martinell
Signing Reason: I approve this document
Signing Time: 21-Oct-2022 | 3:07:56 AM PDT
239648531BDF4A4F9D8E53BE94F0FF8B

SAP Revisions

Version 1.0 of the SAP was finalized at the time of Protocol version 2.0 (06AUG2019). The following table details the changes made to the SAP due to the change in Protocol version 2.1.

Protocol Version # Date	SAP Section	Modification	Description and Rationale
2.1 19AUG2019	5.3	Removed: Gender	This was done since this is a study for boys only.
2.1 19AUG2019	6	Changed: "A comparison of MRI study results (T2/FLAIR from primary endpoint, all other secondary and exploratory MRI endpoints of FA and ADC on DTI) will be performed with a separate sample of patients derived from the database at the University of Minnesota." to "A comparison of MRI study results for white matter T2/FLAIR hyperintensity lesion volume, and white matter fiber structure as determined by diffusion tensor imaging (DTI) will be performed with a separate sample of patients derived from the database at the University of Minnesota."	Updated to match protocol.
2.1 19AUG2019	6.3	Added: "Change from Baseline in T2/FLAIR lesion volume"<br>Removed: ", and may be compared to the University of Minnesota data referred to in the Protocol where possible"	Updated to match protocol.
2.1 19AUG2019	6.4	Removed: "maximum plasma concentration"	This was done to align with the acronyms used in the protocol.
2.1 19AUG2019	7.2.1	Added: reference for PV	Updated to match protocol.
2.1	7.5	Added: reference for Fridericia	Updated to match protocol.

Protocol Version # Date	SAP Section	Modification	Description and Rationale
19AUG2019			
5.1 17Nov2021	1	Added extract of the Protocol's introduction	Updated to match protocol
5.1 17Nov2021	2.2.2	New section to capture interim evaluation	To include a section for Week 24 Interim Evaluation
5.1	3.1	Changed "GIS of 2 or 3" to "GIS of 1, 2 or 3"<br><br>Added "If treatment is stopped, all assessments equivalent to the "Visit Prior to HSCT" will be performed one day after last dose of study drug. The Follow-up Visit will be scheduled 4 weeks (±5 days) after last dose of study drug."	Updated to match protocol
17Nov2021	3.2.2	Updated end-of-treatment criteria	Updated to match protocol
5.1	4	Included information on method for confidence Interval	
	4.1	Updated whole section	To capture current analysis quality control
	4.2.2	Updated definition for mITT	To clearly define populations 1 and 2
	4.2.4	Added definition for PK Analysis Set	Updated to match protocol
	4.2.5	Added definition for PD Analysis Set	Updated to match protocol
	4.3	Eligibility for Efficacy Analysis	Distinguish between "evaluable" and "non-evaluable"
	4.4	Updated HSCT visit to myoablative condition	To match how data is collected
	4.5	Added text for multiple comparison	To match Week 96 analyses
	4.6.1	Previous section 4.6	No change

Protocol Version # Date	SAP Section	Modification	Description and Rationale
	4.6.2	Added section for Drug Compliance	Additional formula update
	4.6.3	Added section for deriving predicted AUC	Additional formula update
	4.6.4	New section for data review meeting	To access individual data into the appropriate population
	5.1	Updated text for disposition analyses	Clarify data to summarize
	5.2	Updated closure to lock.<br>Added "A listing of protocol deviations will be provided and will include type of protocol deviation, the action taken, date it occurred and whether it is a major or minor deviation for SAS"	To indicate what will be presented
	5.3	Added "for SAS"	Specify population for analysis
	5.4	Added a section for Medical History	Needed for final analysis
	5.5	Update title to "Prior and Concomitant Medication"<br>Added WHO drug version<br>Updated "based on" to "for"<br>Updated "patient" to "patient"<br>Added for SAS	To capture prior medications
	6	Updated "made" to "provided"<br>Removed "Outcome variables will be evaluated for change from baseline to the designated time point using descriptive statistics (n, mean, SD, median, minimum, and maximum). In addition, categorical outcomes such as first appearance of Gd-enhancing cerebral lesions in	To clarify efficacy analyses

Protocol Version # Date	SAP Section	Modification	Description and Rationale
		patients not showing these lesions at baseline will be analyzed using frequencies tables”<br><br>Added “A comparison of MRI parameters and also of clinical endpoints will be performed in those instances where there is available natural history data of sufficient quality to make such comparison. The primary comparison to the natural history will be performed with the most up-to-date and comparable data available. Natural history data generated by Mallack et al. (2021) or later publications will be the basis of the primary comparison as this data was generated with the same MRI protocol and highly comparable assessment through expert manual segmentation of brain lesions.”<br><br>All efficacy analysis will be performed using the mITT analysis set”	
	6.1	Added more information to explain definition of arrested disease.	Add clarity to primary endpoint analyses
	6.1.1	Added a section for Arrested Disease Criteria	To further clarify Arrested Disease
	6.1.1.1	Arrested Disease Criteria at Week 24	Define arrested disease at Week 24
	16.1.1.2	Arrested Disease Criteria at Week 96 or visit prior to HSCT	Define Arrested Disease at Week 96
	6.2	Added “including confidence intervals, and graphical	

Protocol Version # Date	SAP Section	Modification	Description and Rationale
		representations will be provided (mean values or proportions over time, or Kaplan-Meier curves in the case of time to event endpoints)"	
	6.3	Added "Each of the endpoints will be descriptively summarized, including confidence intervals, and graphical representations. Changes from baseline will be presented as relative change per month.<br>Additional calculations will be derived for normalized lesion volume, fold change from baseline, fold change from baseline per month, velocity, and acceleration. Each of this will be descriptively summarized and graphical representations to compare with Natural History data from literature."	Add clarity to exploratory analyses to be performed.
	6.4	Exploratory efficacy Endpoints and Analyses	Added to match exploratory objectives
	6.5	Included section for Week 24 Interim Evaluation	Explain analyses at Week 24 interim evaluation
	7	Updated section 6.5 to section 7 and Added additional PK parameters	Based on data collected during PK evaluation
	8	Updated section 6.6 to section 8 and Updated biomarkers analyzed.	To match Biomarkers analyzed
	9	Updated section title to "Safety and Tolerability"<br>Replaced "safety population" to "SAS".	
	9.1	Updated "as well as" to "including".	Add additional exposure data recorded

Protocol Version # Date	SAP Section	Modification	Description and Rationale
		Added "and duration of exposure"	
	9.2.2	Updated "drug, but" to "drug but"	Updated grammar
	9.2.6	Updated list of AE summaries	Based on sponsor request
	9.3	Updated title to include "Cytological"<br><br>Updated list of assessments for chemistry, hematology, prothrombin time, blood spot glucose check, urinalysis and cytological examination.	To match protocol update.
	9.4	Added "Patients can be contacted by phone between scheduled visits and be advised to monitor their weight frequently and report back to the investigator in case of weight gain. If required, an unscheduled visit may be scheduled at any time".<br><br>Updated "data" to "values"<br><br>Added "A listing of all vital signs results will be presented for SAS"	
	9.6	Added "A listing of echocardiogram results including any abnormalities and the corresponding clinical significance will be presented"	
	9.7	Added "for SAS"<br><br>Added "A separate listing will be provided for edema assessment for SAS"	
	9.8	Replaced "descriptive statistics" with "counts and frequencies"<br><br>Added "at each visit"	

Protocol Version # Date	SAP Section	Modification	Description and Rationale
		Added "A listing of palatability assessment will also be provided for the SAS."	
	10	Removed "dropping out" and added "stopping treatment"	To match protocol
	11	Added additional references	To match all references in the SAP

Table of Contents

SAP REVISIONS....3
LIST OF ABBREVIATIONS AND TERMS....12
1. INTRODUCTION....13
2. OBJECTIVES AND ENDPOINTS....14
2.1 OBJECTIVES....14
2.1.1 *Primary Objective*....14
2.1.2 *Secondary Objectives*....14
2.1.3 *Exploratory Objective*....14
2.2 ENDPOINTS....14
2.2.1 *Primary Efficacy Endpoint*....14
2.2.2 *Secondary Efficacy Endpoints*....14
2.2.3 *Exploratory Efficacy Endpoints*....15
2.2.4 *Week 24 Interim Evaluation Endpoints*....15
3. INVESTIGATIONAL PLAN....15
3.1 STUDY DESIGN....15
3.2 TREATMENT....16
3.2.1 *Treatment Compliance*....17
3.2.2 *Duration of Treatment*....17
4. GENERAL CONSIDERATIONS FOR DATA ANALYSIS....17
4.1 ANALYSIS QUALITY CONTROL PROCEDURE....18
4.2 ANALYSIS SETS....18
4.2.1 *Safety Analysis Set (SAS)*....18
4.2.2 *Modified Intent-To-Treat Analysis (mITT) set*....18
4.2.3 *Per-Protocol Analysis Set (PPS)*....18
4.2.4 *Pharmacokinetic Analysis Set (PKS)*....19
4.2.5 *Pharmacodynamics Analysis Set (PDS)*....19
4.3 ELIGIBILITY FOR EFFICACY ANALYSIS....19
4.4 HANDLING OF DROPOUTS OR MISSING DATA....19
4.5 MULTIPLE COMPARISONS....20
4.6 DATA DERIVATIONS AND TRANSFORMATIONS....20
4.6.1 *Baseline and Change from Baseline*....20
4.6.2 *Data Review Meeting*....20
5. DEMOGRAPHIC AND BASELINE CHARACTERISTICS....20
5.1 DISPOSITION OF PATIENTS....20
5.2 PROTOCOL DEVIATIONS....20
5.3 DEMOGRAPHICS....21
5.4 MEDICAL HISTORY....21
5.5 PRIOR AND CONCOMITANT MEDICATIONS....21
6. EFFICACY ANALYSIS....21
6.1 KEY EFFICACY DEFINITIONS....22
6.1.1 *Arrested Disease Criteria*....22

6.1.1.1 Arrested Disease Criteria at Visit 8 (Week 24) 22
6.1.1.2 Arrested Disease Criteria at Visit 12 (Week 96) or Visit prior to HSCT 22
6.2 PRIMARY EFFICACY ENDPOINT ANALYSIS 22
6.3 SECONDARY EFFICACY ENDPOINTS AND ANALYSES 23
6.4 EXPLORATORY EFFICACY ENDPOINTS AND ANALYSES 23
6.5 WEEK 24 INTERIM EVALUATION 24
6.6 SENSITIVITY ANALYSES 24

7. PHARMACOKINETIC EVALUATION 25

8. BIOMARKER EVALUATION 25

9. SAFETY ANALYSIS 25

9.1 EXPOSURE 25
9.2 ADVERSE EVENTS 25
9.2.1 Serious Adverse Events 26
9.2.2 Severity of Adverse Events 26
9.2.3 Relationship of Adverse Events to Study Drug 27
9.2.4 Adverse Event Summaries 27
9.3 CYTOLOGICAL CLINICAL LABORATORY ASSESSMENTS 28
9.4 VITAL SIGNS 29
9.5 ELECTROCARDIOGRAM 29
9.6 ECHOCARDIOGRAM 29
9.7 PHYSICAL EXAMINATION 29
9.8 PALATABILITY ASSESSMENT 30

10. SAMPLE SIZE 30

11. REFERENCES 30

12. APPENDICES 32

12.1 APPENDIX A: STUDY DESIGN 32
12.2 APPENDIX B: FLOW CHART OF STUDY PROCEDURES 33

List of Abbreviations and Terms

Abbreviation	Definition
ACTH	adrenocorticotropic hormone
ADC	apparent diffusion coefficient
AE	adverse event
ALD	adrenoleukodystrophy
ALK	alkaline phosphatase
ALT	alanine aminotransferase
AST	aspartate aminotransferase
ATC	Anatomical, Therapeutic, Chemical
AUC	area under the concentration-time curve
BLQ	Below limit of quantification
cALD	cerebral adrenoleukodystrophy
C2h	plasma concentration at 2 hours
C_{min}	minimum plasma concentration
CI	Confidence Interval
CSF	cerebrospinal fluid
CHT	Capillary Mean Transient Time Heterogeneity
DILI	drug-induced liver injury
DSC	dynamic susceptibility contrast
DTI	diffusion tensor imaging
ECG	electrocardiogram
eCRF	electronic case report form
FA	fractional anisotropy
FLAIR	fluid attenuation inversion recovery
FUV	Follow-up visit
Gd	gadolinium
GIS	gadolinium intensity score
HbA1c	glycated hemoglobin
HSCT	hematopoietic stem cell transplantation or transplant
ICH	International Conference on Harmonization
IL	interleukin
INR	International Normalized Ratio
Kapp	Constant of gadolinium permeability using advance perfusion software

Abbreviation	Definition
K2	Constant of gadolinium permeability using conventional perfusion software
MCP-1	monocyte chemoattractant protein-1
MFD	Major Functional Disabilities
mITT	modified intent-to-treat
MRI	magnetic resonance imaging
NFS	Neurological Function Scale
NfL	Neurofilament light chain
NT-proBNP	NT-proB-type Natriuretic Peptide
PBPK	physiologically based PK
PD	pharmacodynamic
PK	pharmacokinetic or pharmacokinetics
PP	per-protocol
PT	preferred term
QTcF	QT interval corrected for heart rate using Fridericia’s formula
SAE	serious adverse event
SAS	safety analysis set
SAP	Statistical analysis plan
SD	standard deviation
SOC	system organ class
ULN	upper limit of normal
TBIL	total bilirubin
WHO	World Health Organization

1. INTRODUCTION

This is an open-label, multicenter study in male pediatric patients, aged ≥2 and ≤12 years, with a diagnosis of the cALD phenotype of X-linked ALD. This study will provide an assessment of the effects of MIN-102 on cALD disease progression prior to HSCT, the only therapeutic intervention currently available to cALD patients, or experimental ex-vivo autologous stem cell transplant.

This document details the Statistical Analysis Plan (SAP) for study protocol MT-2-02.

The SAP will be finalized before database closure for the final data analysis after the completion of study visits. Any deviations from the analysis pre-planned in the protocol or the SAP will be described and justified in the final study report.

This study will be conducted in compliance with the study protocol and ICH guidelines E9 (Statistical Principles for Clinical Trials 1998).

"Arrested disease" will be assessed at two timepoints:

- 24 weeks after start of treatment (Visit 8), and
- 96 weeks after start of treatment (Visit 12)

The first assessment will be conducted when up to 13 evaluable patients have reached 24 weeks of treatment. The second timepoint represents the confirmatory timepoint.

"Arrested disease" is defined using the following parameters:

- Change in Neurological Function Score (NFS) from Baseline ≤1 point (Week 24) or ≤5 points (Week 96)
- Free of MFD
- Lack of lesion progression on MRI, fulfilling the following:
  - No conversion to Gd+ lesions (Population 1) defined as GIS score of 0
  - Disappearance of persistent Gd+ lesions (Population 2) defined as change in GIS score from 1, 2 or 3 to 0. Persistent Gd+ lesions is defined as those present in ≥ 2 consecutive MRIs spanning a minimum of 6 months.
  - No significant growth of T2/FLAIR lesions compared to the previous MRI (both Populations) as assessed by central reading

The study will be continued if a minimum of 4 of 13 evaluable patients have met the endpoint of "arrested disease" at Week 24 or if the overall assessment from central reading suggests lesion growth deceleration ("continuation criteria"). Lesion deceleration shall be considered when lesion growth determined by central readers assessment is below what would be expected based on the literature (Liberato et al., 2019, Mallack et al., 2019, Mallack et al., 2020, Mallack et al., 2021) and/or when lesion growth rate of the latest measure is lower than previous ones. Growth rate is defined as the increase in T2/FLAIR lesion volume since previous MRI divided by the number of months elapsed since that previous MRI.

The study will be considered successful if a minimum of 4 patients met all "arrested disease" criteria at Week 96, except for MFD where >70% of all evaluable patients at Week 96 have to be free of MFD.

Clarification note: The > 70% condition is to be applied to the number of patients who meet all

"arrested disease" criteria including being free of MFD as a percentage of those who meet all "arrested disease" criteria not including being free of MFD.

2. OBJECTIVES AND ENDPOINTS

2.1 Objectives

2.1.1 Primary Objective

The primary objective of the study is to evaluate whether MIN-102 can halt progression of cALD at week 96 as determined by serial clinical and magnetic resonance imaging (MRI) investigations in pediatric patients.

2.1.2 Secondary Objectives

The secondary objectives of the study are as follows:

- To assess the changes in neurological function.
- To evaluate the effects of pre-HSCT MIN-102 treatment on:
  - Loes scores
  - Gadolinium intensity score (GIS)
  - Overall survival of patients who have not undergone HSCT
  - Number of patients meeting HSCT criteria
- To assess the pharmacokinetics (PK), safety, tolerability, and palatability of MIN-102 in pediatric patients

2.1.3 Exploratory Objective

To evaluate the effects of MIN-102 treatment on:

- White matter T2/FLAIR hyperintensity lesion volume
- White matter fiber structure as determined by diffusion tensor imaging (DTI)
- Cerebral blood flow, blood volume, capillary mean transient time heterogeneity (CHT) and constants of permeability (K2 and Kapp) as determined by DSC MRI
- Plasma biomarkers and cerebrospinal fluid (CSF) biomarkers (optional)

2.2 Endpoints

2.2.1 Primary Efficacy Endpoint

The primary efficacy endpoint will be the number of patients meeting "arrested disease" criteria at Visit 12 (Week 96).

Refer to Section 6.1.1.2 for the definition of "arrested disease."

2.2.2 Secondary Efficacy Endpoints

The secondary efficacy endpoints will be:

- Sustained change from Baseline in the score composed of NFS items 1, (hearing/auditory processing), 2 (aphasia/apraxia), 4 (vision impairment), 10 (spastic gait) and 13 (incontinence)
- Sustained change from Baseline in total NFS score

  "Sustained change" is defined as the same total score for NFS items 1, 2, 4, 10, and 13 and no change >1 in NFS total score observed in two the consecutive Visits 6–8, and Visits 11-12, respectively. If "sustained change" definition is not met, the average of the two scores of Visit 6 and 8, and Visit 11 and 12, respectively, will be used. If NFS total score differs by 1 point between V6-8, or V11-12, the higher of the two scores is used.
- Change from baseline in Loes MRI severity score
- Change from baseline in Gadolinium Intensity Score (GIS)
- Overall survival of patients who have not undergone HSCT
- Number of patients meeting HSCT criteria

2.2.3 Exploratory Efficacy Endpoints

The exploratory endpoints will be:

- Change from Baseline in T2/FLAIR lesion volume
- Change from Baseline in white matter fiber structure as determined by fractional anisotropy (FA) and/or apparent diffusion coefficient (ADC) on DTI
- Change from Baseline in cerebral blood flow and blood volume, capillary mean transient time heterogeneity (CHT) and constant of permeability (K2 and Kapp) as determined by DSC MRI
- Changes from Baseline in the levels of each biomarker in plasma and CSF (optional)

2.2.4 Week 24 Interim Evaluation Endpoints

The continuation criteria and the number of patients meeting "arrested disease" will be assessed at week 24.

The endpoints to inform on study continuation at Week 24 will be:

- Number of patients meeting (week 24) "arrested disease"
- Number of patients meeting lesion growth deceleration
- Number of patients meeting "continuation criteria"

2.2.5 Safety and Tolerability Endpoints

The safety and tolerability endpoints will be:

- Adverse events (AEs)
- Serious adverse events (SAEs) and suspected unexpected serious adverse reactions (SUSARs)

- Vital signs (body weight, height, blood pressure, pulse rate, and body temperature)
- Physical examination
- 12-lead ECG (heart rate and the following intervals: PR, RR, QRS, QT, QT corrected for heart rate using Fridericia's formula [QTcF]).
- Echocardiogram
- Clinical laboratory tests
- Palatability of study drug

3. INVESTIGATIONAL PLAN

3.1 Study Design

This is an open-label, phase 2, multicenter study in male pediatric patients, aged ≥2 and ≤12 years, with a diagnosis of the cALD phenotype of X-linked ALD. After written informed consent by the parent/legal guardian, or authorized legal representative and completed assent as appropriate, is obtained, and after completion of all screening evaluations with all inclusion criteria and exclusion criteria satisfied, patients will be enrolled to be treated with MIN-102. Study patients enrolled may present different MRI status prior to first dose of study drug: patients without Gd-enhancing cerebral lesions (referred to as 'Population 1' in the protocol) and patients presenting Gd-enhancing lesions (Population 2). It is anticipated that approximately 13 patients will be included in the study. Additional patients may be enrolled after agreement with the Sponsor to ensure that the study recruits 13 evaluable patients who receive MIN-102 treatment. Decisions regarding patient replacement will be documented. Patients who undergo HSCT or do not meet "arrested disease" criteria will not be replaced. However, patients who drop out by withdrawal of consent by parents for non-treatment related causes may be replaced. Patients dropping out will be encouraged to remain in the study and attend all further scheduled efficacy and safety assessments.

Each patient will undergo screening for a maximum of 15 days, followed by a treatment period. If the screening procedures are performed within 7 days prior to Baseline (V0), all results obtained from the screening evaluations will serve as the Baseline values and pre-dose Baseline procedures will only consist of the clinical efficacy assessment (NFS-MFD), the blood sampling for biomarkers and MIN-102 plasma levels, and the optional CSF sampling for biomarkers. Patients will be evaluated at the Screening Visit, at the Baseline Visit when the first dose of study drug is administered, and at regular biweekly intervals thereafter until the Week 12 (Visit 6). After Visit 6, evaluations will occur at Week 18 (Visit 7), Week 24 (Visit 8), Week 36 (Visit 9), Week 48 (Visit 10), Week 72 (Visit 11), Week 96 (Visit 12), and at further 24-week intervals thereafter until end-of-treatment criteria apply (Appendix B).

Hematopoietic stem cell transplantation may be performed at any time point after the Baseline Visit. The patient will adhere to the visit schedule until initiation of HSCT procedures. Prior to initiation of HSCT procedures, the patient will be scheduled for another visit irrespective of the time since the Baseline Visit: the "Visit prior HSCT". Last dose of study drug will be administered one day prior to this visit. This visit is the final visit in the study for patients undergoing HSCT.

Patients meeting any of the following criteria ("HSCT criteria") will be scheduled for HSCT:

- GIS of 1, 2 or 3, or
- Significant T2/FLAIR lesion growth from Baseline as assessed by central reading, present at two consecutive MRIs at least 12 weeks apart from each other.

If HSCT criteria are met at the first visit, but not at the second visit, another visit will be scheduled 12 weeks after the second visit to confirm presence or absence of criteria for HSCT. If patients meet HSCT criteria for the first time at Visit 10 (Week 48) or later, an unscheduled visit will occur 12 weeks (±15 days) afterwards. If HSCT criteria are not met at the unscheduled visit, the patient will continue with the regular visit schedule. If HSCT criteria are met at two consecutive visits, the patient will be scheduled for HSCT.

The investigator can terminate treatment, if his/her overall assessment of lesion progression mandates immediate scheduling of HSCT although "HSCT criteria" are not met. If treatment is stopped, all assessments equivalent to the "Visit Prior to HSCT" will be performed one day after last dose of study drug. The Follow-up Visit will be scheduled 4 weeks (±5 days) after last dose of study drug.

3.2 Treatment

All patients will receive MIN-102 (open label). No randomization or blinding will be used in this study.

3.2.1 Treatment Compliance

Compliance will be determined at the study site at each visit after the Baseline Visit, when patients return all previously used and unused bottles. The number of empty bottles will be determined, and the amount as volume in mL of suspension left in returned bottles will be assessed. Compliance with study drug will be calculated as the actual volume in milliliters taken divided by the scheduled volume in milliliters as prescribed by the investigator, expressed as a percentage.

Compliance will also be assessed by determination of MIN-102 levels in plasma samples.

3.2.2 Duration of Treatment

Patients will receive treatment until the first occurrence of any of the following end-of-treatment criteria:

1. Patient undergoes HSCT having met the following criteria:
   - GIS of 1, 2 or 3, or
   - Significant T2/FLAIR lesion growth assessed by central reading,

   These criteria must be present at two consecutive MRIs at least 12 weeks apart from each other.
2. Patient undergoes HSCT, if investigators' overall assessment of lesion progression mandates immediate scheduling of HSCT although "HSCT criteria" are not met.

   Patients who undergo HSCT according to criteria 1 and 2 above may receive study drug until the day before the last study visit prior to initiating HSCT procedures ("Visit Prior to HSCT"). This visit will occur a minimum of 5 days before first administration of

myeloablative medication.

3. Study fails to meet “continuation criteria” at week 24.
4. Patient fails to meet primary endpoint of “arrested disease” at Week 96.
5. The final evaluation of the study is performed at Week 96 and shows that less than 4 of 13 enrolled patients meet criteria for “arrested disease”.
6. The patient’s parent/legal guardian, or authorized legal representative withdraws consent.
7. In the clinical judgment of the investigator, the patient’s general health status declines to an extent that the risks of treatment outweigh the potential benefits.
8. MIN-102 is commercially available for the treatment of cALD.

Patients who undergo HSCT will receive study drug until the day before the last study visit prior to initiating HSCT procedures. This visit will occur a minimum of 5 days before first administration of myeloablative medication.

4. GENERAL CONSIDERATIONS FOR DATA ANALYSIS

All data collected including the point of discontinuation or withdrawal will be included in the summary unless the patient withdraws consent to the use of their data should be included in summary and analysis.

All data obtained in this study and documented through the electronic case report forms (eCRFs) will be entered into the study database. In general, all data collected prospectively, and any derived data will be presented in patient data listings, for all enrolled patients. Listings will be ordered by patient number, visit, and assessment or event date.

In general, continuous variables will be summarized by presenting the population sample size (N), number of patients with available data (n), mean, standard deviation (SD), median, minimum, and maximum. Categorical variables will be summarized by presenting the population size (N), number of patients with available data (n), number of patients in each category, and the percentage of patients in each category. Unless otherwise noted, the denominator to determine the percentage of patients in each category will be based on the number of patients with available data. Select ordinal data may be summarized using both descriptive statistics and counts and percentages of patients in each category, as appropriate.

For analyses where confidence intervals (CI) are provided, a significance level of 0.05 will be used and confidence intervals will be calculated using Clopper-Pearson exact CI (Clopper, C; Pearson, E.S., 1934).

The data analyses will be performed using SAS® System version 9.4 or higher (SAS, Cary, NC), except where other software may be deemed more appropriate.

CTI Clinical Trial and Consulting Services (Covington, KY) will perform all planned analysis described in the SAP on behalf of Minoryx Therapeutics S.L.

4.1 Analysis Quality Control Procedure

Once all the source verification is complete, all queries are resolved, and the database has been updated appropriately, the database will be locked and made available to CTI Biostatistics for final analysis.

Data may be pulled by CTI Biostatistics for interim analysis at a time when source verification and query resolution is ongoing.

All SAS programs used to create analysis datasets, tables, and listings are double programmed. The SAS outputs will be compared, and the programs will be updated until the outputs match

4.2 Analysis Sets

4.2.1 Safety Analysis Set (SAS)

All patients who took at least 1 dose (partial or complete) of MIN-102.

4.2.2 Modified Intent-To-Treat Analysis (mITT) Set

All patients who took at least 1 dose (partial or complete) of MIN-102 and had at least 1 post-baseline MRI assessment with gadolinium. The mITT will further be broken down into Population 1, which will include patients without Gd-enhancing cerebral lesions at baseline and Population 2, which will include patients presenting Gd-enhancing lesions at baseline.

4.2.3 Per-Protocol Analysis Set (PPS)

All patients in the mITT analysis set without a major protocol violation.

4.2.4 Pharmacokinetic Analysis Set (PKS)

All patients who took at least 1 dose (partial or complete) of MIN-102 and had at least 1 plasma sample with MIN-102 concentration. Protocol deviations which may affect the analysis (for example incomplete dose or sample out of the window), will be evaluated on a case-by-case basis and profiles or individual time points may be excluded from summaries and analysis.

4.2.5 Pharmacodynamics Analysis Set (PDS)

All patients who took at least 1 dose (partial or complete) of MIN-102 and had at least 1 post-baseline measurable biomarker concentration available in plasma or CSF. Protocol deviations which may affect the analysis (for example incomplete dose or sample out of the window), will be evaluated on a case-by-case basis and profiles or individual time points may be excluded from summaries and analysis. The PDS will further be broken down into Population 1, which will include patients without Gd-enhancing cerebral lesions at baseline and Population 2, which will include patients presenting Gd-enhancing lesions at baseline.

4.3 Eligibility for Efficacy Analysis

As set forth in the protocol, patients are allowed to leave the study to undergo HSCT even if the study's "HSCT criteria" are not met (Investigator decision). As a consequence of this study design, patients may leave the study before they have provided sufficient data for analysis of efficacy. Evaluability" of patients will be based on the following criteria:

- Patients will be considered "evaluable" if they have provided enough data to assess "arrested disease". To assess "arrested disease", ≥ 2 consecutive MRIs spanning a minimum of 12 weeks are needed.

- Patients will be considered “non-evaluable” if they have not provided enough data to assess “arrested disease”. To assess “arrested disease” ≥ 2 consecutive MRIs spanning a minimum of 12 weeks are needed.

Individual data will still be reported, but “non-evaluable” patients will not be included in analysis of “arrested disease” (See Section 6.1.1)

4.4 Handling of Dropouts or Missing Data

Missing values will not be substituted by estimated values but treated as missing in the statistical evaluation.

For the time to onset of HSCT, this will be defined as the day when the patient began myeloablative conditioning. The time unit will be “days” and will be calculated as:

Time to onset of HSCT (days) = (Date of onset of myeloablative conditioning) - (Date of first study drug administration) + 1

If a patient is shortlisted for HSCT but ultimately does not undergo HSCT, the date used for the time-to-event analysis will be right censored at the time they leave the study (complete the study, or withdrawal from the study), or their last recorded visit if lost to follow-up. If a patient is not able to attend the protocol specified “visit prior to HSCT” before undergoing HSCT, the last protocol visit will be mapped as ‘visit prior to HSCT”, when applicable. The final decision on visit remapping will be made at the data review meeting (see Section 4.5.2).

4.5 Multiple Comparisons

A hierarchical, serial gatekeeper testing strategy will be used to protect the overall type I error rate of α=0.05 (one-sided) when testing the co-primary endpoints at Visit 12 (Week 96) (as described in section 6.1.2).

4.6 Data Derivations and Transformations

4.6.1 Baseline and Change from Baseline

In general, the last recorded value on or prior to the date of Visit 0 will serve as the baseline measurement for all endpoints.

Change from baseline will be calculated as the post-baseline value minus the baseline value.

Percentage change from baseline will be calculated as the change from baseline divided by the baseline value, multiplied by 100.

4.6.2 Drug Compliance

Compliance = [(Volume dispensed – Volume Returned)]/[MIN-102 Dosing] * 100.
MIN-102 Dosing=(days elapsed * dose amount) for each dose.

4.6.3 Predicted Area Under Curve

The predicted AUC post-baseline is calculated below:

- Ages 2-5yrs, AUC = 26.73 * Cpredose + 58.24
- Ages 6-11yrs, AUC = 27.15 * Cpredose + 61.31 and

- Age 12 years, AUC = 26.71 * Cpredose + 57.6.

Cpredose = the trough value (Cmin) or concentration 24 hours after the last dose at the specified visit and age is the age at baseline.

4.6.4 Data Review Meeting

A data review meeting will be held before the assessments at Week 24 and Week 96 where patients will be classified as evaluable or not evaluable for the purposes of the analysis. Non-evaluable patients will still be included in the corresponding data listings but will not be part of the efficacy analysis.

Data from the specific visits may also be considered for non-inclusion in certain analyses (e.g., PK data where there has been a period of non-compliance with the study drug).

Additionally, data will be reviewed to determine whether any remapping of visits is applicable. Decisions made in this data review meeting will be documented.

5. DEMOGRAPHIC AND BASELINE CHARACTERISTICS

5.1 Disposition of Patients

A table of counts of all patients in each analysis set will be provided as well as the number of patients in Population 1 and Population 2. Reasons for not completing study as planned and reasons for premature withdrawal will be tabulated.

Eligibility criteria, screening failures, and informed consent date signed will be listed for all screened patients.

5.2 Protocol Deviations

All protocol deviations will be summarized in the clinical study report. After database lock, the study team will assess all protocol deviations on a patient-by-patient basis to ascertain how the deviation affects patient inclusion into the various analysis sets. Any deviation may affect the patient’s eligibility for 1 or more of the analysis sets. The impact of protocol deviations on assessment of the primary endpoint and the handling of missing/invalid data will be carefully investigated.

A listing of protocol deviations will be provided and will include type of protocol deviation, the action taken, date it occurred and whether it is a major or minor deviation for SAS.

5.3 Demographics

Descriptive statistics will be used to summarize the demographic characteristics. Demographic data will include age, race, and ethnicity. The demographic data will be listed by patients for SAS.

5.4 Medical History

All medical history will be classified by System Organ Class (SOC) and Preferred Term (PT) using Medical Dictionary for Regulatory Activities (MedDRA version 23.0). The number and percentage of patients will be summarized for each SOC and PT.

A listing of all medical history will be displayed and sorted by patient ID, start and stop dates

for SAS.

5.5 Prior and Concomitant Medications

Concomitant medications will be coded using World Health Organization (WHO) drug classifications version B3 Global 2020Q1. The number and percentage of patients using concomitant medications will be tabulated by default using the Anatomical Therapeutic Chemical (ATC) classification system and by preferred name for the SAS.

All concomitant medication data will be listed, sorted by patient ID, start and stop date for SAS.

6. EFFICACY ANALYSIS

Comparisons and/or descriptive summaries will be provided for all efficacy and safety parameters, as available at the given time points:

- Visit 8 (24 weeks after start of treatment) or Visit Prior to HSCT if earlier, and
- Visit 12 (96 weeks after start of treatment) or Visit Prior to HSCT if earlier

A comparison of MRI parameters and also of clinical endpoints will be performed in those instances where there is available natural history data of sufficient quality to make such comparison. The primary comparison to the natural history will be performed with the most up-to-date and comparable data available. Natural history data generated by Mallack et al. (2021) or later publications will be the basis of the primary comparison as this data was generated with the same MRI protocol and highly comparable assessment through expert manual segmentation of brain lesions. Further comparisons of the MRI parameters will be performed with a separate sample of patients derived from the database at the University of Minnesota (refer to Protocol Section 3.2 for information); however, it is expected that this comparison will be less reliable as this data was obtained using automatic segmentation software aimed to assess Multiple Sclerosis brain lesions.

All efficacy analysis will be performed using the mITT analysis set.

6.1 Key Efficacy Definitions

6.1.1 Arrested Disease Criteria

6.1.1.1 Arrested Disease Criteria at Visit 8 (Week 24) or Visit prior to HSCT

"Arrested disease" is defined using the following parameters at week 24:

- Change in Neurological Function Score (NFS) from Baseline ≤1 point
- Free of MFD
- Lack of lesion progression on MRI, fulfilling any of the following:
    - No conversion to Gd+ lesions (Population 1) defined as GIS score of 0
    - Disappearance of persistent Gd+ lesions (Population 2) defined as change in GIS score from 1, 2 or 3 to 0. Persistent Gd+ lesions is defined as those present

in ≥ 2 consecutive MRIs spanning a minimum of 6 months.

- No significant growth of T2/FLAIR lesions compared to the previous MRI (both populations) as assessed by central reading

6.1.1.2 Arrested Disease Criteria at Visit 12 (Week 96) or Visit prior to HSCT

"Arrested disease" is defined using the following parameters at week 96:

- Change in Neurological Function Score (NFS) from Baseline ≤5 points
- Free of MFD
- Lack of lesion progression on MRI, fulfilling any of the following:
    - No conversion to Gd+ lesions (Population 1) defined as GIS score of 0
    - Disappearance of persistent Gd+ lesions (Population 2) defined as change in GIS score from 1, 2 or 3 to 0. Persistent Gd+ lesions is defined as those present in ≥ 2 consecutive MRIs spanning a minimum of 6 months.
    - No significant growth of T2/FLAIR lesions compared to the previous MRI (both populations) as assessed by central reading

6.2 Primary Efficacy Endpoint Analysis

The requirement for a successful study based on the recruitment of 13 patients is set down in the protocol as follows:

- The study will be considered successful if a minimum of 4 patients meet all "arrested disease" criteria at Week 96, except for MFD where >70% of all evaluable patients at Week 96 have to be free of MFD.

To formulate these requirements in a structured way involves having co-primary (binary) endpoints at Week 96 defined as follows:

- Co-Primary Endpoint 1: Arrested disease at Week 96 not including being free of MFD.
- Co-Primary Endpoint 2: Arrested disease at Week 96 including being free of MFD

The null hypothesis for co-primary endpoint 1 is H_{01}: $\theta_1 \leq 0.1$ where θ_1 is the true proportion satisfying all "arrested disease" criteria, not including being free of MFD, in the population

The null hypothesis for co-primary endpoint 2 is H_{02}: $\theta_2 \leq 0.24$ where θ_2 is the true proportion satisfying all "arrested disease" criteria including being free of MFD among those satisfying all "arrested disease" criteria not including being free of MFD.

The endpoints will be tested in a hierarchical manner with endpoint 2 being evaluated for confirmatory conclusions only if the null hypothesis for endpoint 1 is rejected at the one-sided 5% level; otherwise, the p-value for this hypothesis will be considered nominal. All the individual components of the primary endpoint will be descriptively summarized by Population 1, Population 2, and overall.

6.3 Secondary Efficacy Endpoints and Analyses

The secondary efficacy endpoints will be:

- Sustained change from Baseline in the score composed of NFS items 1 (hearing/auditory processing), 2 (aphasia/apraxia), 4 (vision impairment), 10 (spastic

gait) and 13 (incontinence)

- Sustained change from Baseline in total NFS score "Sustained change" is defined as the same total score for NFS items 1, 2, 4, 10, and 13 and no change >1 in NFS total score observed in two the consecutive Visits 6–8, and Visits 11-12, respectively. If "sustained change" definition is not met, the average of the two scores of Visit 6 and 8, and Visit 11 and 12, respectively, will be used. If NFS total score differs by 1 point between V6-8, or V11-12, the higher of the two scores is used.

  Change from Baseline for observed scores of NFS items 1, 2, 4, 10, and 13, and for NFS total score will also be descriptively summarized for each visit without applying the "sustained change" rules.
- Change from baseline in Loes MRI severity score
- Change from baseline in Gadolinium Intensity Score (GIS)
- Overall survival of patients who have not undergone HSCT
- Number of patients meeting HSCT criteria

Each of the endpoints will be descriptively summarized, including confidence intervals, and graphical representations will be provided (mean values or proportions over time, or Kaplan-Meier curves in the case of time-to-event endpoints. All secondary endpoints will be descriptively summarized by Population 1, Population 2, and Overall.

Time to first Gadolinium enhancement (change from GIS = 0 to GIS = 1, 2 or 3; Population 1 only) and time to onset of HSCT will be assessed using Kaplan-Meier (KM) method.

6.4 Exploratory Efficacy Endpoints and Analyses

The exploratory endpoints will be:

- Change from Baseline in T2/FLAIR lesion volume
- Change from Baseline in white matter fiber structure as determined by fractional anisotropy (FA) and/or apparent diffusion coefficient (ADC) on DTI
- Change from Baseline in cerebral blood flow and blood volume, capillary mean transient time heterogeneity (CHT) and constant of permeability (Kapp) as determined by DSC MRI
- Changes from Baseline in the levels of each biomarker in plasma and CSF (optional)

Additional calculations will be derived for normalized lesion volume, fold change from baseline, fold change from baseline per month, growth rate [velocity (cc/month)], and acceleration (cc/month^2). Each of this will be descriptively summarized and graphical representations to compare with Natural History data from literature.

Growth rate (velocity) is defined as the increase in T2/FLAIR lesion volume since previous MRI divided by the number of months elapsed since that previous MRI. Assessment of growth rate will be based on the data obtained at the specified visits that are at least 12 weeks (±15 days) apart from each other: baseline visit, Visit 6 (Week 12), Visit 8 (Week 24), Visit 9 (Week 36), Visit 10 (Week 48), Visit 11 (Week 72), Visit 12 (Week 96), and further visits, as applicable. If after Visit 10, an MRI is performed at an unscheduled visit or visit prior to HSCT. The growth rate will only be evaluated at this intermediate timepoint if at least 12 weeks (±15

days) have elapsed from the previous visit.

Acceleration is defined as the change in growth rate (velocity) divided by the number of months elapsed. Assessment of acceleration will be performed if at least 2 valid assessments of velocity have been obtained.

All the exploratory endpoints will be descriptively summarized by Population 1, Population 2, and Overall. Each of the endpoints will be descriptively summarized, including confidence intervals, and graphical representations.

6.5 Week 24 Interim Evaluation

The continuation criteria and the number of patients meeting “arrested disease” will be assessed at week 24.

Arrested disease at week 24 is defined in section 6.1.1.1. No decisions in terms of futility and efficacy will be made at this timepoint based on evaluation of “arrested disease". “arrested disease” at week 24 will be descriptively summarized, including confidence intervals, and graphical representations and by Population 1, Population 2, and Overall.

The study will be continued when the one-sided 95% confidence interval for the proportion of evaluable patients meeting the week 24 definition of arrested disease or lesion growth deceleration at Week 24 as per overall assessment from central reading is above 10%.

To evaluate the continuation condition, the following endpoints will be presented:

- Number of patients meeting “arrested disease” at Week 24
- Number of patients meeting lesion growth deceleration at Week 24
- Number of patients meeting “continuation criteria” at Week 24

Additionally, the secondary and exploratory endpoints will be reported in a descriptive manner and graphical representations will be provided where applicable.

Safety, pharmacokinetic and pharmacodynamic parameters will also be reported

6.6 Sensitivity analyses

The following sensitivity analysis of the primary endpoint will be performed:

- Number of patients meeting “arrested disease” criteria at Visit 12 (Week 96) or visit prior to HSCT including all patients (i.e. including patients determined as “non-evaluable” in the data review meeting). For this sensitivity analysis “non-evaluable” patients will be considered as not meeting “arrested disease”.

7. PHARMACOKINETIC EVALUATION

Pharmacokinetic data will be summarized using appropriate descriptive statistics and listed and summarized in tabular and/or graphical form.

The PK endpoints include the following parameters: Area Under Curve (AUC), plasma concentration at 2 hours (C2h), and minimum plasma concentration (Cmin), Additional PK analyses may be performed if deemed appropriate.

8. BIOMARKER EVALUATION

Biomarker data (absolute values and change from baseline) will be summarized using descriptive statistics and listed and summarized in tabular and/or graphical form by Population 1, Population 2, and Overall. The PDS will be used for all the biomarkers in plasma and CSF listings and summarization of parameters. Additional biomarker analyses may be performed if deemed appropriate.

Biomarkers analyzed in plasma and CSF will include: neurofilament light chain (NfL), adiponectin, matrix metalloproteinase (MMP)-9, interleukin (IL)-18, interleukin (IL)-1β, interleukin-1 receptor antagonist (IL-1ra), monocyte chemoattractant protein-1 (MCP-1) and chitotriosidase. Further parameters may be added with emerging science.

Biomarker concentration will be considered as PD endpoints.

Biomarkers concentrations below the limit of quantification (BLQ) in pre-dose samples and in samples taken before the time of the first quantifiable value will be set to half of the corresponding limit of quantification.

The biomarker concentrations BLQ after quantifiable concentration will be set to half of the limit of quantification.

9. SAFETY AND TOLERABILITY ANALYSIS

Safety assessments will focus on the type, severity, and frequency of individual AEs and laboratory tests, vital signs, and ECG abnormalities. The analyses will generally be descriptive in nature and will be based on the SAS.

Descriptive statistics for palatability will be summarized over time and will be based in the SAS.

9.1 Exposure

The target dose and exposure in humans are based on the PK findings of the phase 1 study MT-1-01 in healthy adult males and on PBPK modeling and supported by calculations of MIN-102 exposure with respect to pharmacodynamic effects in preclinical pharmacology.

MIN-102 exposure including total number of doses received, the expected total dose, the actual dose administered, and duration of exposure will be summarized and presented.

9.2 Adverse Events

An AE is any untoward medical occurrence associated with the use of a drug in humans, whether or not considered drug related. An adverse event (also referred to as an adverse experience) can be any unfavorable and unintended sign (e.g. an abnormal laboratory finding), symptom, or disease temporally associated with the use of a drug, without any judgment about causality. Please refer to Protocol Section 6.1 for detailed definitions.

9.2.1 Serious Adverse Events

An adverse event is considered "serious" if, in the view of either the investigator or sponsor, it results in any of the following outcomes:

- Death

- A life-threatening adverse event. This refers to an event that, in the view of either the investigator or sponsor, places the patient at immediate risk of death. It does not include an AE that, if it had occurred in a more severe form, might have caused death.
- Inpatient hospitalization or prolongation of existing hospitalization. This refers to hospital admission required for treatment of the AE. This does not include “social or convenience” hospitalization for nonmedical causes such as lack of transportation to home; admissions of less than 24 hours for purposes of observation; confinement in, for example, a respite unit, a skilled nursing unit, or rehabilitation facility; or confinement due to a planned or an unplanned reason unrelated to the study. Emergency room visits that do not result in admission to the hospital should be evaluated for one of the other serious outcomes (e.g. life-threatening; required intervention to prevent permanent impairment or damage; other serious medically important event).
- Persistent or significant disability/incapacity or substantial disruption of the ability to conduct normal life functions
- Congenital anomaly/birth defect

Important medical events that may not result in death, be life threatening, or require hospitalization may be considered serious when, based upon appropriate medical judgment, they may jeopardize the patient and may require medical or surgical intervention to prevent one of the outcomes listed in this definition. Examples of such medical events include allergic bronchospasm requiring intensive treatment in an emergency room or at home, blood dyscrasias or convulsions that do not result in inpatient hospitalization, and the development of drug dependency or drug abuse.

Note that all SAEs are also AEs.

9.2.2 Severity of Adverse Events

The severity of AEs will be graded using the most current version of the Common Terminology Criteria for Adverse Events 5-point scale:

- Mild (grade 1): asymptomatic or mild symptoms: clinical or diagnostic observations only; intervention not indicated
- Moderate (grade 2): minimal, local or noninvasive intervention indicated; limited age-appropriate instrumental activities of daily living
- Severe (grade 3): severe or medically significant but not immediately life threatening: hospitalization or prolongation of hospitalization indicated; disabling; limiting self-care activities of daily living
- Life threatening (grade 4): life-threatening consequences; urgent intervention indicated
- Death (grade 5): death related to AE

9.2.3 Relationship of Adverse Events to Study Drug

The relationship of any AE to the study drug will be assessed and graded as related or not related. Adverse events will be considered “related” when there is a reasonable possibility that

the drug caused the event. Adverse events will be considered "unrelated" when it appears very unlikely that the drug caused the event, such as when an alternate cause of the event is evident.

9.2.4 Adverse Event Summaries

Adverse event data will be displayed in listings by patient. The number and percentage of patients with AEs will be tabulated by system organ class (SOC) and preferred term (PT). A patient with multiple AEs within a SOC or PT will be counted once toward the total for the total for the SOC or PT.

All AEs (serious and non serious) occurring after completion of the informed consent process and before the end of study, regardless of relationship to study drug, will be included and classified by system organ class (SOC) and preferred term (PT) using the Medical Dictionary for Regulatory Activities (MedDRA version 23.0).

For AEs, the following will be summarized and presented for the SAS analysis set:

i. An overall summary of AEs, which includes:
   a. the number and percentage of patients experiencing an AE
   b. the number and percentage of patients experiencing an AE by strongest relationship to study medication
   c. the number and percentage of patients experiencing an AE by greatest severity
   d. the number and percentage of patients experiencing an SAE
   e. the number and percentage of seriousness criteria for SAE

ii. the number and percentage of patients experiencing an AE by SOC and PT

iii. the number and percentage of patients experiencing an AE by SOC, PT and the greatest severity

iv. the number and percentage of patients experiencing an AE by SOC, PT and the strongest relationship to study medication

v. the number and percentage of patients experiencing an SAE by SOC and PT

In the overall summary of AEs table (i), besides tabulating the number and percentage of patients, the total number of AE episodes will also be provided. If a patient has repeated episodes of a particular AE, all episodes will be counted in the summary table.

A patient with more than one type of AE in a particular SOC will be counted only once in the total number of patients experiencing AEs in that particular SOC. Since a patient could have more than one type of AE within a particular SOC, the sum of patients experiencing different AEs within the SOC could appear larger than the total number of patients experiencing AEs in that SOC. Similarly, a patient who has experienced an AE in more than one SOC will be counted only once in the total number of patients experiencing AEs in all SOCs.

All occurrences of all AEs will be listed for each patient. The listing will contain the following information: verbatim term, SOC, PT, severity, relationship to study medication, date and day of onset, date and day of resolution or ongoing, treatment given to treat the adverse event, if any, the outcome, whether the event was an SAE, seriousness criteria for SAE, whether it led to withdrawal. Listings will be sorted by patient identification number, onset date, SOC, and

PT. If the onset date is completely missing, then these events will be presented first. If the onset date is missing a month or a day, then these events will be presented before any complete dates.

9.3 Cytological Clinical Laboratory Assessments

Blood and urine samples for clinical laboratory assessments will be collected at study visits as indicated in Appendix B and C. Blood samples will be taken in fasting state (if possible). The following parameters will be analyzed:

– Blood chemistry: cortisol, adrenocorticotropic hormone (ACTH), aldosterone (only at V-1), and HbA1c (only at V-1, Visit Prior to HSCT or FUV); total bilirubin, direct bilirubin, alkaline phosphatase (ALK), aspartate aminotransferase (AST), alanine aminotransferase (ALT) (at all visits except Visit 0); gamma glutamyl transferase, lactate dehydrogenase, creatinine, creatinine clearance, blood urea nitrogen, uric acid, cholesterol, triglycerides, total protein, albumin, glucose, glucose (random), inorganic phosphate, sodium, potassium, calcium, and chloride (at all visits except Visit 0, 1, 3, 4, 5 and 7); NT-proB-type Natriuretic Peptide (BNP) (at all visits except Visit -1, 1, 3, 4, 5 and 7).

– Prothrombin time (at all visits except Visit 0), INR; for patients of less than 13.75 kg (30.31 lbs) of body weight, prothrombin time will only be analyzed if liver parameters show alterations suspicious of DILI (see section 6.2 of protocol).

– Hematology: leukocytes, erythrocytes, hemoglobin, hematocrit, thrombocytes, lymphocytes (absolute and differential), monocytes (absolute and differential, eosinophils (absolute and differential, basophils (absolute and differential, neutrophils (absolute and differential, mean corpuscular volume, mean corpuscular hemoglobin, and mean corpuscular hemoglobin concentration (at all visits except Visit 0, 1, 3, 4, 5 and 7).

– Blood spot glucose check (at all visits starting at V0)

– Urinalysis [qualitative]: hemoglobin, urobilinogen, ketones, glucose, protein, bilirubin, leukocytes, pH, and nitrite (at all visits except Visit 0, 1, 3, 4, 5 and 7).

– Cytological examination will assess the presence of abnormalities in bladder epithelial cells (at all visits except Visit 0, 1, 3, 4, 5 and 7).

Clinical laboratory data will be summarized by presenting descriptive statistics of raw data and change from baseline values at each visit.

A listing of all clinical laboratory data will be presented and will include clinical significance and abnormal lab values for SAS.

9.4 Vital Signs

Systolic and diastolic blood pressure, pulse rate, body temperature and weight will be measured in each visit. Height will be measured at screening only.

Patients can be contacted by phone between scheduled visits and be advised to monitor their weight frequently and report back to the investigator in case of weight gain. If required, an Unscheduled visit may be scheduled at any time.

Vital sign data will be summarized by presenting descriptive statistics of raw values and change from baseline values at each visit.

A listing of all vital signs results will be presented for SAS.

9.5 Electrocardiogram

A standard 12-lead ECG will be recorded after the patient has been resting in the supine position for at least 5 minutes. The following ECG parameters will be recorded using an ECG machine equipped with computer-based interval measurement heart rate, PR-interval, RR-interval, QRS-duration, QT-interval, and QTcF-interval (Fridericia's [3]).

The investigator will provide interpretation of the ECG profile. The ECG must be recorded in triplicate with 3 serial readings performed 5 minutes apart approximately 2 hours (±0.5 hours) after the daily dose has been administered.

ECG data will be summarized by presenting descriptive statistics of raw data and change from baseline values at each visit for SAS.

9.6 Echocardiogram

An echocardiogram will be performed only at Screening. It will be recorded locally and assessed by the local cardiologist or qualified delegate.

Patients developing peripheral edema or BNP elevations >1.5 times the baseline value will require the echocardiogram repeated at each regular on-site visit (V1 – VX), until resolution of the peripheral edema or elevated BNP. Changes in echocardiogram from baseline value for all patients developing peripheral edema will be evaluated.

A listing of echocardiogram results including any abnormalities and the corresponding clinical significance will be presented for SAS.

9.7 Physical Examination

Physical examination will be performed at the designated on-site visits. Physical examination includes evaluation of the heart, lungs, abdomen, extremities, and skin, with specific attention to the presence of peripheral edema and signs or symptoms of heart failure. The physical examination will be conducted by the investigator or a medically qualified delegate with MD degree. At V1, V3, V4, V5, and V7 the patient will be only examined for the presence of peripheral edema. If this is a home visit, the examination may be performed by the home nurse.

Physical examination abnormalities will be listed and the number of patients with abnormalities will be summarized at each visit for each body system for SAS.

A separate listing will be provided for edema assessment for SAS.

9.8 Palatability Assessment

The palatability of study drug will be evaluated by the patient himself (depending on the age and cognitive ability), or by the parents' rating of ease of administration and patient's facial expression and/or verbal expression on a five-point hedonic from "super-good" to "super-bad" (Guinard, 2000[2]).

Count and frequencies will be used to summarize palatability at each visit.

A listing of palatability assessment will also be provided for the SAS.

10. SAMPLE SIZE

Sample size has been calculated to estimate the proportion of patients meeting “arrested disease” criteria to distinguish a rate of 40% with treatment vs. a rate of spontaneous 10% “arrested disease” in untreated patients. With 80% power, the study would enroll 13 participants and use a criterion of at least 4 participants achieving “arrested disease” for meeting the primary study objective with a one-sided significance level of 0.05.

Additional patients may be enrolled after agreement between the investigator and the Sponsor to ensure that the study recruits 13 evaluable patients who receive MIN-102 treatment. Patients who drop out as treatment failures defined as patients meeting “HSCT criteria”, shortlisted for HSCT for overall lesion progression in the judgment of the investigator, or not meeting “short-term arrested disease” criteria will not be replaced. However, patients who drop out by withdrawal of consent by parents or termination of treatment by the investigator for non-treatment related causes may be replaced. Decisions regarding patient replacement will be documented. Patients stopping treatment will be encouraged to remain in the study and attend all further scheduled efficacy and safety assessments.

11. REFERENCES

1. Clopper, C.; Pearson, E. S. (1934). "The use of confidence or fiducial limits illustrated in the case of the binomial". Biometrika. 26 (4): 404–413.
2. Engelen, M., S. Kemp, M. de Visser, B. M. van Geel, R. J. Wanders, P. Aubourg and B. T. Poll-The (2012). "X-linked adrenoleukodystrophy (X-ALD): clinical presentation and guidelines for diagnosis, follow-up and management." Orphanet J Rare Dis 7: 51.
3. Fridericia LS. Dir Systolendaeur in Elektrokardiogram bei normalen Menchen und bei Herzkranken. Acta Med Scand 1920;53:469-486.
4. Guinard, J.-X. (2000). "Sensory and consumer testing with children." Trends in Food Science & Technology 11(8): 273-283.
5. Eichler F, Duncan C, Musolino PL, Orchard PJ, De Oliveira S, Thrasher AJ, Armant M, Dansereau C, Lund TC, Miller WP, Raymond GV, Sankar R, Shah AJ, Sevin C, Gaspar HB, Gissen P, Amartino H, Bratkovic D, Smith NJC, Paker AM, Shamir E, O'Meara T, Davidson D, Aubourg P, Williams DA. Hematopoietic Stem-Cell Gene Therapy for Cerebral Adrenoleukodystrophy. N Engl J Med. 2017 Oct 26;377(17):1630-1638
6. Raymond, G.V., Aubourg, P., Paker, A., Escolar, M., Fischer, A., Blanche, S., Baruchel, A., Dalle, J.-H., Michel, G., Prasad, V., et al. (2019). Survival and Functional Outcomes in Boys with Cerebral Adrenoleukodystrophy with and without Hematopoietic Stem Cell Transplantation. Biol. Blood Marrow Transplant. 25, 538–548
7. Mallack, E.J., Turk, B., Yan, H., and Eichler, F.S. (2019). The Landscape of Hematopoietic Stem Cell Transplant and Gene Therapy for X-Linked Adrenoleukodystrophy. Curr Treat Options Neurol *21*, 61
8. Mallack, E.J., van de Stadt, S., Caruso, P.A., Musolino, P.L., Sadjadi, R., Engelen, M., and Eichler, F.S. (2020). Clinical and radiographic course of arrested cerebral adrenoleukodystrophy. Neurology *94*, e2499–e2507

9. Mallack, E.J., Askin, G., van de Stadt, S., Caruso, P.A., Musolino, P.L., Engelen, M., Niogi, S.N., and Eichler, F.S. (2021). A Longitudinal Analysis of Early Lesion Growth in Presymptomatic Patients with Cerebral Adrenoleukodystrophy. AJNR Am J Neuroradiol ajnr;ajnr.A7250v1

12. APPENDICES

12.1 Appendix A: Study Design

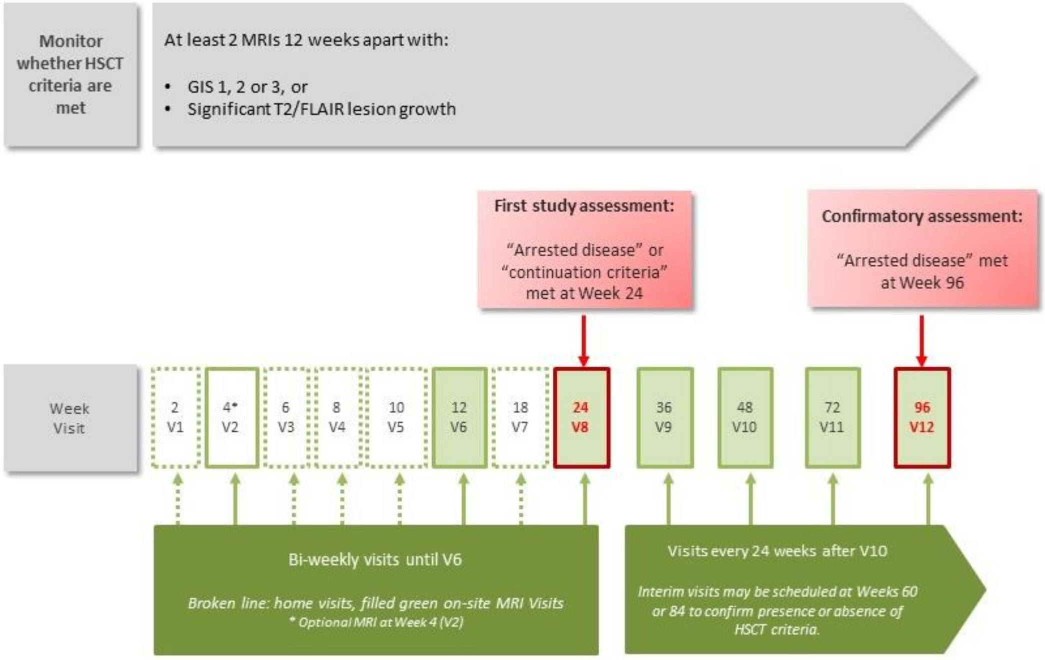

12.2 Appendix B: Flow Chart of Study Procedures

Table 1. Flow Chart of Study Procedures

**Visit Number**	**-1**	**0** Error! Reference source not found.	**1 ***	**2**	**3 ***	**4 ***	**5 ***	**6**	**7***	**8** Error! Reference source not found.	**9-12+**		
**Visit Name**	**Screening**	**Baseline**	**Visit 1**	**Visit 2**	**Visit 3**	**Visit 4**	**Visit 5**	**Visit 6**	**Visit 7**	**Visit 8**	**Visit 9, 10, 11, 12[3]…**	**Visit Prior to HSCT** Error! Reference source not found.	**FUV** Error! Reference source not found.
**Week**	**-2**	**0**	**2 (±5 days)**	**4 (±5 days)**	**6 (±5 days)**	**8 (±5 days)**	**10 (±5 days)**	**12 (±5 days)**	**18 (±15 days)**	**24 (±15 days)**	**36, 48, 72, 96, ..[3] (±15 days)**	**To be scheduled before starting HSCT procedures**	**4 weeks after last dose (±5 days)**
Informed consent	X												
Inclusion & exclusion criteria	X	X Error! Reference source not found.,Error! Reference source not found.											
Medical history/concomitant disease	X	X Error! Reference source not found., Error! Reference source not found.											
Demographics	X												
Prior medication	X	X Error! Reference source not found., Error! Reference source not found.											

Vital signs (weight, height, BP, pulse rate, & temperature)Error! Reference source not found.	X		X	X	X	X	X	X	X	X	X	X	X
Physical examination[20]	X		X	X	X	X	X	X	X	X	X	X	X
12-lead ECG (recorded in triplicate)Error! Reference source not found.	X	X		X		X		X		X	X	X	X
Echocardiogram Error! Reference source not found.	X												
NFS-MFD		X						X		X	X	X	X
Hematology, blood chemistry, PT Error! Reference source not found.	X	X	X	X	X	X	X	X	X	X	X	X	X
Urinalysis, urine cytology	X			X				X		X	X	X	X
Blood sampling for MIN-102 levels Error! Reference source not found.		X		X				X		X	X	X	
Blood sampling for biomarkers Error! Reference source not found.		X		X				X		X	X	X	
CSF sample for biomarkers Error! Reference source not found.		X						X			X[21]	X	
Blood spot glucose checkError! Reference source not found.		X	X	X	X	X	X	X	X	X	X	X	
Cerebral MRI	XError! Reference source not found.			XError! Reference source not found.				X		XError! Reference source not found.	X	X[16]	

Study drug dispensation[17]		X						X		X	X		
Study drug accountability				X				X		X	X	X	
Patient diary review[Error! Reference source not found.]			X	X	X	X	X	X	X	X	X	X	X
Palatability assessment[Error! Reference source not found.]		X		X				X		X	X		
Adverse event recording		X	X	X	X	X	X	X	X	X	X	X	X
Concomitant medication		X	X	X	X	X	X	X	X	X	X	X	X

*** Option for home visit.**

NT-proBNP = NT-proB-type Natriuretic Peptide; BP = blood pressure; CSF = cerebrospinal fluid; ECG electrocardiogram; FUV = Follow-up Visit; HbA1c = glycated hemoglobin; HSCT = hematopoietic stem cell transplantation; MRI = magnetic resonance imaging; NFS-MFD = Neurological Function Score- Major Functional Disabilities; PT = prothrombin time; V = Visit; ULN = upper limit of normal

1 No separate baseline procedures are performed for those procedures that were already performed at the Screening Visit (V -1). All results obtained at the Screening Visit (V -1) will serve as the baseline values. Except for post-dose blood sampling for MIN-102 levels- post-dose ECG and palatability assessment, all assessments at the Baseline Visit (V0) are to be performed pre-dose.
2 If the "Visit Prior to HSCT" is scheduled within 4 weeks to another study visit, the Investigator may decide to omit the other study visit and perform only the "Visit Prior to HSCT" in its place. This is the end of study visit for patients undergoing HSCT and will be scheduled up to 5 days prior to initiating HSCT procedures.
3 Unscheduled visits may occur at Weeks 60 and 84 to confirm presence or absence of HSCT criteria. Patients will continue visit schedule until any of the end-of-treatment criteria apply.
4 The Follow-up Visit will be scheduled 28 (±5) days after last dose of MIN-102 for patients who discontinue MIN-102 treatment after a decision not to undergo HSCT. If a patient drops out of the study before HSCT, the Follow-up Visit will be performed 28 (± 5) days after the last administration of study drug, or immediately before initiation of HSCT procedures, if these occur earlier than 28 days after last dose.
5 Confirmation and/or update of the screening information.
6 Temperature will be measured at each visit until HSCT procedures are initiated
7 At the Baseline Visit, ECGs will be recorded pre-dose and 2 hours (±0.5 hours) after administration of MIN-102. At all other visits, ECGs will be recorded 2 hours (±0.5 hours) after administration of MIN-102.
8 Echocardiogram will be repeated only in patients developing peripheral edema, or elevated NT-proBNP >1.5 times the Baseline value and out of normal range; in such instances, the echocardiogram will be repeated at each visit, until resolution of the peripheral edema or elevated NT-proBNP.
9 At the Screening Visit (V-1), the full panel of laboratory parameters will be assessed except for NT-proBNP and prothrombin time, which will be assessed at the Baseline Visit (V0). At Visits 1, 3, 4, 5 and 7, only total bilirubin, alkaline phosphatase, aspartate aminotransferase, alanine aminotransferase, and prothrombin time will be assessed. At all other visits, the full panel of laboratory parameters will be assessed. Adrenal function chemistries (ACTH, cortisol, aldosterone) will only be determined at V-1, and HbA1c will be determined only at V-1 and "Visit Prior to

HSCT" or FUV. For patients whose body weight is lower than 13.75Kg (30.31 Lbs.), prothrombin time will only be analyzed if liver parameters show alterations suspicious of drug induced liver injury (DILI).

10 At the Baseline Visit (V0) blood samples will be drawn pre-dose, and 2, 6, 12, and 24 hours post-dose requiring an overnight stay. At Visits V2 and V6, respectively, blood samples will be drawn pre-dose, and 2 hours post-dose. At further visits, blood samples for MIN-102 levels will be drawn immediately before MIN-102 administration. At the Visit Prior to HSCT, only one sample will be drawn.

11 Blood samples for biomarker analysis will always be taken pre-dose.

12 Cerebrospinal fluid sampling for biomarkers is optional. The sample at the Baseline Visit (V0) should be taken any time before the first dose. CSF sampling should be performed while the patient in sedated during the MRI, if possible.

13 Blood spot glucose test to be performed at 2 hours (±0.5 hours) after administration of daily dose.

14 The MRI parameters obtained at the Screening Visit (V-1) will be considered the baseline values for the evaluations. If the MRI cannot be scheduled during the Screening Visit, it will be performed at the Baseline visit (V0) prior to first dose of study medication. If a valid MRI obtained with the same study-specific specifications and within 15 days before Screening is available, this will be considered the Screening MRI and no extra MRI will be performed.

15 MRI is optional.

16 To minimize patients' exposure to sedation for the MRI and/or exposure to gadolinium, the Investigator may omit post-baseline scheduled MRIs at a given visit if they are not deemed necessary to monitor progression of cerebral lesions, except for V8 and/or the "Visit prior to HSCT".

17 For patients who are eligible to HSCT, but HSCT has not been performed, study drug is dispensed every 12 weeks; for patients with a final decision not to perform HSCT and continuing treatment, study drug is dispensed every 24 weeks.

18 A patient diary will be used in the study to keep track of IMP compliance, recording adverse events and any changes in concomitant medications

19 To be performed by child or parent depending on the age and cognitive ability.

20 At V1, V3, V4, V5, and V7 the patient will be only examined for the presence of peripheral edema

21 Only at Visit 12.

DocuSign

Certificate Of Completion

Envelope Id: 8B91F9EA186348869A6AB00B76D0F348 — Status: Completed

Subject: Complete with DocuSign: 2022-1005 MT-2-02 SAP v3 - 19Oct22_revMin_GP2_CTI_MP_RK_GP - Final .docx

Source Envelope:

Document Pages: 38	Signatures: 3	Envelope Originator:
Certificate Pages: 5	Initials: 0	Seth Bennett
AutoNav: Enabled		100 E. RiverCenter Blvd
EnvelopeId Stamping: Disabled		Suite 1600
Time Zone: (UTC-05:00) Eastern Time (US & Canada)		Covington, KY 41011
		sbennett@ctifacts.com
		IP Address: 208.102.95.169

Record Tracking

Status: Original<br>10/21/2022 5:54:52 AM	Holder: Seth Bennett<br>sbennett@ctifacts.com	Location: DocuSign

Signer Events	Signature	Timestamp
David McCollum<br>dmccollum@ctifacts.com<br>Director, Global Biostatistics<br>CTI Clinical Trial and Consulting Services<br>Security Level: Email, Account Authentication (Required)	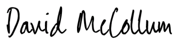<br>Signature Adoption: Pre-selected Style<br>Signature ID:<br>D431E5ED-B26F-4E75-A11A-D083AEFDB1F6<br>Using IP Address: 74.83.28.66<br><br>With Signing Authentication via DocuSign password<br>With Signing Reasons (on each tab):<br>I have reviewed this document	Sent: 10/21/2022 5:58:56 AM<br>Viewed: 10/21/2022 8:11:54 AM<br>Signed: 10/21/2022 8:12:43 AM

Electronic Record and Signature Disclosure:
Accepted: 7/14/2020 11:54:42 PM
ID: 2edc4721-867e-4245-8dad-1715494d30e0

Marc Martinell<br>mmartinell@minoryx.com<br>CEO / director<br>Security Level: Email, Account Authentication (Required)	DocuSigned by:<br>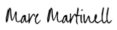<br>Signer Name: Marc Martinell<br>Signing Reason: I approve this document<br>Signing Time: 21-Oct-2022 | 3:07:56 AM PDT<br>239648531BDF4A4F9D8E53BE94F0FF8B<br><br>Signature Adoption: Pre-selected Style<br>Signature ID:<br>23964853-1BDF-4A4F-9D8E-53BE94F0FF8B<br>Using IP Address: 31.4.206.219<br>Signed using mobile<br>With Signing Authentication via DocuSign password<br>With Signing Reasons (on each tab):<br>I approve this document	Sent: 10/21/2022 5:58:56 AM<br>Viewed: 10/21/2022 6:07:25 AM<br>Signed: 10/21/2022 6:08:00 AM

Electronic Record and Signature Disclosure:
Accepted: 10/21/2022 6:07:25 AM
ID: 632434e0-ae6d-40b6-9c1f-41ac0ff93bb4

Signer Events	Signature	Timestamp
Seth Bennett<br>sbennett@ctifacts.com<br>Biostatistician II<br>CTI Clinical Trial and Consulting Services<br>Security Level: Email, Account Authentication (Required)	Seth Bennett<br><br>Signature Adoption: Pre-selected Style<br>Signature ID:<br>1D9E9FC1-398F-4508-9B4F-8325E01BB911<br>Using IP Address: 208.102.95.169<br><br>With Signing Authentication via DocuSign password<br>With Signing Reasons (on each tab):<br>I am the author of this document	Sent: 10/21/2022 5:58:56 AM<br>Viewed: 10/21/2022 6:19:43 AM<br>Signed: 10/21/2022 6:20:10 AM

Electronic Record and Signature Disclosure:
Not Offered via DocuSign

In Person Signer Events	Signature	Timestamp

Editor Delivery Events	Status	Timestamp

Agent Delivery Events	Status	Timestamp

Intermediary Delivery Events	Status	Timestamp

Certified Delivery Events	Status	Timestamp

Carbon Copy Events	Status	Timestamp

Witness Events	Signature	Timestamp

Notary Events	Signature	Timestamp

Envelope Summary Events	Status	Timestamps
Envelope Sent	Hashed/Encrypted	10/21/2022 5:58:56 AM
Certified Delivered	Security Checked	10/21/2022 6:19:43 AM
Signing Complete	Security Checked	10/21/2022 6:20:10 AM
Completed	Security Checked	10/21/2022 8:12:43 AM

Payment Events	Status	Timestamps

Electronic Record and Signature Disclosure

ELECTRONIC RECORD AND SIGNATURE DISCLOSURE

From time to time, CTI Clinical Trial and Consulting Services (we, us or Company) may be required by law to provide to you certain written notices or disclosures. Described below are the terms and conditions for providing to you such notices and disclosures electronically through the DocuSign system. Please read the information below carefully and thoroughly, and if you can access this information electronically to your satisfaction and agree to this Electronic Record and Signature Disclosure (ERSD), please confirm your agreement by selecting the check-box next to 'I agree to use electronic records and signatures' before clicking 'CONTINUE' within the DocuSign system.

Electronic Signature Responsiblities

You agree that your electronic signature is the equivalent of a hand-written signature and that you are responsible for actions initiated using your electronic signature. You agree to report unauthorized usage to CTI upon discovery of such usage via email to esign@ctifacts.com.

Getting paper copies

At any time, you may request from us a paper copy of any record provided or made available electronically to you by us. You will have the ability to download and print documents we send to you through the DocuSign system during and immediately after the signing session and, if you elect to create a DocuSign account, you may access the documents for a limited period of time (usually 30 days) after such documents are first sent to you. After such time, if you wish for us to send you paper copies of any such documents from our office to you, you will be charged a per-page fee. You may request delivery of such paper copies from us by following the procedure described below.

Withdrawing your consent

If you decide to receive notices and disclosures from us electronically, you may at any time change your mind and tell us that thereafter you want to receive required notices and disclosures only in paper format. How you must inform us of your decision to receive future notices and disclosure in paper format and withdraw your consent to receive notices and disclosures electronically is described below.

Consequences of changing your mind

If you elect to receive required notices and disclosures only in paper format, it will slow the speed at which we can complete certain steps in transactions with you and delivering services to you because we will need first to send the required notices or disclosures to you in paper format, and then wait until we receive back from you your acknowledgment of your receipt of such paper notices or disclosures. Further, you will no longer be able to use the DocuSign system to

receive required notices and consents electronically from us or to sign electronically documents from us.

All notices and disclosures will be sent to you electronically

Unless you tell us otherwise in accordance with the procedures described herein, we will provide electronically to you through the DocuSign system all required notices, disclosures, authorizations, acknowledgements, and other documents that are required to be provided or made available to you during the course of our relationship with you. To reduce the chance of you inadvertently not receiving any notice or disclosure, we prefer to provide all of the required notices and disclosures to you by the same method and to the same address that you have given us. Thus, you can receive all the disclosures and notices electronically or in paper format through the paper mail delivery system. If you do not agree with this process, please let us know as described below. Please also see the paragraph immediately above that describes the consequences of your electing not to receive delivery of the notices and disclosures electronically from us.

How to contact CTI Clinical Trial and Consulting Services:

You may contact us to let us know of your changes as to how we may contact you electronically, to request paper copies of certain information from us, and to withdraw your prior consent to receive notices and disclosures electronically as follows:
Please send an email to esign@ctifacts.com

To advise CTI Clinical Trial and Consulting Services of your new email address

To let us know of a change in your email address where we should send notices and disclosures electronically to you, you must send an email message to us at esign@ctifacts.com and in the body of such request you must state: your previous email address, your new email address.

If you created a DocuSign account, you may update it with your new email address through your account preferences.

To request paper copies from CTI Clinical Trial and Consulting Services

To request delivery from us of paper copies of the notices and disclosures previously provided by us to you electronically, you must send us an email to esign@ctifacts.com and in the body of such request you must state your email address, full name, mailing address, and telephone number.

To withdraw your consent with CTI Clinical Trial and Consulting Services

To inform us that you no longer wish to receive future notices and disclosures in electronic format you may:

i. decline to sign a document from within your signing session, and on the subsequent page, select the check-box indicating you wish to withdraw your consent, or you may;

ii. send us an email to esign@ctifacts.com and in the body of such request you must state your email, full name, mailing address, and telephone number.

Required hardware and software

The minimum system requirements for using the DocuSign system may change over time. The current system requirements are found here: https://support.docusign.com/guides/signer-guide-signing-system-requirements.

Acknowledging your access and consent to receive and sign documents electronically

To confirm to us that you can access this information electronically, which will be similar to other electronic notices and disclosures that we will provide to you, please confirm that you have read this ERSD, and (i) that you are able to print on paper or electronically save this ERSD for your future reference and access; or (ii) that you are able to email this ERSD to an email address where you will be able to print on paper or save it for your future reference and access. Further, if you consent to receiving notices and disclosures exclusively in electronic format as described herein, then select the check-box next to 'I agree to use electronic records and signatures' before clicking 'CONTINUE' within the DocuSign system.

By selecting the check-box next to 'I agree to use electronic records and signatures', you confirm that:

- You can access and read this Electronic Record and Signature Disclosure; and
- You can print on paper this Electronic Record and Signature Disclosure, or save or send this Electronic Record and Disclosure to a location where you can print it, for future reference and access; and
- Until or unless you notify CTI Clinical Trial and Consulting Services as described above, you consent to receive exclusively through electronic means all notices, disclosures, authorizations, acknowledgements, and other documents that are required to be provided or made available to you by CTI Clinical Trial and Consulting Services during the course of your relationship with CTI Clinical Trial and Consulting Services.
